# Autophagic impairment in sleep–wake circuitry is linked to sleep loss at the early stages of Alzheimer’s disease

**DOI:** 10.1186/s13024-025-00877-2

**Published:** 2025-09-26

**Authors:** Christopher Daniel Morrone, Arielle A. Tsang, W. Haung Yu

**Affiliations:** 1 Brain Health Imaging Centre, Centre for Addiction and Mental Health, 250 College Street, Toronto, Canada; 2 Department of Biological Sciences, University of Toronto Scarborough, Toronto, ON Canada; 3 Department of Pharmacology and Toxicology, University of Toronto, Toronto, Canada

## Abstract

**Background:**

Proteostasis, in particular the impairment of autophagic activity, is linked to sleep dysregulation and is an early sign of dementias including Alzheimer’s disease (AD). This coupling of events may be a critical alteration driving proteinopathy and AD progression. In the present study, we investigated sleep–wake and memory regulating neurons for vulnerability to autophagic impediment, and related these findings to progression of the sleep and cognitive phenotype.

**Methods:**

Using the double knock-in AD mouse model, *App*^NL−G−F^x*MAPT*, we examined phenotypic and pathological alterations at several timepoints and compared to age-matched single knock-in *MAPT* mice. Spatial learning, memory and executive Function were investigated in the Barnes maze. Sleep was investigated by 24-h locomotor activity and EEG. Immunostaining for autophagic, neuronal and pathological markers was conducted in brain regions related to memory (hippocampus, prefrontal cortex, entorhinal cortex) and the sleep–wake cycle (hypothalamus, locus coeruleus). Hippocampal electrophysiological recordings were conducted to probe neuronal Function during object investigation. A 3-day sleep disruption was conducted in *MAPT* mice to investigate autophagic changes following sleep loss. Autophagy was activated in *MAPT* mice with trehalose to probe effects on sleep recovery.

**Results:**

We identified that disrupted sleep occurred from early-stages in *App*^NL−G−F^x*MAPT* mice, that sleep declined over age, and sleep deficits preceded cognitive impairments in late-stages. Cytoplasmic autophagic impediment in hypothalamic and locus coeruleus sleep–wake neurons occurred in early-stage *App*^NL−G−F^x*MAPT* mice, prior to significant β-amyloid deposition in these regions, with a failure of lysosomal flux over disease progression. Autophagic changes in the hippocampus and cortex at early-stage were predominantly in processes and less frequently associated with the lysosome. Plaque-associated autophagic and lysosomal accumulations were frequent from the early-stage. Sex differences in the AD phenotype were prominent, including greater cognitive decline in males than females, linked to increased proteostasis burden in EC layer II neurons and hippocampal tau in the late-stage. Conversely, sleep impairments were more rapid in females including less REM sleep recovery than males, along with greater autophagic burden in hippocampal processes of female *App*^NL−G−F^x*MAPT* mice. We probed the sleep-cognition linkage demonstrating hippocampal electrophysiological slowing during cognitive processing in mid-stage *App*^NL−G−F^x*MAPT* mice, prior to cognitive decline. We provide evidence for a positive feedback loop in the autophagic-sleep relationship by demonstrating that disrupted sleep in *MAPT* mice led to arrhythmic sleep patterns and accumulations of autophagic aggregates in the hippocampus and hypothalamus, similar to as was seen in the early Alzheimer’s phenotype. We further probed the autophagy-sleep linkage by treating *MAPT* mice with trehalose to activate autophagy and demonstrate an improvement in sleep recovery following a sleep disruption.

**Conclusions:**

These findings demonstrate the vulnerability of sleep-regulating neurons to proteostatic dysfunction and the sleep-autophagy linkage as an early, and treatable, Alzheimer’s disease mechanism.

**Graphical Abstract:**

Morrone *et al* provide evidence for the linkage between sleep and autophagic disruptions in Alzheimer’s disease (AD) progression. At early AD stages, sleep-wake regulating neurons in the hypothalamus and locus coeruleus exhibit increased cytoplasmic inclusions concomitant with the onset of sleep disturbances. Early-stage autophagic aggregates in the hippocampus appear more prominently in neuronal processes and in the cortex linked to plaques. This pathology worsens over AD progression, including advanced sleep and cognitive deficits, autophagic aggregates in entorhinal cortex-hippocampus projecting neurons. Disrupting sleep in control mice mimics the hippocampal, hypothalamic and sleep patterns impairments observed in early-stage AD, and therapeutic activation of autophagy improves sleep recovery. See also Table 1 for a summary of changes along with sex differences in autophagy and behavioral readouts.

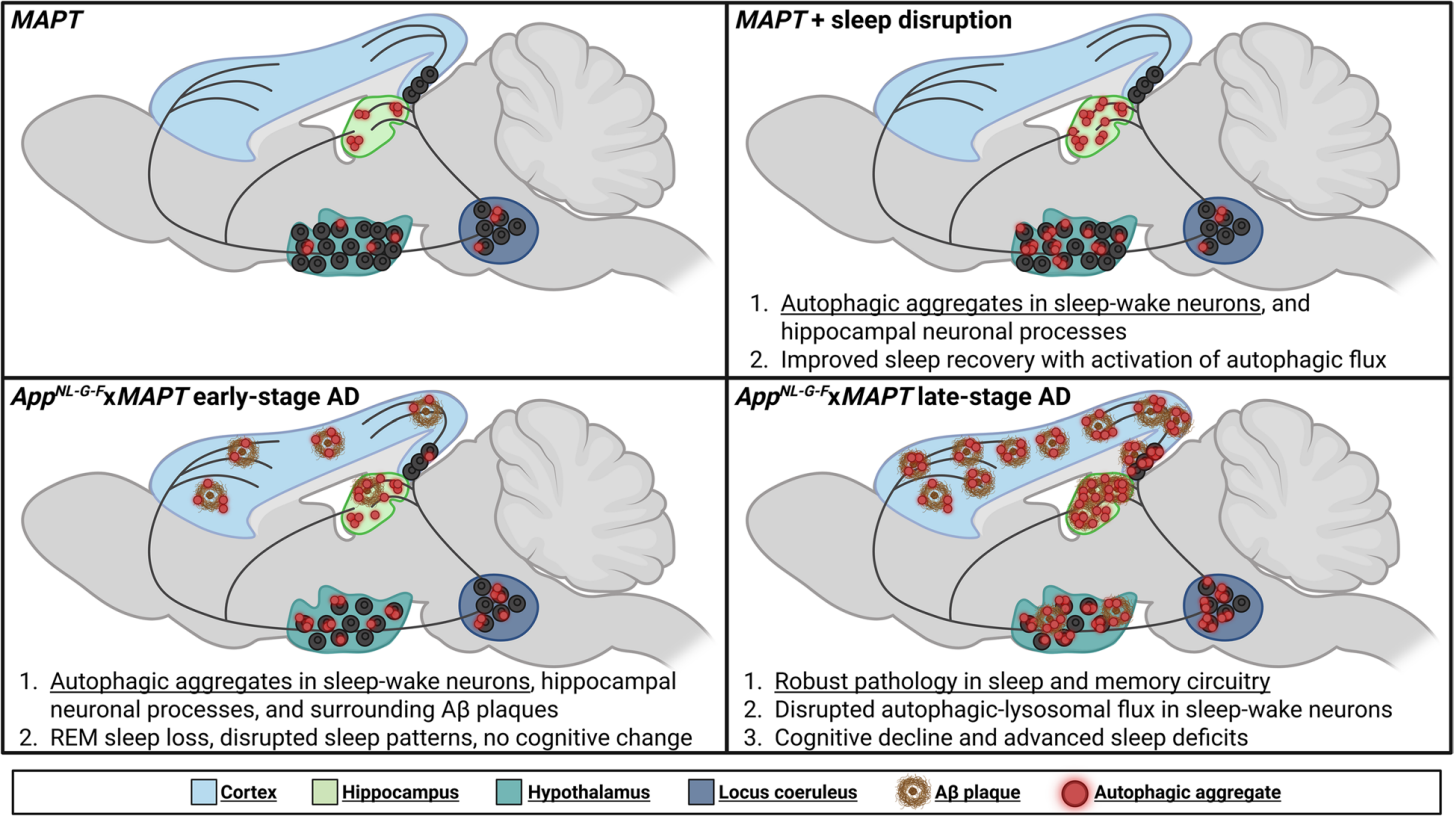

**Supplementary Information:**

The online version contains supplementary material available at 10.1186/s13024-025-00877-2.

## Introduction

At the core of Alzheimer’s disease (AD) progression is proteinopathy. Classically this referred to accumulation of β-amyloid (Aβ) and tau pathologies, though recent evidence indicates the prevalence of other aggregate-prone proteins including α-synuclein and TDP-43 [1–6]. Common to these neurodegenerative, aggregate-prone species is an overwhelming of cellular proteostasis, leading to failed protein degradation. Autophagy in particular is impacted in AD, with proteins targeted to the autophagosome failing to degrade and accumulating in neurons [7, 8]. Reduced axonal transport and lysosomal fusion leads to abundant uncleared protein, contributing to neurodegeneration and to pathological spread through the brain [9–11]. There is an urgency to understand regional and neuronal vulnerabilities to autophagic impediment, and treatable modulators of these disease mechanisms.

One such factor is sleep impairment, a common occurrence in people with AD and seen in the majority of brain disorders. Prodromal sleep disruptions confer a 3.78 × risk for exhibiting preclinical AD biomarkers, and even a single night of sleep disruption can increase Aβ and tau levels [12–15]. Loss in the quantity and quality of sleep, particularly slow wave sleep and rapid eye movement sleep (REM), associates with AD cognitive impairments and pathological development. There is an intimate connection between sleep and proteostasis, in which impairments in these processes accelerate the other and neurodegenerative proteinopathy in a positive-feedback-loop [11]. In particular, autophagic flux is related to sleep and circadian function [11], though the regions and neurons sensitive to autophagic impediments in the neurodegenerative environment and in relation to sleep and cognitive changes, remain to be elucidated.

In this study, we utilized a double knock-in (DKI) mouse model of AD bearing the human amyloid precursor protein (APP) and microtubule associated protein tau (MAPT) transgenes: *App*^NL−G−F^x*MAPT*. *App*^NL−G−F^x*MAPT* DKI mice generate pathology from 3 APP mutations (Swedish, Iberian, Arctic) to increase the cleavage to and pathogenicity of Aβ. Human *MAPT* is not mutated in the model, yet the presence of the 6 tau isoforms present in humans (vs. 3 in mice) better recapitulates Aβ-tau interactions and tauopathy in AD patients [16, 17]. Single knock-in *MAPT* mice were utilized as a control to model endogenous, non-pathological tau effects as a comparator to Aβ pathology and synergistic Aβ-tau effects in *App*^NL−G−F^x*MAPT*s, as seen in this model and in *App*^NL−G−F^ mice crossed to the *MAPT* overexpression model P301S [16, 18]. Knock-in expression patterns is an additional advantage in *App*^NL−G−F^x*MAPT*s to allow normal cellular proteostasis in early age without impeding these systems from transgene overexpression.

Three ages were chosen in the *App*^NL−G−F^x*MAPT* model based on amyloid plaque staging resulting with the *App*^NL−G−F^ mutations, where plaque onset occurs between 2-to-4-months of age [17]. *First,* “early-stage” 4-months represents Thal phase 1–2 with significant cortical deposition, yet sparse and diffuse plaques in the hippocampus. *Second*, “mid-stage” 8-months represents Thal phase 2–3 with much greater plaque burden than 4-months, and subcortical deposition in the hypothalamus for example. *Third,* “late-stage” 12-months represents Thal phase 3–5 with the hippocampus approaching the cortical level especially with newer plaque formations, significant striatal and hypothalamic deposition, and the presence of brain stem Aβ plaques in locus coeruleus and adjacent regions [3, 17, 19]. Most reports indicate preservation of spatial and working memory until 8-to-12-months of age in these mice [20–25], though there are indications of altered memory modalities as early as 6-months [17, 26].

In this study, we characterize sleep profiles and cognitive changes from early-to-late-stage AD pathology, and identify behavioral and electrophysiological changes that precede cognitive decline. Furthermore, we identify neurons and brain regions sensitive to autophagic impediments in relation to the behavioral phenotype, in order to elucidate the importance of the sleep-autophagy relationship in AD, and inform on potential therapeutic interventions. We then probe the sleep-to-autophagy interaction utilizing sleep disruption and autophagy activation to model effects of sleep loss on autophagy, and of activating autophagic flux on sleep.

## Methods

### Animals

All mouse experiments were conducted in accordance with the ethical standards of the Canadian Council on Animal Care guidelines and approved by the Animal Care Committee of CAMH (Protocol #850). Homozygous *App*^NL−G−*F*^x*MAPT* DKI and *MAPT* single knock-in mice were bred in-house (original lines established, characterized and available through Dr. Takaomi Saido: [16, 17]), and housed in a 12-h light:dark-cycle with ad libitum access to chow and water. All mice were on a C57Bl6J background. Humanization of the *MAPT* gene in mice maintains physiological tau function, and therefore *MAPT* single knock-in mice were utilized as a control group in the present study, appropriate for the synergistic Aβ-tau effects in *App*^NL−G−*F*^x*MAPT* DKI mice [16]. Six cohorts of mice were utilized in this study: *1)* longitudinal cognitive and locomotor activity assessments (*n* = 43, 10–11/sex/genotype at 4-, 8- and 12-months); *2)* longitudinal EEG/EMG (*n* = 20 total, 5/sex/genotype at 4- and 12-months); *3)* pathology on brain tissue (*n* = 18, 3/sex/genotype/age); *4)* hippocampal depth electrode (*n* = 17, 3–5/sex/genotype at 8-months); *5) MAPT* Ctrl vs. 3-day sleep disruption (3DSD; *n* = 18, 4–5/sex/condition at 10-to-12-months of age); *6) MAPT*-sucrose vs. *MAPT*-trehalose treated mice (*n* = 20, 5/sex/treatment at 12-months). Exact *n* per analysis is provided in figures, figure legends and results text.

### Barnes maze

Barnes maze cognitive testing was conducted repeated at 4-, 8- and 12-months of age in the same cohort of mice, by similar methods as we have previously reported [27]. Briefly, a circular field was utilized with 20 holes (1 escape box) along the outside (92 cm diameter, Maze Engineers), and an overhead camera acquired trials in EthoVision XT (Noldus) software. After a habituation day, mice were tested twice per day for 4 days in learning trials (3 min trials, 2-h inter-trial interval) for memory of the escape box, with an aversive overhead light and spatial cues oriented around the testing room. The memory probe was conducted in one 3-min trial 2 days later, with the escape box blocked. Reversal trials were run starting the next day in the same manner as the learning trials except that the escape box location was rotated 180°. For learning and reversal trials, latency to the escape box (s) and number of errors were calculated in EthoVision per trial and the two trials were averaged per day. For trials in which the mouse did not find the escape box within 3 min, 20 errors were added. For the probe, the time spent in the target quadrant (%), and a search strategy score were calculated in EthoVision. Barnes maze data in Fig. 2 is presented as a pooled average across trial and reversal trial days to assess age*genotype*sex effects; trial day breakdowns are presented in Supplementary Fig. 4. The search strategy score involved assessment of direct and indirect zone transitions to the escape box, centre crossings, time spent searching target and non-target quadrants, and velocity to create a composite search strategy score, and then was binned in 30 s intervals and averaged across the trial. This analysis represents direct and corrected strategies (score range ~ 3–5), long correction and focused search (score range ~ 1–3), serial search (score range ~ 0–1) and random (score range < 0). Search strategy distinctions and weighting were determined based off of previous publications [28, 29].

### PhenoTyper locomotor activity and ADLs

Approximately 1-week after finishing the Barnes maze at each age, the same mice were tested in PhenoTyper home-cages (Noldus) over a 24-h period with extended habituation and post-testing time for nesting time-points at 42-h, as we previously described [27]. Mice were placed in the cages (single-caged) 3-h before the start of the dark-cycle to allow habituation before data collection. Nest building was scored manually at 18-, 24- and 42-h as per [30], from untouched (score of 1), to a fully-formed nest (score of 5). Locomotor activity was recorded on an overhead camera and analyzed in EthoVision XT10. Data was split into 12-h dark- and light-cycle segments (or 2-h segments within the light-cycle), binned by 10-s intervals, and analyzed for locomotor velocity (cm/s). “Attempted sleep” states were quantified by 4 consecutive data points (40 s) with a velocity < 0.1 cm/s [27, 31], and the percentage of time spent sleeping was then calculated for the dark- and light-cycle.

### Sleep disruption

For the 6-h or 3-day sleep disruption, mice were single-caged in PhenoTyper cages, and a tone (2,300 Hz, 80 dB) and white light were generated to disrupt sleep within each cage throughout the 6- (12 pm-6 pm, starting 5 h after the light-cycle onset) or 72-h period (starting 2 h after the light-cycle onset). Tone (length: 10–30 s, interval: 30–180 s) and light (length: 20–60 s, interval: 30–180 s) length and interval were randomized to prevent habituation, similar to our previous methods [27]. Locomotor activity was recorded during the 3-day period as described above. Activity patterns in control mice were simultaneously recorded in PhenoTypers in a separate testing room from the 3DSD mice. Control and 3DSD mice were immediately sacrificed at the end of the 3-day period at a consistent time in the light–dark-cycle: 2–4 h after the start of the light-cycle.

### EEG and electrophysiology recordings, spectral analysis and sleep staging

For EEG/EMG analyses and hippocampal electrophysiology, prefabricated headcaps (Pinnacle Technology Inc.) were utilized and surgeries were performed as we previously described [27]. Briefly, mice were anesthetized with isoflurane (5% induction, 1–2% maintenance), provided analgesic (Metacam) and local anesthetic (Bupivacaine) to the incision site, and placed on a stereotaxic frame. An incision was made to expose the skull. For EEG/EMG headcaps (8201-SS, Pinnacle Technology Inc.) mice were approximately 3.5 months (Fig. 3) or 9 months (Fig. 10) of age at time of surgery: 4 electrode screws were implanted over the left and right Hemispheres with 2 anterior (Bregma –2–2.5 mm AP, 1.5 ML) and 2 posterior (Bregma 3.5–4 mm AP, 1.5 ML) screws. For the hippocampal depth electrode (Fig. 8; 8201-DEP-SS, Pinnacle Technology Inc.), mice were approximately 7.5 months at the time of surgery: a drill was utilized to make a small hole (Bregma −1.5 mm AP, 0.5 ML), the electrode was slowly inserted to a depth of 2 mm, and screws were utilized to secure the Headcap. Silver epoxy was used to adhere screws to the Headcaps, and dental acrylic to seal and protect the headcaps. Mice were allowed to recover for at least 1 week prior to recordings.

A wireless, battery-operated potentiostat was plugged into headcaps at the time of recording with data acquired, digitized and amplified at the potentiostat prior to being transferred to a computer via Bluetooth to Sirenia Acquisition v2.2 (Pinnacle Technology Inc) software. Recordings were sampled at 1024 Hz, 100 × gain, with a 0.5 Hz high-pass filter for EEG and 10 Hz high-pass filter for EMG; a 500 Hz low-pass filter was applied to all channels. An anterior EEG electrode was utilized for sleep-staging and was normalized to a posterior electrode to minimize noise. The EMG signal output was generated as the difference between the two wires. As previously shown, it is possible to do longitudinal recordings [27], EEG/EMG recordings were conducted at 4- and then at 12-months of age in PhenoTyper home-cages over a 24-h period. Sleep staging was conducted in Sirenia Sleep v2.2 (Pinnacle Technology Inc) similar to our previous methods [27]. Fast Fourier Transform (FFT) with a Hann windowing function was utilized to transform data from time to frequency. EEG spectral power (µV^2^/Hz; anterior electrode) was generated for delta (0.5–4 Hz), theta (4–8 Hz), alpha (8–13 Hz) and beta (13–30 Hz) bands, total power (0.5–500 Hz) as well as EMG power (50–150 Hz), in 4-s epochs. Each 4-s epoch within the 12-h dark- and light-cycles were scored as wake, REM and NREM using a semi-automatic method. Briefly, cluster scoring was utilized to define a sleep–wake threshold by EMG power (high EMG = wake), and within the sleep cluster, a REM-NREM threshold was defined using the theta:delta power ratio, with delta dominant sleep indicating NREM and theta dominant sleep indicating REM (see Morrone et al. [27]). Accuracy of the cluster scoring was validated manually for each mouse scored. This analysis leaves ~ 5–10% of the transitional epochs unscored which were then scored manually.

Hippocampal electrophysiological field recordings (1024 Hz sample rate, 100 × gain, 0.5 Hz high-pass, 500 Hz low-pass) were conducted at 8-months of age during an object investigation task. Hippocampal electrophysiological data was acquired, digitized and amplified at the potentiostat, then transferred to Sirenia software via Bluetooth. Potentiostats were plugged-in and mice were allowed to habituate to the testing arena (30 × 30 cm). One hour after habituation, two of the same object (cell culture flask filled with bedding (10.3 × 4.5x2.5 cm LxWxH) or Lego tower (10.5 × 4.7x4.7 cm LxWxH) [32]) were placed in the testing arena. Video and electrophysiological data were recorded concurrently on the same computer. FFT followed by hippocampal power generation in 4-s bands was conducted as described for the EEG, and time aligned by computer clock time to during “object investigation” or not (calculated in EthoVision XT10) in excel. Data alignment was with the time-stamped hippocampal power (generated in 4-s epochs) and mouse location relative to the object in the arena (average over 4-secs). Total power (0.5–500 Hz) was reported in the habituation (no objects). For the object investigation, delta (0.5–4 Hz), theta (4–8 Hz), alpha (8–13 Hz) and beta (13–30 Hz) power was expressed as a ratio of averaged epochs of “object investigation” vs. “non-object investigation” during the same trial, to detect electrophysiological changes during learning and exploratory behavior. Frequency spectra were generated in Sirenia Sleep in representative mice to delineate power averaged across “object investigation” and “non-object investigation” epochs.

### Trehalose and sucrose treatment

Mice in cohort #6 (Fig. 10) underwent an oral treatment of 2% trehalose to activate autophagy, or 2% sucrose as a disaccharide control, both administered ad libitum in the drinking water; method adapted from previous work [33–35]. Treatment onset was at 10-months of age and continued until and throughout the testing period at ~ 12-to-13-months of age, with treated water changed weekly. Mice of both cohorts drank at least 5 mL/mouse/day, in line with regular daily water intake.

### Immunostaining

Mice were anesthetized with an overdose of avertin and transcardially perfused with Heparinized 1× phosphate buffered saline (PBS), then with 4% paraformaldehyde. Brains were incubated overnight in 4% paraformaldehyde, then washed and stored in 30% sucrose at 4 °C. Coronal sections were collected at 40 µm on a sliding microtome (Leica SM2000R) through PFC (~ Bregma 0 to −0.20 mm), hypothalamus (POA: ~ Bregma 0 to −0.20 mm; LH: ~ Bregma −1.30 to −1.50), hippocampus (~ Bregma −1.30 to −3.20), lateral EC (~ Bregma −3.10 to −3.40), and LC (~ Bregma −5.30 to −5.50), and stored at −20 °C in tissue cryoprotectant.

Immunohistochemistry was conducted for Aβ plaques with the 6F/3D antibody, adapted from previous methods [36]. Briefly, free-floating sections were washed in 1xPBS, incubated for 30 min in 1% hydrogen peroxide to block endogenous peroxidases, washed, underwent antigen retrieval with 70% formic acid for 5 min, were washed, then blocked (5% horse serum, 0.2% Triton-X100, 0.2% bovine serum albumin (BSA)) for 1 h. Following blocking, sections were incubated at room temperature overnight with the mouse anti-6F/3D antibody (1:400; Dako, M0872) in 1xPBS with 0.2% Triton-X100 and 0.2% BSA. The next day sections were washed then underwent a 1.5-h secondary incubation (biotinylated horse anti-mouse IgG, 1:400; ABC kit, Vector Laboratories, PK-4002) in 1xPBS with 0.2% Triton-X100 and 0.2% BSA. Following washes, sections were incubated for 1-h with reagent A and B from the ABC kit (both 1:200; PK-4002), washed again, and developed (~ 7 min) with a 3,3'-diaminobenzidine (DAB) horseradish peroxidase substrate kit using nickel chloride for a gray-black signal (Vector Laboratories, SK-4100). Sections were then washed, mounted on a microscope slide and dehydrated: 5-min 70% ethanol, 5-min 95% ethanol, 5-min 100% ethanol, 10-min xylene. Slides were then cover-slipped in Cytoseal mounting media (Epredia). Representative hippocampal images were captured at 10 × magnification using an Olympus VS200 slide scanner.

Immunofluorescence was conducted by standard methods similar to previous work from the authors [28, 37]. Primary antibodies for molecular markers included monoclonal rabbit anti-p62 (1:400; Abcam, ab109012), monoclonal rat anti-LAMP1 (1:500, Biolegend, 121,602), and polyclonal rabbit anti-CCP3 (1:100; Cell Signaling Technology, 9661). Primary antibodies for cellular markers included polyclonal guinea pig anti-NeuN (1:500; Millipore Sigma, ABN90), monoclonal mouse anti-NeuN (Supplementary Fig. 2 only; 1:500; Millipore Sigma, MAB377), monoclonal mouse anti-GAD67 (1:1000; Millipore Sigma, MAB5406), monoclonal mouse anti-Orexin A (1:200; Santa Cruz Biotechnology; sc-80263), and monoclonal mouse anti-MAP2 (1:500; Millipore Sigma, M1406). Primary antibodies for pathological markers included monoclonal mouse anti-β-amyloid (6F/3D, 1:200 for immunofluorescence; Dako, M0872), monoclonal mouse anti-PHF1 (1:250; courtesy of Dr. Peter Davies), and monoclonal mouse anti-CP13 (1:250; courtesy of Dr. Peter Davies). For stains that did not include 6F/3D or PHF1, sections were washed in 1xPBS, blocked (2% goat serum, 1% BSA, 0.1% Triton-X100 in 1xPBS), and incubated with primary antibody in the blocking solution, at 4 °C. Different blocking solutions were utilized for stains containing LAMP1 (5% goat serum, 1% BSA, 0.1% Triton-X100 in 1xPBS) and for CCP3 (10% goat serum, 1% BSA, 0.3% Triton-X100 in 1xPBS). The following day sections were washed and then incubated with appropriately targeted fluorescent secondary antibodies (all 1:200, see Supplementary Table 1 for specific antibodies) diluted in the blocking solution, at room temperature for 2-h. Three iterations of immunofluorescent staining were conducted for specific antibody probes.

#### Iteration 1

For 6F/3D staining, the same procedures were followed with addition of an antigen retrieval step prior to blocking: 70% formic acid for 5 min.

#### Iteration 2

PHF1 and CP13 immunostaining included washes and incubations using 1 × tris buffered saline (TBS). Sections were washed, blocked in 5% milk and 0.25% Triton-X100 in 1xTBS, then incubated with primary antibodies overnight at 4 °C in 5% milk in 1xTBS. On day 2, the washes were in 1xTBS containing Triton-X100 (0.05%) until just before mounting (or before Thioflavin-S if included) when sections were switched back to 1xTBS washes. Sections were washed then incubated for 2-h with secondary antibodies including biotinylated goat anti-mouse IgG1 (1:80; Invitrogen, A10519) to amplify the PHF1 or CP13 signal, and fluorescent secondary antibodies for any additional targets. Sections were washed then incubated with streptavidin Alexa Fluor 647 (1:200; Invitrogen S32357) for 2-h at room temperature.

#### Iteration 3

For Thioflavin-S (Sigma-Aldrich; T1892), incubations were after the secondary and prior to DAPI: 7 min in Thioflavin-S (1% wt/volume in ddH2O), followed by 2 × 5-min 70% ethanol washes before returning sections to the wash buffer. For each type of immunofluorescent stain, sections were then incubated with DAPI (1:5000) for 10 min, washed, then mounted and cover-slipped with ProLong™ Gold antifade mounting media (Invitrogen).

### Immunofluorescence analysis

**Fig. 1:**
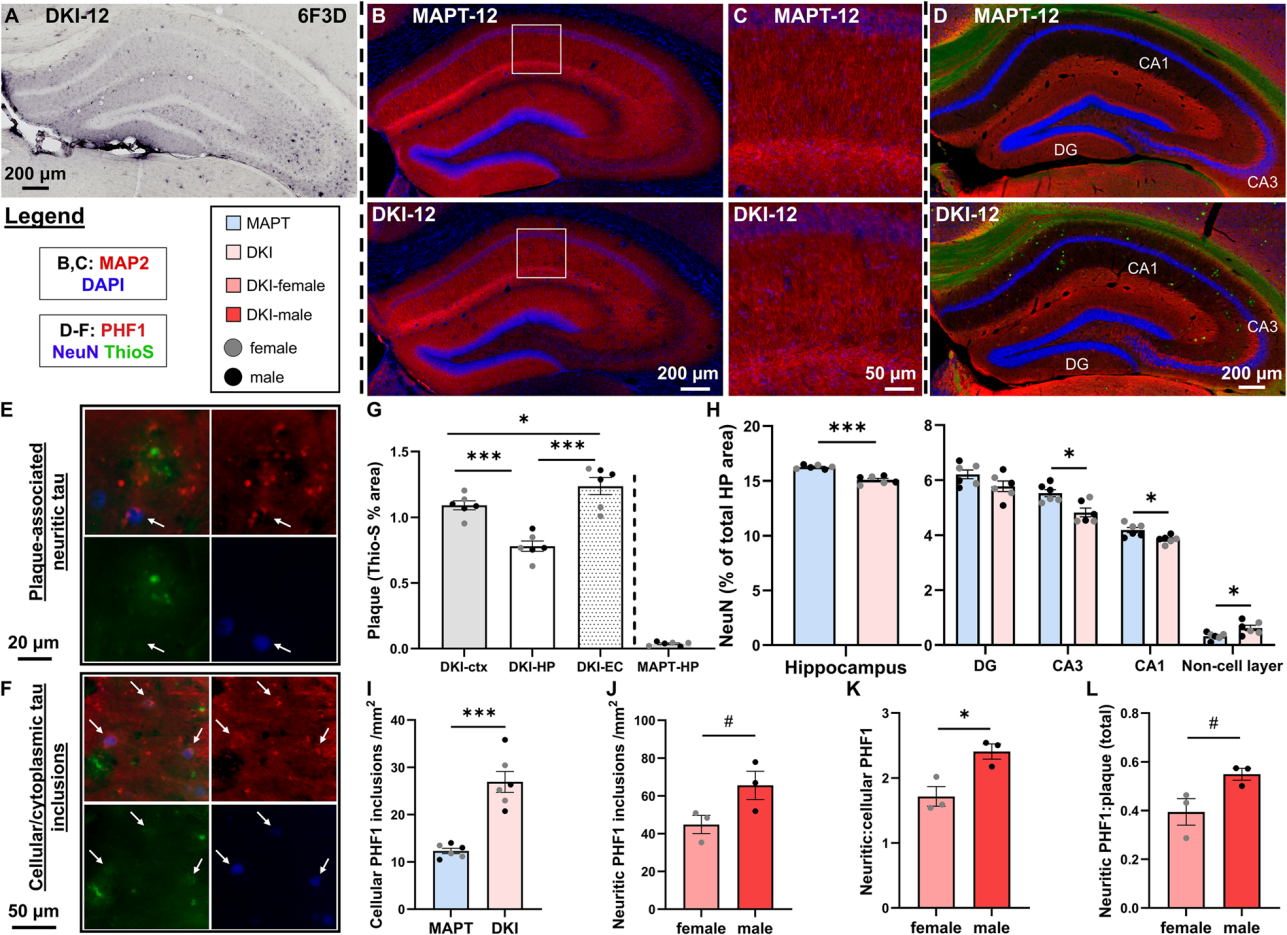
Hippocampal neurodegeneration in late-stage *App*^NL−G−F^x*MAPT* mice, greater tau pathology in males. **A** Representative hippocampal Aβ plaque (6F/3D, black) pathology in 12-month old *App*^NL−G−F^x*MAPT* (DKI) mice (DKI-12). **B**,**C** Relative to 12-month old MAPT (MAPT-12) mice, *App*^NL−G−F^x*MAPT* mice exhibit degenerative hippocampal neuronal processes (MAP2, red), especially around plaque formations. **D** Representative PHF1 (red), NeuN (blue) and ThioS (green) staining in *MAPT* and *App*^NL−G−F^x*MAPT* mice demonstrating Aβ and tau pathologies and thinning of CA1 and CA3 pyramidal cell layers in the 12-month *App*^NL−G−F^x*MAPT* mice. No neuronal loss was observed at earlier ages (Supplementary Fig. 1). **E** Aβ plaques (ThioS, green) have extensive tau + dystrophic neurites (PHF1, red). **F** Cellular, non-plaque-associated tau inclusions (arrows) are also prevalent in *App*^NL−G−F^x*MAPT* mice. **G** Quantification of regional area covered by Aβ determined greater cortical (neocortex and entorhinal cortex – EC) vs. hippocampal area covered (HP), though the hippocampus has more frequent small, putatively new plaque formations (Supplementary Fig. 1). **H** Quantification of hippocampal NeuN in 12-month *App*^NL−G−F^x*MAPT* mice determining significantly less NeuN signal in total hippocampus, primarily from CA1 and CA3 cell layers, no change in the dentate gyrus (DG), and more non-cell layer signal. **I** Cellular PHF1 + aggregates were significantly increased in *App*^NL−G−F^x*MAPT* vs. *MAPT* mice. **J** Neuritic PHF1 + aggregates trended to an increase in males vs. females in late-stage *App*^NL−G.−F^x*MAPT*s. **K** The ratio of neuritic:cellular PHF1 inclusions was significantly higher in males. **L** Male *App*^NL−G.−F^x*MAPT*s also exhibit a trend to more PHF1 per plaque. Data are presented as mean ± SEM; *n* = 3/sex/genotype. #*P* < 0.10, *P < 0.05, **P < 0.01, ***P < 0.001. Statistical analysis was conducted using a one-way ANOVA, Holm-Šídák post-hoc (**G**), multiple unpaired t-tests, Holm-Šídák correction (**H**), or unpaired t-test (**I**-**L**); see Supplementary Table 2 for complete statistics

Analysis and representative images were collected at 10x (Fig. 1 (except B, C, E and F), Fig. 9D-F, Supplementary Fig. 1) or 20x (all others except Supplementary Fig. 8) magnification using an Olympus VS200 slide scanner; representative images in Supplementary Fig. 8 were collected at 40 × magnification on an Olympus Disk-Spinning Unit confocal microscope. Thioflavin-S images were binarized and analyzed for staining density (% area, # of plaques) and binned into plaque sizes (10–100, 100–200, 200–300 and > 300 µm^2^) for hippocampus, neocortex and EC (2 sections, both hemispheres, per region per mouse). Thioflavin-S + plaques were counted in the hypothalamus (normalized to area) and in the locus coeruleus (normalized to section); sampling: 1 section, both hemispheres, per mouse. PHF1 plaque-associated (visualized with Thioflavin-S positive plaques) and non-plaque associated inclusions were quantified in ImageJ for total hippocampus, DG, CA3 and CA1, normalized to regional or subregional area (2 sections spaced 1 in 14, both hemispheres, per mouse). Plaque-associated inclusions (neuritic) were also expressed as a ratio to non-plaque associated inclusions (cellular) and to total plaque count (including small Aβ + aggregates). Hippocampal NeuN images (3 sections spaced 1 in 14, both hemispheres, per mouse) were binarized and automatically analyzed for staining density (“Analyze Particles” function) in ImageJ for the total hippocampus. Area of NeuN + staining density in DG, CA3 and CA1 cell layers was normalized to the total hippocampal area. The remainder of the total hippocampal area minus the 3 cell layers calculated the non-cell layer portion.

Analysis for p62 in combination with NeuN, DAPI, PHF1, CP13, ThioS, 6F/3D, MAP2, Orexin A, LAMP1, and/or GAD67 was conducted in ImageJ. Hippocampal and PFC p62 analysis involved quantification of clusters (10 or more individual p62 aggregates within a 50–100 µm radius). Hippocampal p62 clusters were counted as plaque-associated (by surrounding PHF1 positivity) or non-plaque associated. EC p62 analysis involved quantification of NeuN + cells containing specifically p62 + punctate aggregates (not just diffuse p62 signal), specifically in EC layer II. Hypothalamic p62 analysis involved quantification of the percentage of neurons exhibiting p62 positivity (upregulation or puncta), as well as total NeuN + neurons (images were binarized and number of neurons quantified in ImageJ with the “Analyze Particles” function), per subregion (LH, mPOA, LPO). LC p62 analysis involved quantification of neurons that were PHF1 + and p62 + (upregulation or puncta). These analyses (1 section, both hemispheres, per region per mouse) were normalized to regional area or total neurons, when appropriate, as indicated in Y axes. LAMP1 +/p62 + and LAMP1-/p62 + inclusions were quantified in the LH (1 section, both hemispheres, per mouse) and expressed as a percentage of LAMP1 co-localization. LC LAMP1 images (1 section, both hemispheres, per mouse) were binarized and analyzed for staining density in ImageJ, as a percent area covered. For Fig. 9, analysis of hippocampal p62 was conducted as described above with different sampling: 3 sections spaced 1 in 14, both hemispheres, per mouse. For Fig. 9 hypothalamic p62 analysis, p62 + inclusions were quantified and normalized to region area.

### Statistics

**Fig. 2:**
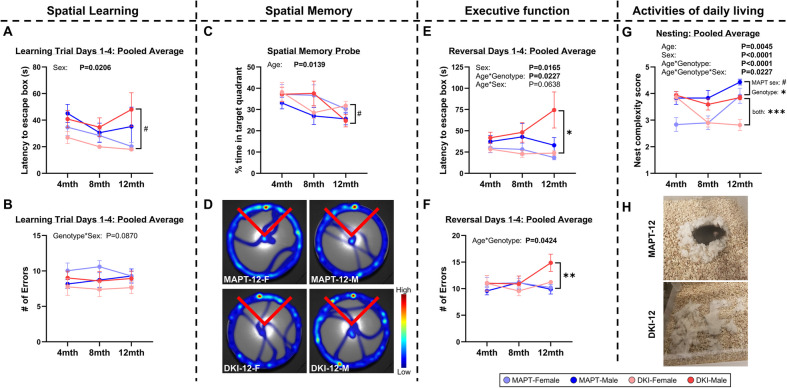
Cognitive decline onset at the late-stage in *App*^NL−G−F^x*MAPT* mice; greater deficits in males. *App*^NL−G−F^x*MAPT* (DKI) and *MAPT* mice underwent longitudinal behavioural testing in the Barnes maze (spatial learning, memory and executive function) and activities of daily living by nest building, at 4-, 8- and 12-months of age. **A**,**B** 12-month male mice exhibit significantly slower latency to the escape box during learning trials, more so in the male *App*^NL−G−F^x*MAPT*s (trending compared to female *App*^NL−G−F^x*MAPT*s), with no significant differences detected in errors made. **C** Spatial memory performance decreased over age in all mice. Notably there was a trend to less time spent searching the target quadrant in male vs. female *App*^NL−G−F^x*MAPT*. **D** Representative Heatmaps for 12-month male and female *MAPT* and *App*^NL−G−F^x*MAPT* mice demonstrate less time in the target quadrant, more centre crossings and searches in non-target regions in 12-month male *App*^NL−G−F^x*MAPT* mice; search strategy complexity was significantly impaired in 12-month male *App*^NL−G−F^x*MAPT* mice (see Supplementary Fig. 3). **E**, **F** Impairments in latency to escape and number of errors made in reversal learning trials indicate the significant executive dysfunction in 12-month male *App*^NL−G−F^x*MAPT* mice. **G**,**H** Nesting data highlights a significant deficit in all 12-month *App*^NL−G−F^x*MAPT* mice, which was more advanced in the females. See Supplementary Figs. 4 and 5 for trial-by-trial Barnes maze graphs and hour-by-hour nesting graphs at each age. Data are presented as mean ± SEM; *n* = 10–11/sex/genotype/age. #*P* < 0.10, *P < 0.05, **P < 0.01, ***P < 0.001. Statistical analysis was conducted using a three-way repeated measures ANOVA (age, genotype, sex effects; values reported above graphs), and with two-way ANOVA, Holm-Šídák post-hoc in the 12-month data (multiple comparisons indicated in the graphs); see Supplementary Tables 2 and 3 for complete statistics

**Fig. 3:**
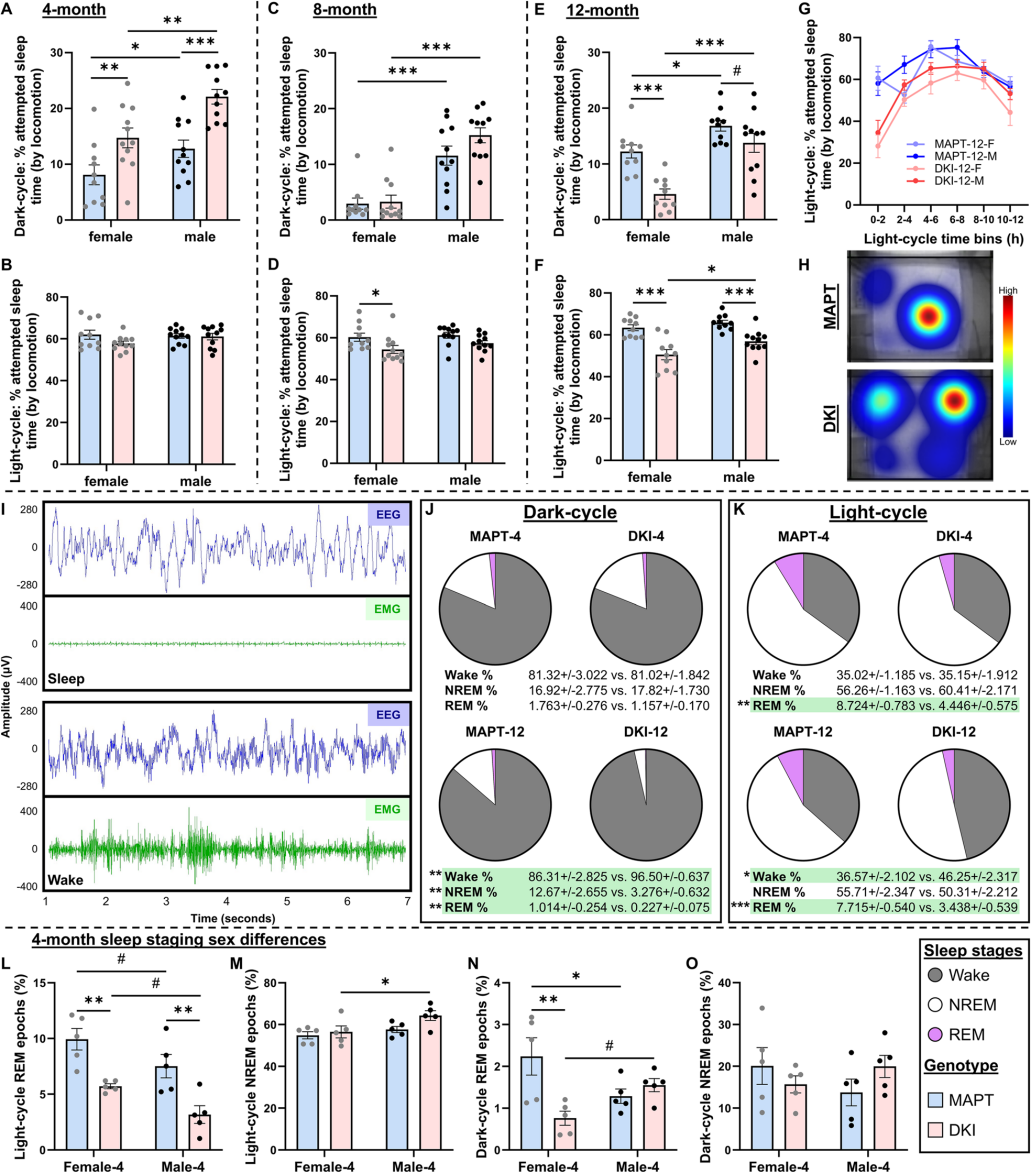
Vulnerability of REM and sleep activity deficits in *App*^NL−G−F^x*MAPT* mice from early-stage pathology; more rapid in female mice. Locomotor activity across 12-h dark- and light-cycles was utilized to measure attempted sleep time. **A** 4-month *App*^NL−G−F^x*MAPT* (DKI) mice had more attempted sleep in the dark-cycle, increased in males of both genotypes. **B** No changes were observed in the 4-month light-cycle. **C** No genotypes differences were detected in the 8-month dark-cycle, with higher attempted sleep time in males than females. **D** An overall significant genotype effect was observed in the 8-month light-cycle, mainly driven by significantly less attempted sleep time in female *App*^NL−G−F^x*MAPT* mice. **E** In the 12-month dark-cycle, *App*^NL−G−F^x*MAPT* mice had less attempted sleep time than *MAPT*s, significant in female *App*^NL−G−F^x*MAPT*s and trending in males; higher in males overall. **F** Both male and female 12-month *App*^NL−G−F^x*MAPT* mice had less attempted sleep time than *MAPT*s, with greater loss in female AD mice. **G** Attempted sleep time separated by 2-h time-bins demonstrate less *App*^NL−G−F^x*MAPT* sleep throughout the light-cycle, with most of the loss in the first half (see Supplementary Fig. 6 for 4- and 8-month binning). **H** Representative light-cycle Heatmaps demonstrate the loss of attempted sleep time in 12-month *App*^NL−G−F^x*MAPT*. **I** Representative EEG and EMG trace demonstrating sleep (top) and wake (bottom) states. **J** EEG/EMG recordings were utilized to stage wake, NREM and REM sleep in 12 h dark-cycle at 4- and 12-months in *App*^NL−G−F^x*MAPT* (DKI-4; DKI-12) and age matched *MAPT* mice (MAPT-4, MAPT-12), demonstrating no overt genotype differences at 4-months, and loss of NREM and REM with increased wake time in 12-month *App*^NL−G−F^x*MAPT* mice. **K** Light-cycle sleep staging indicates a significant loss of REM sleep in 4-month *App*^NL−G−F^x*MAPT* mice, which is also observed at 12-months of age along with increased wakefulness. **L** At 4-months, both male and female *App*^NL−G−F^x*MAPT* mice exhibit a loss of light-cycle REM with a trend to less REM time in general in males. **M** Male 4-month *App*^NL−G−F^x*MAPT* mice spend significantly more of the light-cycle in NREM sleep than female *App*^NL−G−F^x*MAPT*s. **N** When split by sex, 4-month dark-cycle REM sleep has a significant genotype deficit specifically in female *App*^NL−G−F^x*MAPT* mice; male *MAPT*s had less REM time than female *MAPT*s, yet male *App*^NL−G−F^x*MAPT* trended to more REM sleep than female *App*^NL−G−F^x*MAPT*s. **O** No sex or genotype differences were observed in 4-month dark-cycle NREM sleep. No sex differences were observed at 12-months (Supplementary Fig. 7). Data are presented as mean ± SEM; *n* = 10–11/sex/genotype/age (**A**-**G**) *n* = 5/sex/genotype/age (**J**-**O**). #*P* < 0.10, *P < 0.05, **P < 0.01, ***P < 0.001. Statistical analysis was conducted using multiple unpaired t-tests, Holm-Šídák correction (**J**, **K**) or two-way ANOVA, Holm-Šídák post-hoc when appropriate (**A**-**F**, **L**-**O**); see Supplementary Table 2 for complete statistics

**Fig. 4:**
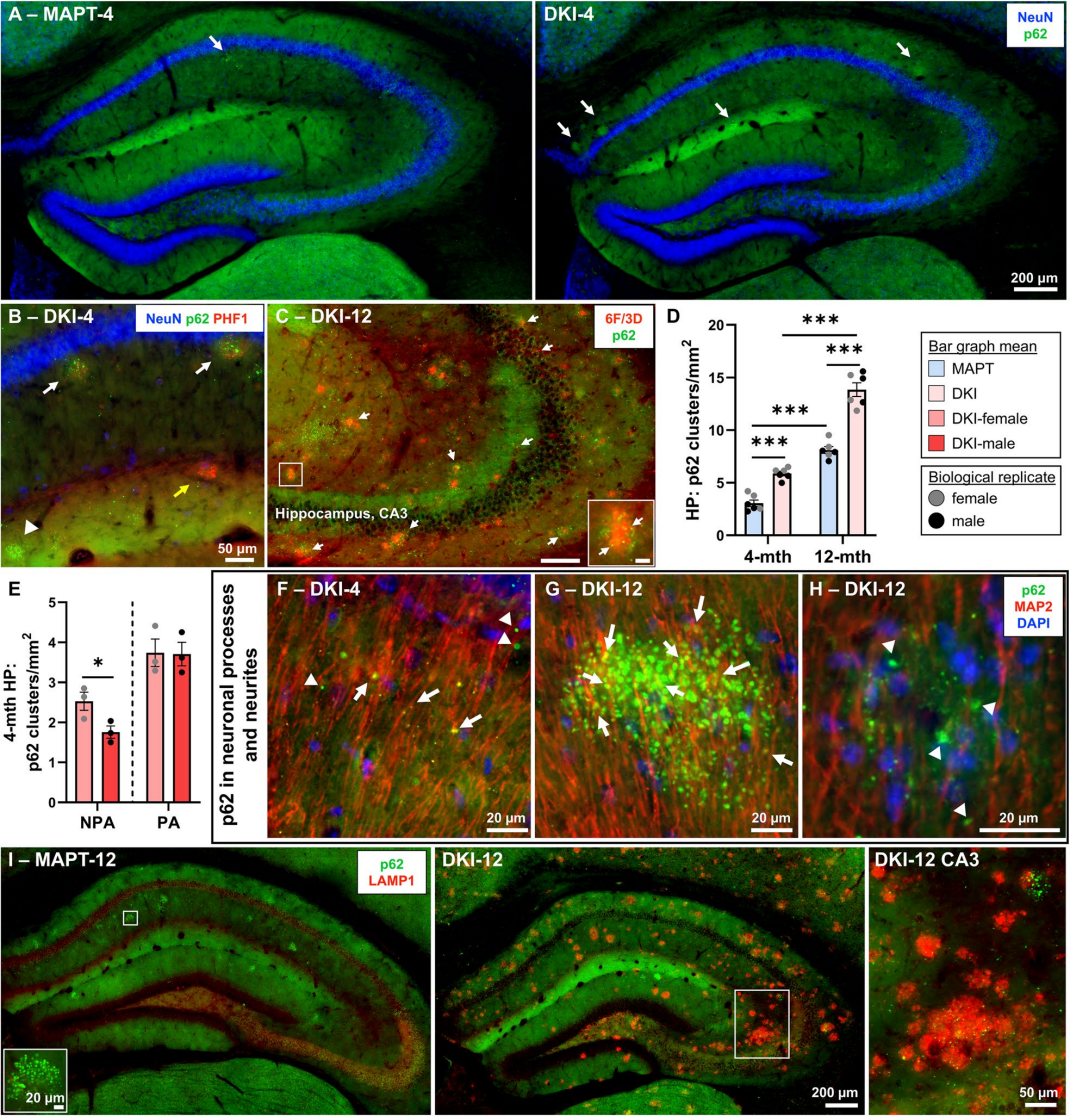
*App*^NL−G−F^x*MAPT* hippocampal autophagic impediment in neuronal processes and dystrophic neurites.** A** p62 (green) and NeuN (blue) staining in 4-month *MAPT* and *App*^NL−G−F^x*MAPT* (DKI) mice demonstrating clustering of uncleared protein (white arrows) in the AD mice; see Supplementary Fig. 11 for 12-month pictures. **B** p62 clusters in close proximity to tau + dystrophic neurites around putative plaques (PHF1, red, white arrows), though this relationship is not exclusive (p62 alone: white arrowhead; PHF1 alone: yellow arrow); see Supplementary Fig. 9 for close-up images of p62/6F/3D staining in 4-month *App*^NL−G−F^x*MAPT* mice. **C** p62 associates with Aβ plaques (6F/3D, red) including co-localization of Aβ in p62 aggregates (scale bars represent 100 µm and 10 µm for the inset); see Supplementary Fig. 10. **D** Hippocampal p62 clusters were quantified determining a significant increase in *App*^NL−G−F^x*MAPT* mice at both early- and late-stage pathology, as well as an age-associated increase in both genotypes. **E** At 4-months of age, female *App*^NL−G−F^x*MAPT* mice have significantly more non-plaque-associated (NPA) p62 clusters than the males, with no changes in plaque-associated (PA) clusters. **F**–**H** p62 aggregates were prominently found in neuronal processes (white arrows) by co-localization with MAP2 (red) and did not frequently appear in proximity to DAPI (blue; see Supplementary Fig. 8) indicating a predominant accumulation of hippocampal p62 in processes > cytoplasm; dystrophic neurites were often p62 +/MAP2- (white arrowheads). **I** LAMP1 (red) staining in *App*^NL−G−F^x*MAPT* and *MAPT* 12-month mice demonstrates a robust lysosomal accumulation surrounding plaques (see Supplementary Fig. 12), and a non-exclusive association of LAMP1 with p62 aggregates in the plaque vicinity, but less so in non-plaque-associated p62 clusters or in the *MAPT*s. Data are presented as mean ± SEM; *n* = 3/sex/genotype/age. *P < 0.05, ***P < 0.001. Statistical analysis was conducted using two-way ANOVA, Holm-Šídák post-hoc (**D**) or unpaired t-tests (**E**); see Supplementary Table 2 for complete statistics

**Fig. 5:**
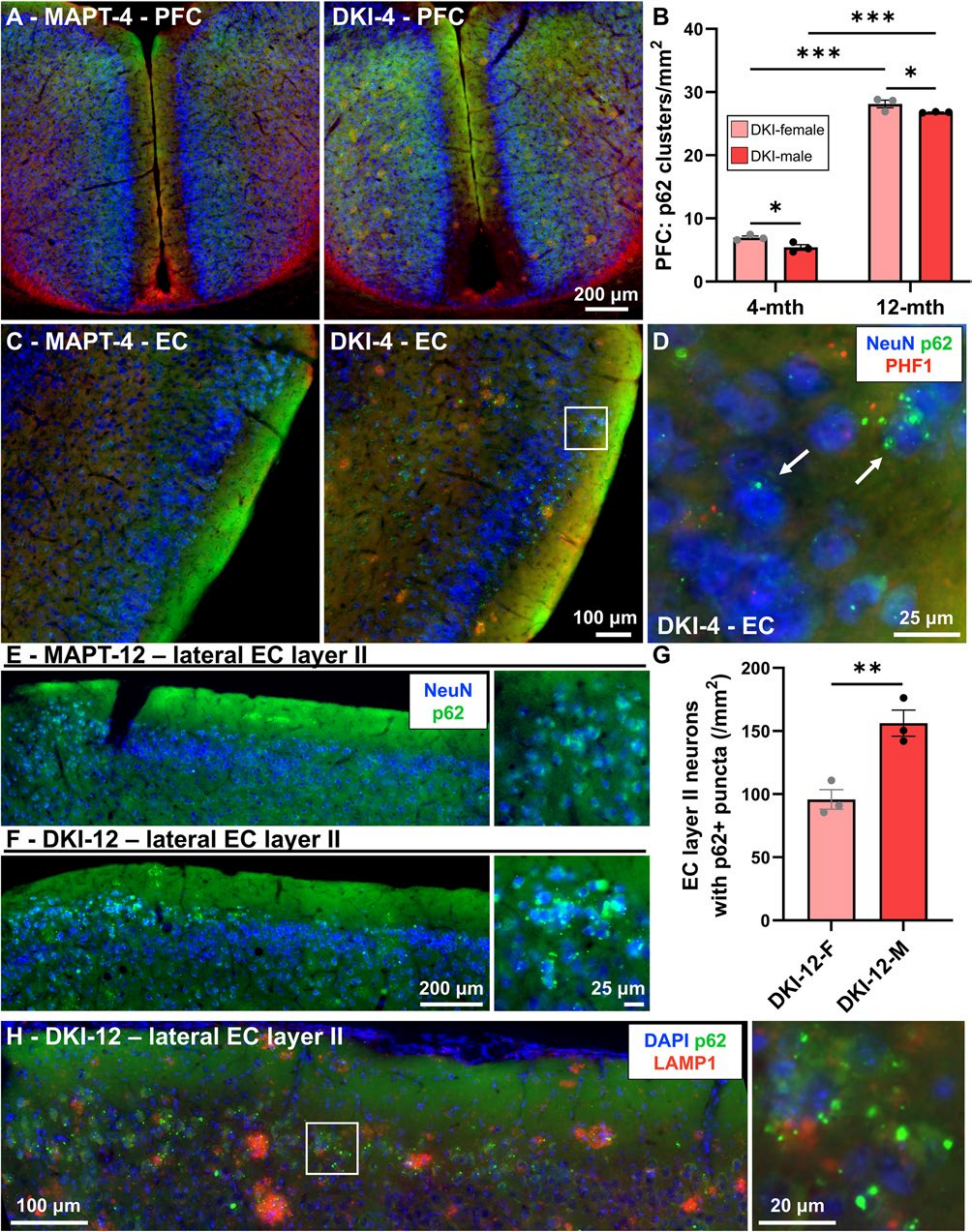
Cortical autophagic impediment follows plaque pathology in *App*^NL−G−F^x*MAPT* mice, except for the vulnerability of entorhinal cortical layer II neurons.** A** Representative p62 (green), NeuN (blue) and PHF1 (red) staining in the 4-month PFC demonstrating a specific association of p62 clusters with putative Aβ plaques in *App*^NL−G−F^x*MAPT* (DKI) mice (surrounding PHF1 positivity), and no clusters in *MAPT*s. **B** p62 clusters were quantified at 4- and 12-months in *App*^NL−G−F^x*MAPT* mice demonstrating significantly more in females, and a large increase with disease progression. **C** p62 follows plaque pathology in the EC as well. **D** EC layer II neurons demonstrate sparse co-localization with p62 + puncta in the 4-month *App*^NL−G−F^x*MAPT*, but not *MAPT*, mice. **E** 12-month *MAPT*s still do not develop p62 + puncta in EC layer II, though they do have p62 immunoreactivity in the cell layer. **F** 12-month *App*^NL−G−F^x*MAPT*s demonstrate robust accumulation of p62 + aggregates within EC layer II neurons. **G** Quantification of p62 +/NeuN + neurons within the EC layer II of month *App*^NL−G−F^x*MAPT* mice, demonstrating a significantly greater burden in male mice. **H** EC layer II puncta in *App*^NL−G−F^x*MAPT* mice predominantly do not co-localize with LAMP1 (red). Data are presented as mean ± SEM; *n* = 3/sex/genotype/age (for analysis and representative images **A**-**G**); *n* = 3/genotype/age (**H**). *P < 0.05, **P < 0.01, ***P < 0.001. Statistical analysis was conducted using two-way ANOVA, Holm-Šídák post-hoc (**B**) or unpaired t-test (**G**); see Supplementary Table 2 for complete statistics

**Fig. 6:**
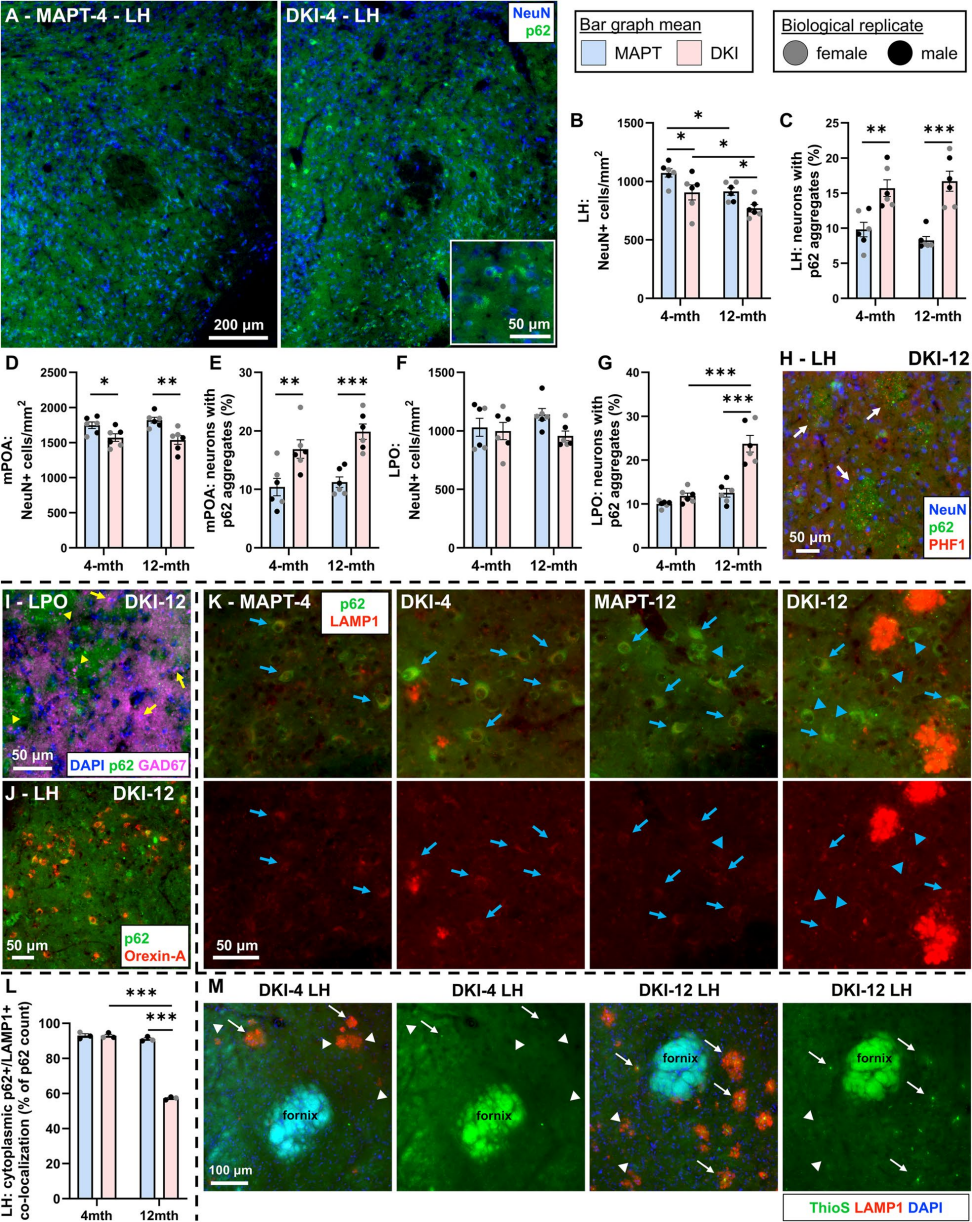
Cytoplasmic autophagic impediments and neuronal injury in the hypothalamus from early-stage *App*^NL−G−F^x*MAPT* progression.** A** Representative images of p62 (green) and NeuN (blue) staining in the lateral hypothalamus (LH), demonstrating greater association of p62 and NeuN in the AD mice. **B** NeuN + neurons were quantified in the LH with a loss in *App*^NL−G−F^x*MAPT* (DKI) mice at both ages, and a significant age effect in both genotypes. **C** 4- and 12-month *App*^NL−G−F^x*MAPT* mice exhibit significantly more LH neurons with p62 immunoreactivity. **D** Neuronal injury was also observed in the medial preoptic area (mPOA) of the *App*^NL−G−F^x*MAPT* hypothalamus, at both ages. **E** mPOA neurons of *App*^NL−G−F^x*MAPT* mice have significantly increased p62 immunoreactivity. **F** No significant differences were detected in the number of NeuN + neurons in the lateral preoptic area (LPO); overall genotype effect was trending to a *App*^NL−G−F^x*MAPT* reduction. **G** p62 +/NeuN + neurons in the LPO were significantly increased in 12-month *App*^NL−G−F^x*MAPT* mice, compared to *MAPT*s and to 4-month *App*^NL−G−F^x*MAPT*s. **H** Hypothalamic p62 clusters were seen associating with plaques (PHF1 positivity, red; white arrows) at 12-months of age, and rarely at 4-months because hypothalamic plaque pathology was sparse at the early-stage (see also results text for plaque counts). **I** LPO GABAergic neurons (GAD67, purple) did not co-localize with p62 at cell bodies (yellow arrows; visualized by co-localization with the blue DAPI signal) or processes (yellow arrowheads). **J** LH orexinergic neurons (Orexin A, red) co-localize with p62 in both ages and genotypes, visualized in the 12-month *App*^NL−G−F^x*MAPT*s. **K**, **L** LH neurons with p62 aggregates exhibit high co-localization with LAMP1 (blue arrows) in 4-month mice of both genotypes and 12-month *MAPT*s. p62 +/LAMP1 + co-localization is significantly less frequent (blue arrowheads) in the 12-month *App*^NL−G−F^x*MAPT*s. **M** Representative Aβ plaque pathology (ThioS, green) in 4- and 12-month *App*^NL−G−F^x*MAPT*s demonstrating low frequency of hypothalamic ThioS + plaques at 4-months. LAMP1 accumulation surrounding plaques precedes the formation of β-sheet structure detected by ThioS (white arrows indicate ThioS +/LAMP1 +; white arrowheads indicate ThioS-/LAMP1 +), further indicative of early-stage disruption in the autophagic-lysosomal system. Data are presented as mean ± SEM; *n* = 3/sex/genotype/age (**B**-**G**) or *n* = 3/genotype/age (**K**-**M**). *P < 0.05, **P < 0.01, ***P < 0.001. Statistical analysis was conducted using a two-way ANOVA, Holm-Šídák post-hoc when appropriate; see Supplementary Table 2 for complete statistics

**Fig. 7:**
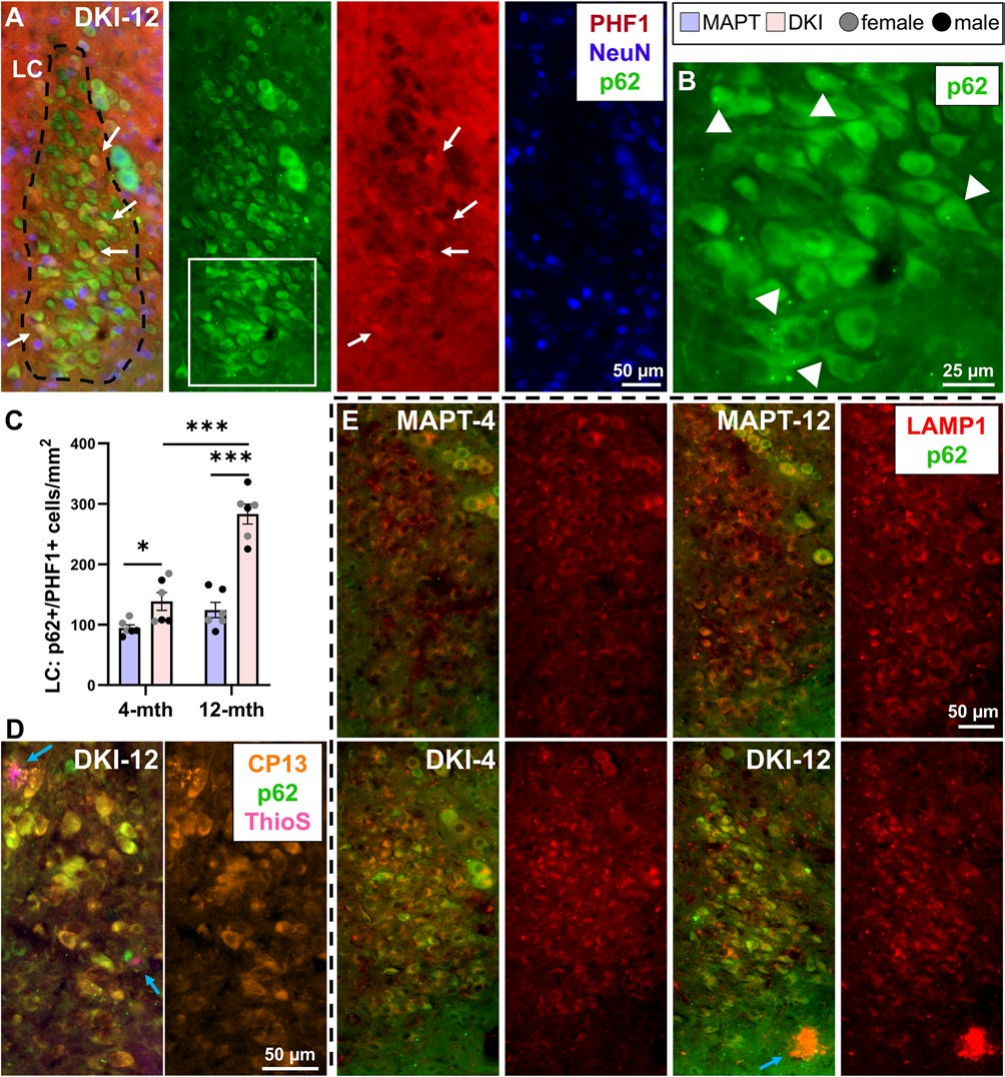
Vulnerability of locus coeruleus neurons to tau pathology and autophagic impediment from early-stage *App*^NL−G−F^x*MAPT* pathology. **A** Representative images of a 12-month *App*^NL−G−F^x*MAPT* (DKI) mice with immunostaining for p62 (green), NeuN (blue) and PHF1 (red) demonstrating the association and abundance of p62 +/PHF1 + neurons (white arrows). **B** LC neurons exhibit high p62 immunoreactivity as well as p62 puncta aggregates (white arrowheads) in *App*^NL−G−F^x*MAPT* mice. **C** Significant increases in LC p62 +/PHF1 + neurons were observed in 4- and 12-month *App*^NL−G−F^x*MAPT* mice, compared to *MAPT*s, with a robust increase over disease in *App*^NL−G−F^x*MAPT*s. **D** LC neurons were predominantly also positive for phosphorylated CP13 (orange) tau (Ser202), including p62 co-localization. ThioS + (pink) plaques (blue arrows) were present, yet rare, at 12-months in close proximity to the LC (see results text for quantification). **E** LC LAMP1 signal (red) is unchanged by genotype or age (see results text for quantification) despite the increase in p62 aggregates in *App*^NL−G−F^x*MAPT* mice. Data are presented as mean ± SEM; *n* = 3/sex/genotype/age (**A**-**C**); *n* = 3/genotype/age (D, E). *P < 0.05, ***P < 0.001. Statistical analysis was conducted using a two-way ANOVA, Holm-Šídák post-hoc; see Supplementary Table 2 for complete statistics

**Fig. 8:**
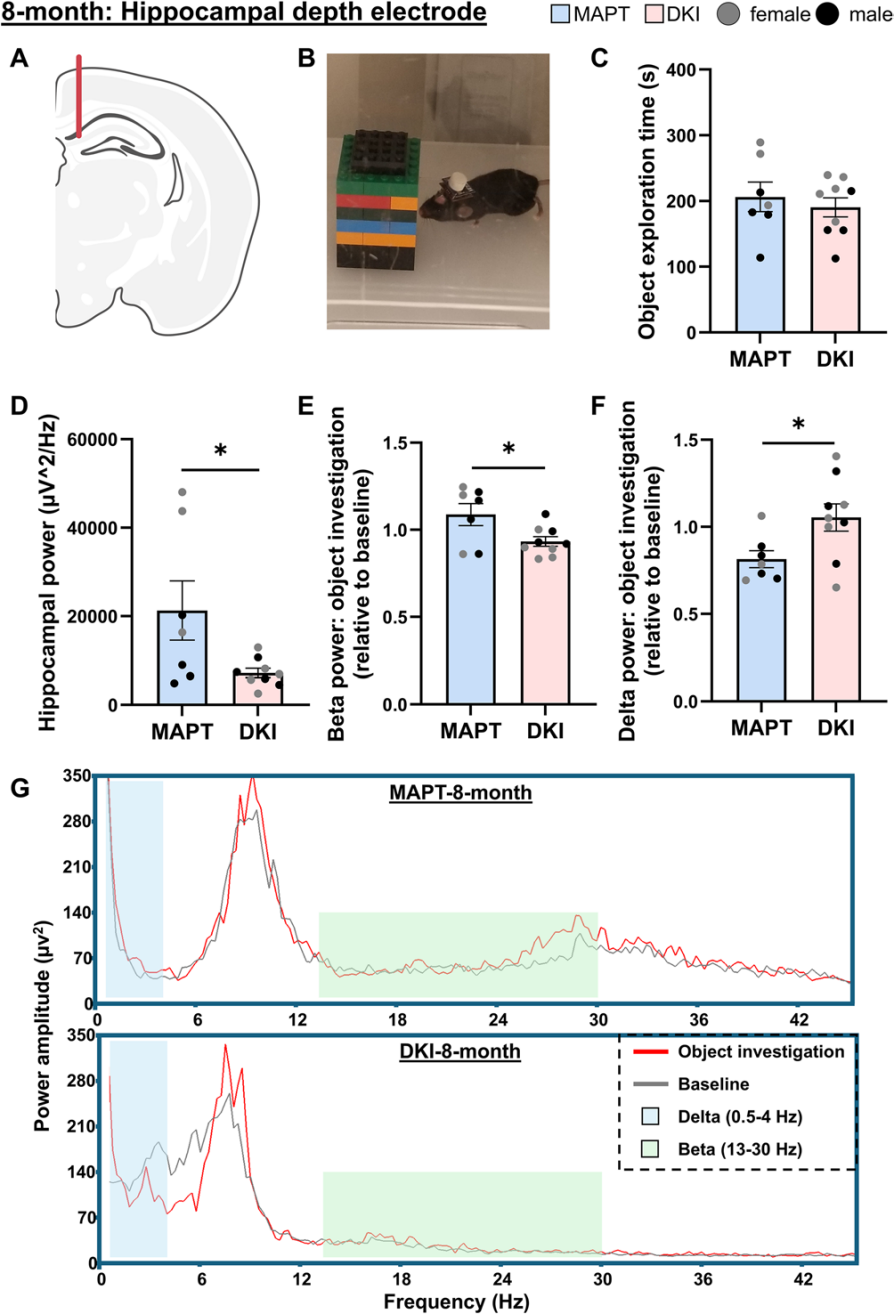
Electrophysiological slowing during cognitive processing as an early sign of cognitive decline in *App*^NL−G−F^x*MAPT* mice. **A** *App*^NL−G−F^x*MAPT* (DKI) and *MAPT* mice at the mid-stage (8-months) were implanted with a hippocampal electrode attached to a headcap, in the CA1 of the hippocampus. **B** Mice were then recorded wirelessly at baseline and during the learning phase of an object recognition task. **C** No differences were detected in time spent exploring/learning the objects; but we do note that female mice explored significantly more than males. **D** *App*^NL−G−F^x*MAPT* exhibit a significant loss of hippocampal power, primarily in females (see results text for sex statistics). **E** Hippocampal beta power was expressed as the ratio of change from baseline to during active object recognition, demonstrating a significant deficit in *App*^NL−G−F^x*MAPT* mice compared to *MAPT*s. **F** Conversely, delta power in *App*^NL−G−F^x*MAPT* mice was significantly higher than *MAPT*s during object learning. No changes were observed in theta or alpha power (see Supplementary Fig. 15). **G** Representative frequency spectra for *MAPT* and *App*^NL−G−F^x*MAPT* mice separated by baseline (gray line) and object investigation (red line) demonstrates greater delta waveforms (blue shading) in *App*^NL−G−F^x*MAPT* mice, and higher beta waveforms (green shading) in *MAPT*s, including an increase during object investigation. Data are presented as mean ± SEM; *n* = 7–9/genotype. *P < 0.05. Statistical analysis was conducted using an unpaired t-test; see Supplementary Table 2 for complete statistics

**Fig. 9:**
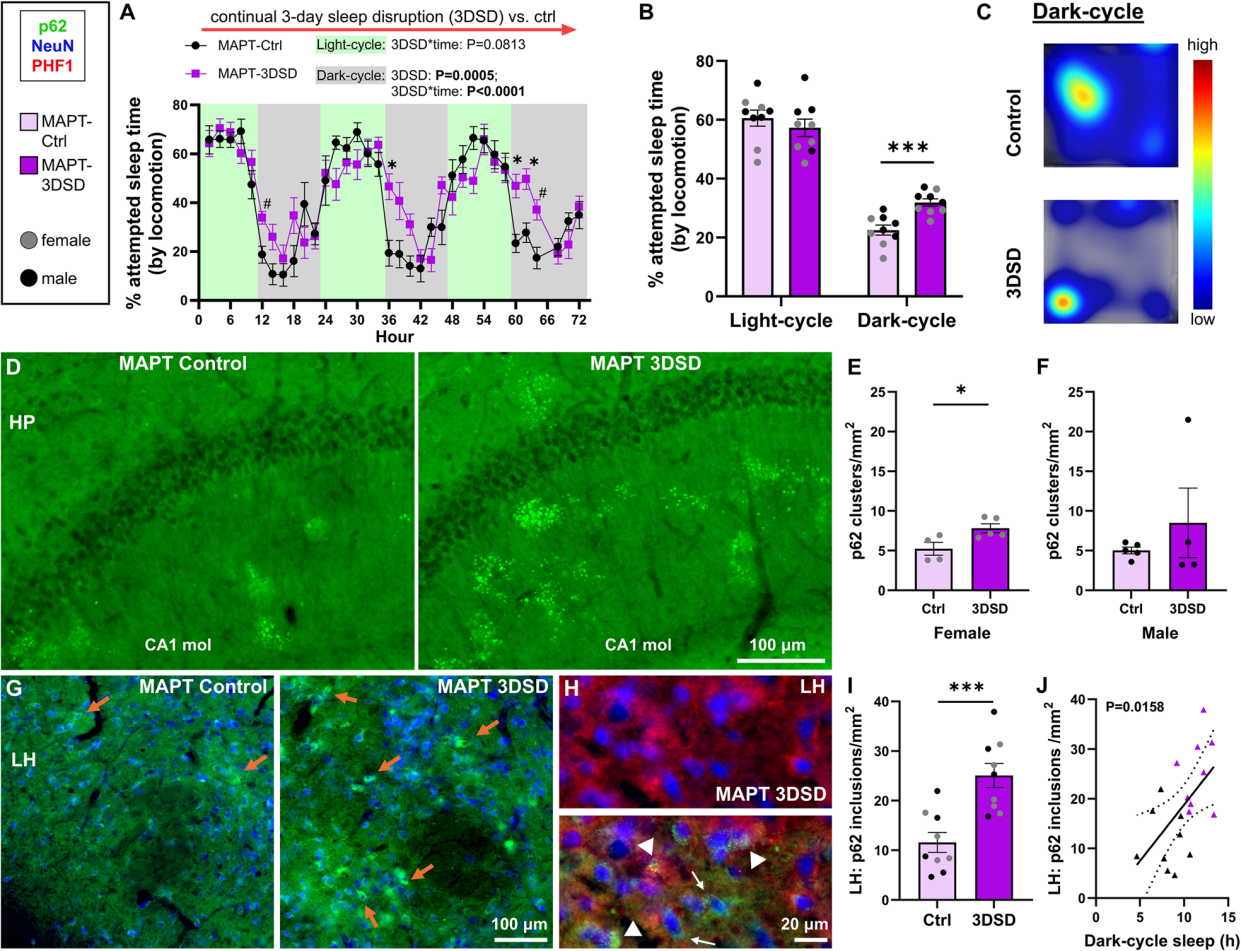
Acute sleep disruption impedes autophagy in the hippocampus and hypothalamus of *MAPT* control mice. *MAPT* mice at 10–12-months of age underwent a 3-day sleep disruption (3DSD) compared to control conditions (Ctrl). **A**,**B** Locomotor activity was measured during the 3-day sleep disruption period demonstrating circadian arrhythmicity in attempted sleep time, and significantly more dark-cycle attempted sleep time, in 3DSD *MAPT* mice. **C** Representative Heatmaps of dark-cycle locomotor activity demonstrate greater inactivity and attempted sleep time in 3DSD *MAPT* mice. **D** p62 (green) clusters increase in the hippocampus of 3DSD *MAPT* mice. **E**,**F** p62 clusters of aggregates were quantified in the hippocampus demonstrating a significant increase in 3DSD vs. Ctrl, primarily in the female mice. **G** Hypothalamic neurons (NeuN, blue) exhibit increased cytoplasmic p62 + inclusions (orange arrows) after sleep disruption. **H** p62 associates with p-tau (PHF1, red, white arrowheads), though not exclusively (white arrows for p62 +/NeuN +/PHF1-). **I** Hypothalamic p62 inclusions are significantly increased in 3DSD *MAPT* mice. **J** Hypothalamic p62 inclusions significantly correlate with the attempted sleep time in the dark-cycle (black triangles = Ctrl; purple triangles = 3DSD). Data are presented as mean ± SEM; *n* = 4–5/sex/condition. *P < 0.05, ***P < 0.001. Statistical analysis was conducted using a two-way ANOVA (**A**), multiple unpaired t-tests, Holm-Šídák correction (**B**), unpaired t-test (**E**,**F**,**I**), or linear regression (**J**); see Supplementary Table 2 for complete statistics

**Fig. 10:**
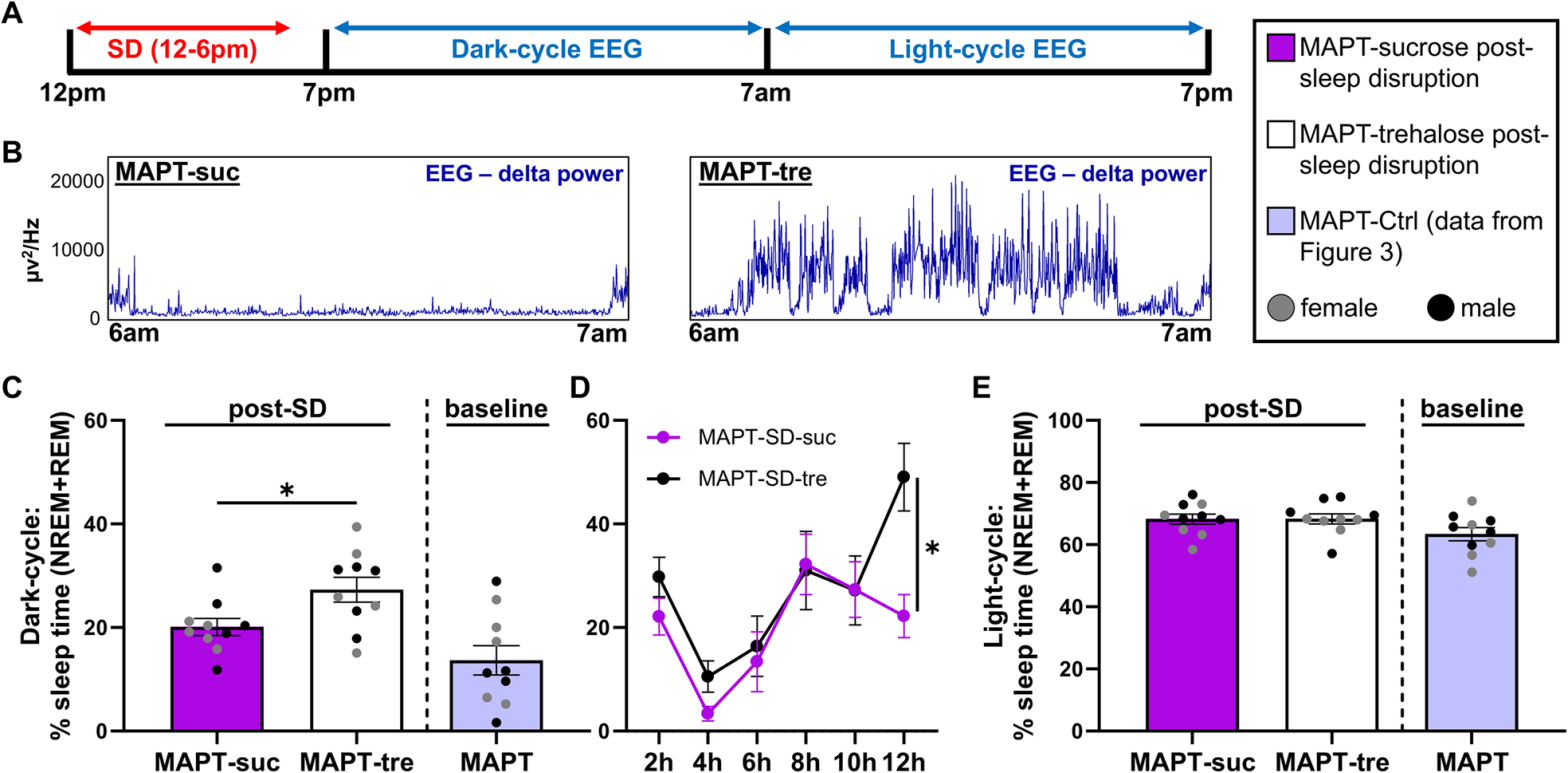
Activating autophagy with trehalose improves sleep recovery. **A** *MAPT* mice at 12-months of age were continuously treated with 2% trehalose (MAPT-tre), or 2% sucrose control (MAPT-suc), and underwent a 6-h sleep disruption (SD). The mice were then recorded with EEG for sleep staging in the immediate dark-cycle then light-cycle, in the 24 h following SD. SD reduced sleep time by ~ 60% in both treatment groups (see Supplementary Fig. 16). **B** An hour before the onset of the first light-cycle following SD, trehalose treated mice exhibit increased sleep-associated EEG delta power than in the control treatment. **C**,**D** Wake, NREM and REM sleep was staged during the dark-cycle immediately post-SD determining greater sleep time (NREM + REM) in MAPT-tre mice, primarily in the 2-h leading up to the sleep-dominant light-cycle. **E** No differences by treatment were detected in the light-cycle. For comparison, baseline *MAPT* datapoints were re-graphed from the 12-month *MAPT*s in Fig. 3. Data are presented as mean ± SEM; *n* = 5/sex/condition. *P < 0.05. Statistical analysis was conducted using an unpaired t-test (**C**,**E**) or two-way ANOVA, Holm-Šídák post-hoc (**D**); see Supplementary Table 2 for complete statistics

GraphPad Prism 10 was utilized for the generation of graphs and statistical analyses. When appropriate, two-sided statistical tests included t-tests, one-, two- or three-way ANOVAs (with or without repeated measures), linear regressions, and correlations. In cases with multiple comparisons (multiple t-tests, ANOVA post-hoc), the Holm-Šídák correction was utilized. Statistics (F, dF, P, r^2^, regression equation) and the utilized test are reported in Supplementary Tables 2 and 3 or in the results text. All biological replicates were mice. Data are expressed as mean ± SEM in the figures or results text. Two mice died in the longitudinal cognitive and locomotor activity cohort between the 8- and 12-month timepoints. One mouse was excluded from the hippocampal depth electrode experiment due to noisy signal from improper implantation. An additional 14 mice (8 males, 6 females) were utilized for optimization of depth electrode surgeries and for EEG longitudinal assessments, which were not included in final analyses. Supplementary Table 2 complements discussion of graphed data in Figs. 1, 2, 3, 4, 5, 6, 7, 8, 9, and 10 in the results section, with the statistical test, and F, t, dF and *P* values.

## Results

### Pathological characterization of *App*^NL−G−F^x*MAPT* mice

*App*^NL−G−F^x*MAPT* mice were assessed at 12-months for Aβ plaques, hyperphosphorylated tau pathology and hippocampal neurodegeneration for basal pathological levels (Fig. 1). Immunostaining for Aβ plaques (6F/3D) revealed progressive deposition over age in *App*^NL−G−F^x*MAPT*s, in line with reported cortical plaque onset between 2-to-4-months of age [17, 19] (Fig. 1A; Supplementary Fig. 1). Diffuse plaques are present in the 4-month hippocampus. This pathology increases significantly at 8- and then 12-months of age, with the presence of cored plaques in CA1, CA3, hilus and dentate gyrus (DG). Notably at 12-months of age there is greater frequency of small, new plaque formations, indicative of a continual and robust Aβ pathology (Fig. 1A; Supplementary Fig. 1 representative images from *n* = 4/age in *App*^NL−G−F^x*MAPT*s). Late-stage *App*^NL−G−F^x*MAPT* exhibit stunted and tortuous neuronal processes in hippocampal molecular layers, especially around putative Aβ plaques (Fig. 1B,C; *n* = 2/sex/age/genotype). At each age we assessed, plaques associated with PHF1 + dystrophic neurites. Non-plaque associated cellular PHF1 inclusions were also abundant (Fig. 1D-F; Supplementary Fig. 1 for 4- and 8-month images, representative images from *n* = 3/age). We conducted a Full pathological assessment at 12-months. Thioflavin-S quantification demonstrated significant Aβ pathology in the neocortex, entorhinal cortex (EC) and hippocampus, with more plaque coverage in the cortical regions compared to hippocampus (*P* =* 0.0006* and *P* = *<* *0.0001*, respectively; Fig. 1G; see Supplementary Table 2 for full statistical parameters). EC plaque coverage was more dense than in the neocortex (*P* = *0.0465*). The 12-month hippocampus has significantly more new plaque formations (10–100 µm^2^; *P* = *0.0066*) and less larger plaques (> 300 µm^2^; *P* = *0.0069*) than the cortex (Supplementary Fig. 1).

Hippocampal neuronal injury onset was observed in the 12-month cohort, with notable thinning in pyramidal layers compared to *MAPT* controls (Fig. 1D,H; Supplementary Fig. 1 for 4- and 8-month images). Quantification of NeuN + hippocampal neurons determined a significant loss of ~ 7.2% of NeuN signal in 12-month *App*^NL−G−F^x*MAPT* mice, compared to age-matched *MAPT* mice (*P* = *0.0001*) primarily from less excitatory pyramidal NeuN in the CA3 (*P* = *0.0263*) and CA1 (*P* = *0.0339*). Granular DG NeuN was less in *App*^NL−G−F^x*MAPT* mice, but non-significant (*P* = *0.1127*). Significantly more non-cell layer NeuN (*P* = *0.0439*) were detected in *App*^NL−G−F^x*MAPT* compared to *MAPT* mice, potentially due to ectopic neurons as PHF1 + cellular inclusions were often localized to molecular layers (Fig. 1D,F), or GABAergic compensation [37]. We did not observe association of cleaved caspase-3 (CCP3) with NeuN in any assessed region (Supplementary Fig. 2): NeuN loss therefore indicates neuronal injury and degeneration [37, 38] but not widespread neuronal apoptosis. These data highlight the vulnerability of excitatory neurons (Fig. 1H) and neuronal processes (Fig. 1B,C) within the hippocampus of 12-month-old *App*^NL−G−F^x*MAPT* mice.

Cellular (non-plaque associated) and neuritic (plaque-associated) tau inclusions were quantified by PHF1 positivity in the hippocampus of 12-month *App*^NL−G−F^x*MAPT* mice. Compared to *MAPT*s, *App*^NL−G−F^x*MAPT* mice exhibited a significant > 2 × increase in p-tau + cells in total hippocampus (*P* < 0.0001; Fig. 1I). PHF1 tau phosphorylation was observed in *MAPT* mice in hippocampal processes and in the cytoplasm (Fig. 1D), yet was notably increased in *App*^NL−G−F^x*MAPT*s, in line with previous characterization of these models [16]. PHF1 + dystrophic neurites were quantified in *App*^NL−G−F^x*MAPT* mice and assessed for sex effects. Male *App*^NL−G−F^x*MAPT* mice exhibit a trend to more neuritic tau pathology overall than females (*P* = 0.0810; Fig. 1J), a significantly greater ratio of neuritic:cellular PHF1 inclusions (*P* = 0.0224; Fig. 1K), and a trend to more PHF1 + neurites per plaque (*P* = 0.0601; Fig. 1L).

### *App*^NL−G−F^x*MAPT* cognitive decline aligns with late-stage hippocampal pathology

Next we assessed cognition at early-, mid- and late-stage *App*^NL−G−F^x*MAPT* pathology using the Barnes maze to identify 3 cognitive domains: spatial learning, memory and executive function (Fig. 2). Mice were trained for the location of an escape box, underwent learning trials, tested for memory recall, then tested for executive function to find the new escape location in reversal learning trials. No overt age or genotype effects were detected in the learning trials (latency: Fig. 2A; errors: Fig. 2B), indicating that *App*^NL−G−F^x*MAPT* mice effectively learn the Barnes maze task until the late-stage. Male *App*^NL−G−F^x*MAPT* mice at 12-months trended to slower performance in learning trials, but not greater errors, compared to female *App*^NL−G−F^x*MAPT*s. In the memory probe, a significant deficit over age was observed: 12-month males spent less time in the target quadrant, trending less in male *App*^NL−G−F^x*MAPT* compared to female *App*^NL−G−F^x*MAPT* (*P* = 0.0833), but unchanged in the *MAPT* sex comparison (Fig. 2C). Late-stage male *App*^NL−G−F^x*MAPT* mice rely on non-direct (random, serial) search strategies compared to all other age-matched groups (Supplementary Fig. 3). Representative 12-month heatmaps demonstrate more centre crossings and searches in non-target quadrants in male *App*^NL−G−F^x*MAPT* mice (Fig. 2D). Executive function in reversal trials significantly declined over age and genotype (latency: Fig. 2E; errors: Fig. 2F), highlighting a cognitive deficit in 12-month male *App*^NL−G−F^x*MAPT* mice; female *App*^NL−G−F^x*MAPT* mice also made more errors over subsequent trial days (Supplementary Fig. 4I). We assessed nest building at each age as an activity of daily living readout (ADL). This data highlights significantly impaired ADLs in 12-month *App*^NL−G−F^x*MAPT* mice compared to *MAPT*s, with a greater deficit in female *App*^NL−G−F^x*MAPT*s (Fig. 3G,H). Complete statistics for Fig. 2 are in Supplementary Table 2, and graphs at each age are presented in Supplementary Figs. 4 and 5 and statistics in Supplementary Table 3.

### Sleep impairment begins in early-stage *App*^NL−G−F^x*MAPT*s, precedes cognitive decline and is more prominent in females

To assess daily activity and sleep patterns, we recorded *App*^NL−G−F^x*MAPT* and *MAPT* mice in home-cages over a 24-h period (Fig. 3). We utilized locomotor data to predict sleep vs. wake states [27, 31], and binned data by the wake-dominant dark-cycle and sleep-dominant light-cycle (both 12-h). Importantly, this analysis does not delineate true sleep from quiet wakefulness, and we therefore utilize it as a measure of “attempted sleep”. At 4-months, *App*^NL−G−F^x*MAPT* mice exhibit increased time spent in attempted sleep states during the dark-cycle (genotype: *P* < 0.0001), with significantly more sleep time in female (*P* = 0.0065) and male DKIs (*P* = 0.0004; Fig. 3A), compared to MAPTs. A significant sex effect was detected as well, with males having greater attempted sleep in the dark-cycle. There were no differences in 4-month light-cycle attempted sleep time (Fig. 3B), suggesting potential disruptions in sleep quality in early-stage *App*^NL−G−F^x*MAPT* mice leading to attempts at sleep recovery during the dark-cycle. At the 8-month mid-stage, no genotype differences were seen in dark-cycle, yet males generally spent more time attempting sleep during the dark-cycle (*P* < 0.0001; Fig. 3C). In the sleep-dominant light-cycle, a significant loss of attempted sleep-time was observed in 8-month *App*^NL−G−F^x*MAPT* mice (*P = 0.0054*), primarily in female mice (*P* = 0.0349), though this trended down in males as well (*P* = 0.1022; Fig. 3D). No sex differences were observed in the 8-month light-cycle.

At late-stage pathology, *App*^NL−G−F^x*MAPT* mice had significantly less attempted sleep in the dark-cycle with a large deficit in female *App*^NL−G−F^x*MAPT*s (*P* = 0.0003) and a trend in males (*P* = 0.0886). Male mice attempted sleep significantly more in the dark-cycle than females (*MAPT*: *P* = 0.0144; *App*^NL−G−F^x*MAPT*: *P* < 0.0001; Fig. 3E). *App*^NL−G−F^xMAPTs also spend less time in attempted sleep states during the light-cycle at 12-months (*P* < 0.0001; females: *P* < 0.0001, males: *P* = 0.0007), with even less in female vs. male *App*^NL−G−F^x*MAPT* (*P* = 0.0230), but no sex difference in *MAPT*s (Fig. 3F). To Further delineate activity changes during the light-cycle, we assessed 2-h bins. This elucidated a loss of attempted sleep time in 12-month *App*^NL−G−F^x*MAPT*s primarily within the first 6-h of the light-cycle, indicating potential impairments in adjusting to the environmental cue (light change), and delayed sleep onset (Fig. 3G). These changes were not present at 4-months, and subtle at 8-months (Supplementary Fig. 6). Representative light-cycle heatmap (Fig. 3H) images demonstrate the severity of sleep activity changes in 12-month *App*^NL−G−F^x*MAPT*s relative to *MAPT* mice.

To confirm sleep changes and stage REM and non-REM (NREM) sleep in *App*^NL−G−F^x*MAPT*s, 24-h EEG/EMG recordings were conducted at the early- and late-stage. Representative raw traces demonstrate EEG and EMG activity during sleep and wake states (Fig. 3I). In the 4-month dark-cycle, *MAPT* and *App*^NL−G−F^x*MAPT* mice spend ~ 18–19% of the time asleep and no genotype differences were detected for wake, NREM or REM stages (Fig. 3J). This contrasts with increased attempted dark-cycle sleep-time by activity observed in 4-month *App*^NL−G−F^x*MAPT* mice (Fig. 3A), suggesting higher quiet wakefulness but not more true sleep. At 12-months, dark-cycle NREM and REM sleep time is lower by age, and significantly less in *App*^NL−G−F^x*MAPT* mice compared to *MAPT*s (NREM: *P* = 0.0074; REM: *P* = 0.0081), with more wake time (*P* = 0.0074; Fig. 3J), consistent with sleep activity observations (Fig. 3E). It has been reported in Tg2576 AD mice that dark-cycle sleep is reduced at both 6- and 11-months [39], contradictions which are likely due to differences in disease staging and/or an overexpression (Tg2576) compared to physiological expression (*App*^NL−G−F^x*MAPT*) of pathological species. In the 4-month light-cycle, REM sleep is ~ 50% less in the *App*^NL−G−F^x*MAPT* mice (*P* = 0.0010), with no changes in wake or NREM. At 12-months *App*^NL−G−F^x*MAPT* mice exhibit a ~ 50% reduction in REM (*P* < 0.0001), significantly more wake time (*P* = 0.0125), and a trend to less NREM sleep (*P* = 0.1113; Fig. 3K). These data demonstrate the sensitivity of REM sleep to AD pathology from early stages, consistent with previous reports in the *App*^NL−G−F^ genotype [40, 41], as well as changes in attempted sleep patterns and increased wakefulness over disease progression.

We detected significant sex differences in how male and female *App*^NL−G−F^x*MAPT* mice respond to impaired sleep quality at the early-stage pathology. A significant effect of genotype (*P* < 0.0001) and sex (*P* = 0.0083) was detected on 4-month light-cycle REM sleep, with REM deficits in both sexes of *App*^NL−G−F^x*MAPT* mice (both *P* = 0.0036) and generally less REM sleep in males (both *P* = 0.0861; Fig. 3L). Interestingly, a significant effect of sex was detected in light-cycle NREM time (*P* = 0.0261; trending effect of genotype: *P* = 0.0731) with more NREM sleep in *App*^NL−G−F^x*MAPT* males compared to females (*P* = 0.0421), and unchanged in *MAPT*s (Fig. 3M). In dark-cycle REM sleep, significant genotype (*P* = 0.0362) and genotype*sex effects (*P* = 0.0047) were observed, with less REM sleep time in *App*^NL−G−F^x*MAPT* females compared to *MAPT* females (*P* = 0.0023), no genotype differences in male mice, more REM sleep in *MAPT* females compared to males (*P* = 0.0434), and an increase in *App*^NL−G−F^x*MAPT* males vs. females (*P* = 0.0511; Fig. 3N). These data demonstrate that males at early-stage AD pathology compensate to a loss of “night-time” REM sleep with more NREM sleep and more “day-time” REM sleep whereas female mice do not. Early-stage dark-cycle NREM sleep was not changed by sex or genotype, and trending in genotype*sex interaction (*P* = 0.1147; Fig. 3O). No sex differences in sleep staging were detected at 12-months (Supplementary Fig. 7).

### Failed autophagic flux in memory-regulating regions linked to plaque progression

Immunostaining for p62 was conducted to investigate temporal, regional and neuronal vulnerabilities of *App*^NL−G−F^x*MAPT* mice to autophagic impediment and accumulation of uncleared protein, in association to the behavioral phenotype. p62 is a multifunctional protein involved in trafficking of protein for degradation to the proteasome and through autophagosome-lysosomal-mediated clearance. It is widely utilized as a marker of autophagic flux, with overabundant p62 levels indicative of uncleared protein and a disruption in cellular proteostasis [9, 42]. In the hippocampus, prefrontal cortex (PFC) and EC, p62 distribution was assessed along with NeuN and PHF1 phosphorylated tau in 4- and 12-month-old *App*^NL−G−F^x*MAPT* and *MAPT* mice (Figs. 4 and 5). Representative hippocampal images from 4-month mice demonstrate accumulation of p62 within hippocampal molecular layers, and in processes surrounding the pyramidal cell layers, with a greater accumulation in *App*^NL−G−F^x*MAPT* mice (Fig. 4A). Hippocampal p62 primarily accumulates within neurites and processes as it is less common in cell bodies in the region (Supplementary Fig. 8; representative images from *n* = 3/sex/genotype/age). p62 + neurites present commonly in clusters in both the *App*^NL−G−F^x*MAPT* and *MAPT* mice (Fig. 4A, white arrows). These clusters are often associated with Aβ plaques in *App*^NL−G−F^x*MAPT* (visualized by surrounding PHF1 positivity and by Aβ staining), but are not exclusively around plaques, nor do all hippocampal plaques have p62 accumulations (Fig. 4B; Supplementary Fig. 9). p62 aggregates are frequently Aβ + (6F/3D residue 8–17) (Fig. 4C, inset, and Supplementary Fig. 10 for hypothalamus co-localization; *n* = 4/age), but are more often Aβ-. We quantified hippocampal p62 clusters (> 10 p62 + neurites) at 4- and 12-months and determined significantly greater p62 accumulation in *App*^NL−G−F^x*MAPT* compared to *MAPT* mice at both ages, and increases over age in both genotypes (all *P* < 0.0001; Fig. 4D). At 12-months, p62 clusters in *MAPT*s were denser and more concentrated yet less frequent, whereas in *App*^NL−G−F^x*MAPT* mice p62 aggregates and clusters were significantly more numerous and spread throughout the hippocampus and cortex (Supplementary Fig. 11; representative images from *n* = 3/sex/genotype).

Within *App*^NL−G−F^x*MAPT* mice, p62 clusters were defined as plaque-associated (PA) and non-plaque-associated (NPA). Female *App*^NL−G−F^x*MAPT* mice at 4-months exhibit significantly more hippocampal non-plaque-associated p62 clusters than male *App*^NL−G−F^x*MAPT* mice (*P* = 0.0485), yet no change in plaque-associated p62 clusters by sex (Fig. 4E). At 12-months, plaque-associated (unpaired t-test, *n* = 3/sex, mean ± SEM; female: 10.03 ± 0.5206, male: 11.72 ± 0.7280; t = 1.886(df = 4), *P* = 0.1324) and non-plaque-associated (female: 3.287 ± 0.7702, male: 2.666 ± 0.3350; t = 0.7391(df = 4), *P* = 0.5008) p62 clusters (per mm^2^ hippocampal area) did not differ by sex. Localization of p62 aggregates to neuronal processes was confirmed with MAP2 staining, indicating deposition of aggregates along axons (white arrows); visualized in CA1 inner molecular layer of a 4-month *App*^NL−G−F^x*MAPT* mouse (Fig. 4F) and consistent in both genotypes and ages (*n* = 2/sex/genotype/age). Representative p62 cluster in the CA1 of a 12-month A*pp*^NL−G−F^x*MAPT* mouse demonstrates prominence of aggregates within MAP2 + processes, and significant non-colocalized aggregates likely in synapses and dystrophic neurites (Fig. 4G). p62 + dystrophic neurites (white arrowheads) around putative Aβ plaques (visualized by contrast) are typically not co-localized with MAP2 (Fig. 4H). Hippocampal p62 clusters in *MAPT*s and non-plaque-associated p62 clusters in *App*^NL−G−F^x*MAPT* mice typically do not co-localize with lysosomal associated membrane protein 1 (LAMP1), indicating impaired lysosomal flux of aggregated protein in these p62 accumulations (Fig. 4I and insets); however, a robust lysosomal accumulation was detected surrounding Aβ plaques including association with p62 (Fig. 4I and insets; Supplementary Fig. 12; *n* = 3/genotype/age).

PFC p62 is almost exclusively associated with neurites and plaques; accumulations were rare in *MAPT*s (Fig. 5A). Plaque-associated p62 clusters were quantified in PFC of *App*^NL−G−F^x*MAPT* mice determining significant sex and age effects, with more p62 in females compared to males at each age (both *P* = 0.0495), and in late-stage vs. early-stage pathology (both *P* < 0.0001; Fig. 5B). EC p62 follows plaque pathology; however, cell body p62 puncta were observed in neurons of *App*^NL−G−F^x*MAPT* mice from the early-stage, mainly in EC layer II (Fig. 5C,D). This increases robustly in the late-stage EC layer II neurons, with multiple p62 + aggregates surrounding NeuN + nuclei in 12-month *App*^NL−G−F^x*MAPT* mice, which is not observed in the age-matched *MAPT*s albeit some p62 immunoreactivity (Fig. 5E,F). EC layer II neurons with p62 + aggregated puncta were quantified in male and female 12-month *App*^NL−G−F^x*MAPT* mice, demonstrating more in males (*P* = 0.0093; Fig. 5G). EC layer II p62 + puncta predominantly did not co-localize with LAMP1 (Fig. 5H). Critically, long-projecting EC layer II-hippocampus circuitry regulates spatial memory [28, 43–45], and higher vulnerability of these neurons to autophagic failure and impaired flux may contribute to sex differences in memory (Fig. 2).

### p62 accumulations from the early-stage in sleep-regulating regions of *App*^NL−G−F^x*MAPT*s, precedes regional plaque pathology; autophagic flux is further impaired with disease progression

Autophagic flux was investigated in the hypothalamus, a critical brain region for sleep–wake regulation (Fig. 6). The lateral hypothalamus (LH) is the major source of orexinergic neurons which activity promotes wake and arousal and is critical for sleep–wake balance, with outputs to other sleep regions including locus coeruleus (LC), basal forebrain and within the hypothalamus [11]. From the early-stage, *App*^NL−G−F^x*MAPT* mice exhibit robust increases in cytoplasmic p62 accumulation in LH neurons (Fig. 6A). Notably, at the 4-month early-stage hypothalamic Aβ plaque pathology is nearly non-existent (4-months: 1.801 ± 0.1220; 8-months: 11.18 ± 0.6287; 12-months: 24.24 ± 0.9590 plaques/mm^2^; *n* = 3–4/age), and therefore these disruptions in autophagic flux precede plaque deposition. Neuronal injury (NeuN loss) and the percentage of neurons exhibiting autophagic impediment (NeuN +/p62 +) were quantified at 4- and 12-months in LH, medial preoptic area (mPOA; less directly sleep–wake-associated) and lateral preoptic area (LPO; sleep-associated inhibitory tone, glutamatergic wake-associated neurons) [11, 46].

*App*^NL−G−F^x*MAPT* mice exhibit significantly less LH NeuN + cells at early- and late-stages, compared to *MAPT*s (4-month: *P* = 0.0252; 12-month: *P* = 0.0293). LH neuronal injury increases with age in both genotypes (*App*^NL−G−F^x*MAPT*: *P* = 0.0380; *MAPT*: *P* **= **0.0331; Fig. 6B). From 4-months, ~ 16% of LH neurons exhibit p62 inclusions in *App*^NL−G−F^x*MAPT*s, significantly greater than *MAPT*s (4-month: *P* = 0.0011; 12-month: *P* < 0.0001), with no effect of age (Fig. 6C). In mPOA, *App*^NL−G−F^x*MAPT* loss of NeuN signal occurred (4-month: *P* = 0.0256; 12-month: *P* = 0.0020; Fig. 6D) and p62 aggregated within neurons (4-month: *P* = 0.0028; 12-month: *P* = 0.0004; Fig. 6E), with no age effects. In LPO, *App*^NL−G−F^x*MAPT* mice trended to a loss of NeuN (*P* = 0.1005; Fig. 6F), yet had a robust increase in neurons impacted by failed autophagic flux in 12-month *App*^NL−G−F^x*MAPT* compared to 4-months, and to *MAPT*s (both *P* < 0.0001; Fig. 6G). These results indicate that the hypothalamus is sensitive to autophagic impediment and neuronal injury prior to significant plaque pathology, and although these changes were not specific to sleep-associated subregions, significant age and genotype*age effects (LH and LPO, respectively; statistics in Supplementary Table 2) suggest a mounting impairment in sleep circuitry.

Neurite and plaque-associated p62 clustering in the hypothalamus was quite rare at 4-months but common at 12-months in *App*^NL−G−F^x*MAPT*s (Fig. 6H; representative images from *n* = 3/sex/age), coinciding with plaque counts. GABAergic (GAD67) and orexinergic (Orexin-A) neuronal co-localization with p62 was assessed to determine which hypothalamic neurons were vulnerable to autophagic impediment. No inhibitory neurons exhibited cytoplasmic or neurite depositions of p62 in the LPO (Fig. 6I; see Supplementary Fig. 13 for PFC; *n* = 2/genotype/age), despite GABAergic dystrophic neurite pathology, indicating an excitatory neuronal vulnerability to autophagic impediment. LH orexinergic neurons exhibit significant p62 inclusions at a similar rate as the NeuN +/p62 + quantification (Fig. 6J; *n* = 2/genotype/age). Representative images for orexinergic results are in 12-month *App*^NL−G−F^x*MAPT* mice, though these results are consistent (to a lesser degree) at 4-months and in *MAPT*s. In sum, hypothalamic sleep–wake regulating neurons are sensitive to autophagic disruption.

Next, we assessed LH cytoplasmic p62 + inclusions for co-localization with LAMP1 as an additional indicator of autophagic flux. Nearly all (~ 90–93%) p62 inclusions were LAMP1 + in *MAPT* mice at both ages and 4-month *App*^NL−G−F^x*MAPT* mice, yet a significant loss (*P* < 0.0001) of co-localization was observed in the 12-month *App*^NL−G−F^x*MAPT* mice (~ 57% co-localization; Fig. 6K,L), indicating a mounting impediment in autophagic flux in sleep-regulating neurons in the AD mice. Representative LAMP1/ThioS/DAPI images in 4- and 12-month *App*^NL−G−F^x*MAPT* mice demonstrate the sparsity of hypothalamic plaques at the early-stage, and that lysosomal accumulation precedes deposition of β-sheet plaques (Fig. 6M; see also Supplementary Fig. 12 for hippocampal and cortical images), further indicating the autophagic-lysosomal burden in the hypothalamus of the AD mice from early disease stages.

The LC is a neuromodulatory system with noradrenergic afferents to hippocampus, hypothalamus and cortex, regulating sleep and memory [11], and one of the first regions to exhibit tau pathology in AD [2]. We immunostained the LC of 4- and 12-month *App*^NL−G−F^x*MAPT* mice with p62, PHF1 and NeuN (Fig. 7). LC neurons are typically NeuN-, which guided regional identification. Representative images from 12-month *App*^NL−G−F^x*MAPT* mice demonstrates accumulation of cytoplasmic p62 within LC neurons, frequency of phosphorylated-tau pathology, and the co-localization of p62 with p-tau (white arrows) albeit a non-exclusive relationship (Fig. 7A). LC neurons exhibit p62 puncta (white arrowheads) further indicating failed autophagic flux and overabundance of uncleared protein (Fig. 7B). We quantified p62 +/PHF1 + LC neurons in *App*^NL−G−F^x*MAPT* and *MAPT* mice. Significantly more p62 +/PHF1 + cells were detected in *App*^NL−G−F^x*MAPT* mice at both ages (4-month: *P* = 0.0266; 12-month: *P* < 0.0001), and in 12-month vs. 4-month *App*^NL−G−F^x*MAPT* (*P *< 0.0001). Age had no effect in *MAPT*s (Fig. 7C). Non-cytoplasmic clusters of p62 aggregates were quite rare in the LC even at 12-months. ThioS + Aβ plaques were assessed in 4- and 12-month *App*^NL−G−F^x*MAPT* LC: demonstrating 0 and 1.125 plaques/LC hemisphere, respectively (*n* = 4 mice/age). LC neurons were also frequently positive for phosphorylated tau at CP13 (Ser202), a more advanced tauopathy marker, especially in 12-month *App*^NL−G−F^x*MAPT* mice and in association with p62 (Fig. 7D). CP13 was also prevalent in dystrophic neurites in the hippocampus and hypothalamus, as well as in hippocampal cellular tau inclusions, and in co-localization with hypothalamic p62 + inclusions (Supplementary Fig. 14). We assessed LC for lysosomal alterations with LAMP1 and observed no changes in LAMP1 signal by age or genotype (LC area covered by LAMP1 (mean ± SEM): 4-month *MAPT* 8.897% ± 1.197; 4-month *App*^NL−G−F^x*MAPT* 9.280% ± 1.205; 12-month *MAPT* 8.867% ± 0.4554; 12-month *App*^NL−G−F^x*MAPT* 7.838 ± 1.181; age*genotype: F(1,8) = 0.4441, *P* = 0.5239; age: F(1,8) = 0.4831, *P* = 0.5067; genotype: F(1,8) = 0.0929, *P* = 0.7683; two-way ANOVA, *n* = 3/age/genotype). Representative LAMP1/p62 images in the LC demonstrate the severity of p62 accumulation in *App*^NL−G−F^x*MAPT* mice, the increase over disease progression, and the impediment of autophagic flux to the lysosome due to an unchanging LAMP1 signal (Fig. 7E). These data indicate the vulnerability of LC neurons to autophagic and tau pathologies from the early-stage and with disease progression. This further demonstrates that mounting autophagic impairments in sleep-circuitry coincide with the progression of sleep deficits from early-through-late-stage *App*^NL−G−F^x*MAPT* pathology.

### Sleep-to-cognition linkage: sleep-associated delta waves are prevalent during cognitive processing in *App*^NL−G−F^x*MAPT* mice prior to cognitive decline

We next assessed hippocampal neuronal electrophysiology during cognitive testing in *App*^NL−G−F^x*MAPT* mice (Fig. 8). At 8-months of age, prior to memory impairments (Fig. 2), *App*^NL−G−F^x*MAPT* and *MAPT* mice were implanted with a hippocampal depth electrode, in the CA1 region of the dorsal hippocampus, attached to a headcap for wireless recording (Fig. 8A). After habituation, mice were placed in a cage with 2 copies of a novel object and electrophysiological recordings were collected (Fig. 8B). No genotype differences were detected in the amount of time mice spent exploring the objects (*P* = 0.5486; Fig. 8C), though females in general explored longer than males (two-way ANOVA, sex: F(1,12) = 11.00, *P* = 0.0062). Total hippocampal power was significantly lower in *App*^NL−G−F^x*MAPT* mice compared to *MAPT*s (*P* = 0.0336; Fig. 8D), primarily in female mice (two-way ANOVA, sex: F(1,12) = 9.669, *P* = 0.0090; genotype: F(1,12) = 14.46, *P* = 0.0025; genotype*sex: F(1,12) = 9.483, *P* = 0.0095). Neuronal frequency was binned for delta (0.5–4 Hz), theta (4–8 Hz), alpha (8–13 Hz) and beta (13–30 Hz) bands and the ratio of change during object investigation compared to baseline was assessed. Notably, hippocampal beta power increased in *MAPT*s but decreased in *App*^NL−G−F^xMAPTs while learning the novel object (*P* = 0.0283; Fig. 8E). Conversely, delta power was lower in *MAPT*s and unchanged in *App*^NL−G−F^x*MAPT* mice during object investigation (*P* = 0.0298; Fig. 8F). No changes were seen in theta or alpha (Supplementary Fig. 15). Representative frequency spectra during object recognition vs. baseline highlight greater delta power and lower beta power at baseline and when acquiring the task in *App*^NL−G−F^x*MAPT* mice, and increased beta power only in *MAPT* mice during object investigation (Fig. 8G). These data demonstrate that prior to deficits in cognitive or exploratory behavior, *App*^NL−G−F^x*MAPT* mice exhibit hippocampal neuronal impairments during learning, related to lower attention and wakefulness (beta), electrophysiological slowing and sleepiness (delta) [11, 27, 47, 48]. Electrophysiological signatures of sleepiness and impaired attention occur during cognitive processing in the AD mice, and further signify the importance of sleep deficits in the prodromal phases of AD.

### Sleep-to-autophagy linkage: acute sleep disruption in *MAPT* mice impedes autophagy and mimics an Alzheimer’s-like phenotype

To confirm the sleep-autophagy linkage we conducted an acute 3-day sleep disruption (3DSD) in *MAPT* mice and assessed p62 in the hippocampus and hypothalamus, compared to control (Ctrl) conditions (Fig. 9). We have validated this SD method by EEG previously [27] and in the present study (Supplementary Fig. 16). Attempted sleep time by locomotor activity was measured during the 3DSD and Ctrl periods, demonstrating a disruption in activity patterns highlighted in the increased attempted sleep time in the dark-cycle of 3DSD mice (Fig. 9A). Attempted sleep time across the three days was unchanged in the light-cycle, and significantly greater in the dark-cycle of 3DSD mice (*P* = 0.0009; Fig. 9B,C). 3DSD mice significantly increased their attempted sleep time in the dark-cycle over the 3-days (linear regression, Y = 0.5227*X + 2.783 (hour), r^2^ = 0.3797, F(1,25) = 15.30, *P* = 0.0006); which was trending but not significant in controls (Y = 0.3480*X + 2.007 (hour), r^2^ = 0.1315, F(1,25) = 3.786, *P* = 0.0630). Notably, higher dark-cycle attempted sleep is indicative of disrupted activity patterns as we showed in early-stage *App*^NL−G−F^x*MAPT* mice (Fig. 3A). 3DSD increased p62 accumulation in the hippocampus, with a greater number of, and denser p62 clusters most notably in CA1 molecular layers (Fig. 9D). Quantification of hippocampal p62 aggregates determined a significant increase in 3DSD compared to Ctrl, specifically in the females (*P* = 0.0305; Fig. 9E); whereas higher variability was observed in the males (*P* = 0.4005; Fig. 9F). Cytoplasmic p62 was increased in LH neurons (Fig. 9G). There was a non-exclusive association between cytoplasmic p62 and PHF1 in the hypothalamus (Fig. 9H), indicating vulnerability of sleep-regulating neurons to autophagic impediment and p-tau after sleep loss, akin to the AD phenotype observed in *App*^NL−G−F^x*MAPT* mice. Hypothalamic inclusions were significantly increased in 3DSD vs. Ctrl mice, in mice of both sexes (*P* = 0.0006; Fig. 9I). A significant positive correlation was detected between hypothalamic p62 aggregates and dark-cycle sleep time in 3DSD and control *MAPT* mice (r^2^ = 0.3127, *P* = 0.0158; Fig. 9J), further indicating the connection between sleep loss and autophagic impediment.

### Autophagy-to-sleep linkage: activating autophagy with trehalose in *MAPT* mice promotes NREM and REM sleep recovery following sleep disruption

To probe the effect of activating autophagy on sleep, we treated 12-month *MAPT* mice with 2% trehalose, or 2% sucrose as a control. Trehalose is a natural disaccharide that induces autophagy via transcription factor EB (TFEB), and has gained interest for neuroprotective and anti-aggregant/protein clearance effects in models of neurodegenerative diseases [33–35, 49]. Autophagy targets include increased p62, which we show is activated yet uncleared after sleep disruption (Fig. 9); however, trehalose also increases expression of genes necessary for downstream degradation of p62-sequestered proteins, including microtubule-associated protein 1 light chain 3B-I (autophagosome formation), its lipidated form for promoting continual autophagic flux, and multiple lysosomal genes [49]. Treated EEG Headcap mice underwent a 6-h sleep disruption (Fig. 10A), utilizing the same sleep disruption method as in Fig. 9, which significantly reduced their NREM (by ~ 59%) and REM (by ~ 78%) sleep time (Supplementary Fig. 16). Subsequent EEG recordings were conducted in the immediate dark-cycle and light-cycle after the sleep disruption, to assess sleep recovery. Representative EEG delta power over time in the last hour of the dark-cycle demonstrates a large increase in slow waves in trehalose-treated *MAPT* mice compared to sucrose controls, indicative of improved sleep recovery (Fig. 10B). We quantified NREM and REM sleep in the dark-cycle after sleep disruption and demonstrate a significant increase in sleep recovery in male and female *MAPT* mice with ongoing autophagy activation (*P* = 0.0229), specifically leading up to the next light-cycle (Fig. 10C,D). No changes were observed in the light-cycle sleep time by treatment (*P* = 0.9758; Fig. 10E).

In combination with Fig. 9 results, these data highlight that in a model without neurodegeneration or widespread proteinopathy, *1)* disrupting sleep increases uncleared, sequestered protein in the hippocampus and in hypothalamic sleep–wake neurons, and that *2)* ongoing activation of autophagic flux improves sleep recovery following a sleep disruption; thereby linking the behavioral and pathological outcomes of the sleep-autophagy positive-feedback-loop. Table 1 presents a summary of results and comparisons of the behavioral and autophagic impairments between early- and late-stage AD mice, related to the sleep-to-cognition, sleep-to-autophagy, and autophagy-to-sleep effects.

**Table 1:** Summary of changes in early- and late-stage *App*^NL−G−F^x*MAPT* pathology, related to sleep-to-cognition, sleep-to-autophagy, and autophagy-to-sleep effects. All changes are in both sexes unless otherwise noted. Multiple arrows (2 or 3) in the late-stage column are utilized to indicate autophagy age effects in late- vs. early-stage *App*^NL−G−F^x*MAPT*s

Analysis	Early-stage pathology (*App*^NL−G−F^x*MAPT vs. MAPT*)	Late-stage pathology (*App*^NL−G−F^x*MAPT vs. MAPT*)	Sleep-to-cognition effect (mid-stage *App*^NL−G−F^x*MAPT vs. MAPT*)	Sleep-to-autophagy effect (*MAPT*−3DSD vs. -Ctrl)	Autophagy-to-sleep effect (*MAPT*-trehalose vs. -sucrose)
Cognition	No genotype changes in Barnes maze Activities of daily living intact (↑ compared to *MAPT*)	↔ spatial learning ↓ spatial memory (*males only*) ↓ executive function (*primarily in males*) ↓ activities of daily living (*greater deficit in females*)	↔ in learning/exploration time of novel objects	N/A	N/A
Sleep	↑ dark-cycle attempted sleep ↔ light-cycle attempted sleep ↓ dark-cycle REM EEG (*females only*) ↓ light-cycle REM EEG	↓ dark-cycle attempted sleep (*females: significant; males: trending*) ↓ light-cycle attempted sleep ↓ REM and NREM EEG ↑ wake EEG	N/A	↑ dark-cycle attempted sleep ↔ light-cycle attempted sleep	SD impaired sleep similarly in both cohorts ↑ NREM and REM EEG recovery in the dark-cycle after sleep disruption ↔ light-cycle NREM and REM EEG after sleep disruption
Hippocampus	↑ non-cytoplasmic and neuritic autophagic aggregates (> *in females*) ↔ neurons	↑↑↑ non-cytoplasmic and neuritic autophagic aggregates ↓ pyramidal NeuN ↑ tau in *males vs. females*	↓ beta wave activity during cognitive processing ↑ delta wave activity during cognitive processing	↑ non-cytoplasmic autophagic aggregates (*females only*)	N/A
Cortex	↑ neuritic autophagic aggregates (PFC: > *in females*)	↑↑↑ neuritic autophagic aggregates (PFC: > *in females*) ↑ EC layer II cytoplasmic autophagic aggregates (> *in males*)	N/A	N/A	N/A
Hypothalamus	↑ cytoplasmic autophagic aggregates – excitatory and orexinergic neurons (*LH and mPOA only*) ↓ NeuN (*LH and mPOA only*) ↔ lysosomal targeting of p62	↑↑ cytoplasmic autophagic aggregates – excitatory and orexinergic neurons (*higher in LPO with age*) ↓ NeuN (*LH and mPOA only*) ↓ lysosomal targeting of p62 ↑ neuritic autophagic aggregates	N/A	↑ cytoplasmic autophagic aggregates	N/A
Locus coeruleus	↑ neurons with co-localized tau and autophagic pathologies ↔ cytoplasmic lysosomal signal despite ↑ p62	↑↑↑ neurons with co-localized tau and autophagic pathologies ↔ cytoplasmic lysosomal signal despite ↑↑↑ p62	N/A	N/A	N/A

*Abbreviations*: *3DSD* 3-day sleep disruption, *Ctrl* control, *EC* entorhinal cortex, *EEG* electroencephalogram, *LH* lateral hypothalamus, *LPO* lateral preoptic area, *mPOA* medial preoptic area, *NeuN* neuronal nuclei, *NREM* non-REM sleep, *PFC* prefrontal cortex, *REM* rapid eye movement sleep, *SD* sleep disruption

## Discussion

In this study, we utilized an AD pathology mouse model with physiological expression patterns of Aβ and tau, *App*^NL−G−F^x*MAPT* mice, to investigate the relationship of sleep loss and autophagic impediment at early disease stages. We concurrently characterized the sleep and cognitive phenotype, demonstrating an earlier sensitivity to REM sleep and hippocampal neuronal impairments, with preservation of memory and executive function until late-stage pathology. Critically, the regional, neuronal and temporal vulnerabilities of failed autophagic flux were linked to the behavioral phenotype, in sleep- and memory-circuitry. In the cortex and hippocampus, aggregated uncleared protein was most abundant in neuronal processes, dendrites and dystrophic neurites – putatively in neuronal afferents to these regions – until the late-stage when hippocampal-projecting EC layer II neurons demonstrated impaired autophagic flux, as seen by the accumulation of protein aggregates and lack of lysosomal fusion. We also identified early pathological changes in the hypothalamus and locus coeruleus, from early-stage AD that preceded regional plaque pathology, with neuronal cell bodies exhibiting abundant autophagic aggregates, primarily in excitatory and neuromodulatory systems that promote wakefulness, arousal and regulate the sleep–wake balance. Critically, autophagic flux of sequestered protein to the lysosome could not fully compensate for an increasing abundance in sleep–wake neurons, especially in the late-stage.

We then probed the sleep-to-cognition, sleep-to-autophagy, and autophagy-to-sleep linkages demonstrating *1)* electrophysiological signatures of sleepiness during cognitive processing preceding cognitive decline in the AD mice; *2)* that an acute sleep disruption in *MAPT* mice lead to failed autophagic flux in the hippocampus and hypothalamus aligned to sleep activity impairments, akin to an early AD phenotype; and, finally, *3)* that activation of autophagy with trehalose in *MAPT* mice improved sleep in the recovery period following a sleep disruption.

### Sex differences in the Alzheimer’s disease behavioral phenotype are linked to autophagic pathology in memory-regulating regions

Our findings indicate a sex difference where cognitive decline at the late-stage was greater in AD male mice than females, yet at early- and mid-stages of pathology sleep impairments were more rapid in female AD mice, notably less REM sleep recovery than males. Contrary to our expectations, we observed sex differences in classically cognition-regulating regions, PFC, EC and hippocampus, and not in the sleep–wake circuitry. Hippocampal and cortical impairments in AD are well documented and associated with memory impairments [1–3]. Autophagic aggregates in the *App*^NL−G−F^x*MAPT* hippocampus were observed in molecular layer neuronal processes (CA1, CA3 > DG) and were often negative for the LAMP1 lysosomal marker, suggesting autophagosome and autophagic vacuolar blockage in neuronal afferents, which may be contributing to electrophysiological impairments during information processing. Conversely, plaque-associated dystrophic neurites were commonly p62 +, often associating with LAMP1 accumulations around plaques, in the hippocampus, cortex and in later stages the hypothalamus, and, to a lesser degree in the locus coeruleus. Female *App*^NL−G−F^x*MAPT*s at the early-stage exhibited an increased hippocampal proteostasis burden, observed by clustering of uncleared protein, compared to males. This increase was only in autophagic aggregates that were not dystrophic neurites (not associated with plaques), indicating a greater burden in females that was putatively from impaired hippocampal inputs.

One possible explanation is that increased activity of wake-active neurons with hippocampal inputs, such as in noradrenergic, orexinergic and cholinergic (wake and REM-active) systems during disrupted sleep and leads to a greater protein burden, which includes production and spread of Aβ and tau pathologies [50–55], and is presenting in the female *App*^NL−G−F^x*MAPT* mice as increased synaptic autophagic aggregates. Delorme et al. recently described increased activity of cholinergic and orexinergic inputs to the hippocampus after sleep deprivation, which increased somatostatin-mediated gating of the hippocampal circuits [56]. Also, noradrenergic neuronal activity drops significantly during sleep and to quiescence during specifically REM sleep [11, 57, 58], and therefore loss of REM sleep and reduced REM recovery in the female AD mice could lead to a greater vulnerability of noradrenergic circuitry to proteinopathy. Furthermore, female *App*^NL−G−F^x*MAPT* mice exhibited higher plaque-associated autophagic clusters than males in the PFC from the early-stage, indicative of more advanced Aβ plaque pathology, supporting conclusions that earlier sleep disturbances in females are linked to more rapid proteinaceous production and deposition.

Another major source of hippocampal long-projecting afferents is lateral EC layer II neurons, which progressively accumulated uncleared protein in *App*^NL−G−F^x*MAPT* mice and with notable p62 pathology in the late-stage pathology. This pathway is well known to contribute to the memory and cognitive domains we detected in the Barnes maze [28, 43–45], and therefore is a strong correlate of the *App*^NL−G−F^x*MAPT* cognitive decline. Notably, EC layer II proteostasis burden was significantly greater in male vs. female *App*^NL−G−F^x*MAPT* mice, consistent with the loss vs. preservation of spatial memory impairments, respectively, and the greater executive function deficits in males. Late-stage male *App*^NL−G−F^x*MAPT* mice also exhibited more hippocampal tau pathology in dystrophic neurites further signifying the greater cognitive phenotype in males, though whether the source of tau was from EC-hippocampus spread [59, 60] or other neuronal inputs was not determined.

### Autophagic disruptions in sleep–wake neurons is a consequence of an early Alzheimer’s disease phenotype

The present study in particular highlights the importance of failed autophagic flux in hypothalamic and LC sleep–wake neurons from the early *App*^NL−G−F^x*MAPT* pathology and preceding significant regional plaque deposition. In particular, LH orexinergic and LPO excitatory neurons were impacted including LH neuronal injury, indicating disruptions in triggers for sleep–wake balance [11, 46]. Calafate et al. recently described melanin-concentrating hormone (MCH) neuronal activity deficits in sleep recovery but no changes in orexinergic neurons, and impaired morphology and plaque-associated dystrophy in hippocampal CA1-projecting MCH axons from 6-months in *App*^NL−G−F^ mice [41]. MCH neurons are sleep-active and increase transition to REM sleep contributing to REM deficits in the AD mice [41, 57]. We and others demonstrate the sensitivity of REM sleep in *App*^NL−G−F^x*MAPT* AD mice [40, 41], though NREM and slow-wave activity as treatable factors in AD are important as well [61–64].

In support of our results, wake-promoting neurons (WPN), including LH orexinergic and LC noradrenergic neurons are vulnerable to AD-related tau pathology in patients, with WPN loss and significant p-tau inclusions in remaining neurons [65]. This signifies the importance of tau and autophagic deficits in sleep–wake neurons. Our study highlights the tau-p62 relationship after sleep disruption in the hypothalamus, and in the LC over *App*^NL−G−F^x*MAPT* disease progression. LC neurodegeneration has been described in the *App*^NL−G−F^ genotype including LC neuronal loss at 9- and 12-months of age, but not earlier [26]. Another study showed no LC neuronal loss at 24-months in *App*^NL−G−F^ mice, with noradrenergic axonal degeneration in the neocortex but not CA1 at 12-months of age, and widespread at 24-months [66]. Sakakibara et al. demonstrated no AT8 + tau in the LC of *App*^NL−G−F^ mice [66], indicating the importance of the human tau knock-in in *App*^NL−G−F^x*MAPT* mice for modelling Aβ-tau-autophagy effects, as we demonstrate significant and progressive LC tau pathology at PHF1 and CP13 epitopes, and the validity to AD patients [65].

Disruption of the autophagic-lysosomal system in *App*^NL−G−F^ single knock-in mice has been described and is similar to our observations, including increased p62 and autophagosomes in the cortex and hippocampus at 12-months [67], and deposition of lysosomal markers from the earliest accumulation of cortical Aβ plaques [68]; which we also demonstrated with lysosomal deposition preceding β-sheet plaque detection. Autophagic impediment in sleep disrupted *MAPT* mice is supported by previous work showing overactivation of the autophagic-lysosomal system [69, 70] and circadian arrhythmicity of autophagic flux [71] in the mouse hippocampus after sleep disruption, and increased Aβ and tau after even one night of sleep loss in humans [11]. To our knowledge ours is the first report of the sleep-autophagy connection in hypothalamic sleep–wake neurons, aligning with the phenotype in *App*^NL−G−F^x*MAPT* mice and in AD patients [65].

Finally, we show that therapeutically activating autophagy in *MAPT* mice improves their sleep recovery after sleep disruption. We propose this is protective against the autophagic impediments that occur during sleep disruption, promoting flux of uncleared p62 + protein through the autophagic-lysosomal pathway, and thereby exerting a behavioral effect on sleep recovery. Our data elucidates an intimate linkage between sleep loss, either by disruption or from AD pathology, and the autophagic-lysosomal system. Given that the sleep staging and electrophysiological impairments during cognitive processing preceded the cognitive phenotype in the AD mice, this work emphasizes the sleep-autophagy relationship as a modifiable disease mechanism in AD and potentially other neurodegenerative disorders.

### Limitations and future directions

There are a few limitations of this study. *Firstly*, the pathological, autophagic and some of the behavioral observations were cross-sectional, and independent animal cohorts were utilized for many of the experiments precluding the usage of intraindividual comparisons across experiments. *Secondly*, the addition of non-transgenic mice and the single *App*^NL−G−F^ line as control groups would have benefited our conclusions; though it has been reported that *MAPT* mice have physiological tau structure and function, and that *App*^NL−G−F^ mice are behaviorally and pathologically (plaque level, tau + neurites) similar to the *App*^NL−G−F^x*MAPT* mice we utilized [16, 19]. However, it is important to note that the present study cannot fully delineate Aβ vs. tau effects, and it is possible that the neuritic-tau and p62-tau pathologies in *App*^NL−G−F^x*MAPT* mice are driven solely by the Aβ pathology and not a synergistic effect with tau humanization. *Thirdly*, dark-cycle NREM sleep time was lower in the present study (in *MAPT* and *App*^NL−G−F^x*MAPT* mice) than has been previously reported in non-transgenic and other AD models [39, 41]. This is likely due to differences in behavioral handling relative to recording onset – the experiment in the present study began near the start of the dark-cycle which may have increased baseline activity levels during this phase – as well as differences in the testing facility and apparatus, and data analysis. *Fourthly*, it is difficult to disentangle the effect of stress during sleep disruption [72]; we utilized an aversive stimuli, yet stress effects could be reduced in future work with less invasive forms of sleep disruption (*i.e.*, gentle handling) [73]. *Fifthly*, the sleep disruption experiment in Fig. 9 would have benefited from electrophysiological measurements of sleep to align to autophagic disruptions, especially given the role of slow waves, and enhancement of slow wave sleep with sodium oxybate, for clearing neurodegenerative proteinopathy (α-synuclein) potentially through improved glymphatic- and cellular proteostasis-mediated protein flux [74].

Future work can utilize single-cell omics as well as single-population induction of autophagic impediments, to investigate molecular factors underlying neuronal vulnerabilities (from genotype, age, sleep disruption, etc.) and to further align the timing and source of Aβ, tau and autophagic aggregates especially those in neuronal processes and neurites. Alignment of the behavioral, pathological and autophagic-lysosomal readouts to the circadian cycle and clock gene expression is another interesting future direction. Critical to our observations from this study and the future therapeutic implications are the impact on neuronal circuitry. Hypothalamic and locus coeruleus neuronal outputs, for example, are complex and have widespread, neuromodulatory effects to regions including the cortex, hippocampus, basal forebrain, thalamus, serotonergic and dopaminergic circuitry etc. [11], many of which are sensitive to AD pathology from early stages. The entorhinal cortex, basal forebrain and locus coeruleus are some of the earliest regions to exhibit tau pathology [2], along with emerging evidence for the sensitivity of hypothalamic WPNs to tau [65], signifying the importance of neuronal, circuitry and regional vulnerabilities to proteinopathy and failed proteostasis for understanding and treating AD.

## Conclusions

### Translational impact of the sleep-autophagy relationship in Alzheimer’s disease

This report highlights the sleep-autophagy dynamics: notably, *1)* the prodromal vulnerability of sleep–wake-regulating neurons to autophagic disruption was aligned to sleep impairments and preceded cognitive decline in the *App*^NL−G−F^x*MAPT* AD mouse model, and *2)* our observation that autophagic flux was dysfunctional after sleep disruption in control mice, and that sleep recovery can be improved with autophagy activation. Sleep is a treatable, modifiable risk factor for AD and most neurodegenerative diseases with a wide selection of therapeutic targets including orexinergic antagonism (suvorexant, lemborexant [75]), anti-depressants (trazodone), non-pharmacological interventions (sleep therapy, light/auditory stimulation, neuromodulation), with varying degrees of interaction with mechanisms of proteostasis (as we recently reviewed: [11]), including targeting autophagy with trehalose as we demonstrate herein. Sleep quality is intimately linked to cognitive function, in particular memory consolidation [11, 76, 77], underlining the promising effect of sleep therapies for AD. Suvorexant, for example, is approved for treating insomnia in mild-to-moderate AD patients [11, 78], has shown cognitive benefits in AD and tauopathy mouse models [79, 80], and reduces tau phosphorylation and Aβ in cognitively unimpaired participants [81], indicating potential preventative or disease modifying effects for AD. The Sleep Trial to Prevent Alzheimer's Disease (SToP-AD) is currently in the recruiting phase with a Suvorexant intervention (ClinicalTrials.gov ID: NCT04629547). Furthermore, beyond EEG measurements, digital wearable and plasma biomarkers may be critical to identify people under sleep stress and those with the greatest potential to benefit from a sleep-targeted therapy [11]. Understanding these neuronal, regional and temporal vulnerabilities to AD pathology and to autophagic disruptions, in alignment with the behavioral phenotype, will aid design of future therapeutic paradigms targeting sleep and autophagy for AD and other neurodegenerative proteinopathies.

## Supplementary Information

Additional file 1. The supplementary file includes Supplementary Tables 1–3, and Supplementary Figs. 1–16

## Data Availability

All data generated and analyzed in this study are reported in the text, figures, figure legends or supplementary information. The raw data that support the findings of this study are available from the corresponding author on reasonable request.

## Abbreviations

3DSD: 3-Day sleep disruption
AD: Alzheimer’s disease
ADL: Activities of daily living
APP: Amyloid precursor protein
Aβ: β-Amyloid
BSA: Bovine serum albumin
CCP3: Cleaved caspase-3
Ctrl: Control
DKI: Double knock-in
EC: Entorhinal cortex
FFT: Fast Fourier Transform
LAMP1: Lysosomal associated membrane protein 1
LC: Locus coeruleus
LH: Lateral hypothalamus
LPO: Lateral preoptic area
MAPT: Microtubule associated protein tau
MCH: Melanin-concentrating hormone
mPOA: Medial preoptic area
NeuN: Neuronal nuclei
NPA: Non-plaque associated
NREM: Non-rapid eye movement
PA: Plaque associated
PBS: Phosphate buffered saline
PFC: Prefrontal cortex
REM: Rapid eye movement
WPN: Wake-promoting neurons
