# Supplementary material for: Autophagic impairment in sleep–wake circuitry is linked to sleep loss at the early stages of Alzheimer’s disease
Source: Mol Neurodegener. 2025 Sep 26;20:99. doi: 10.1186/s13024-025-00877-2 (PMC12465791; doi:10.1186/s13024-025-00877-2)
Supplement: Supplementary file 1 — Additional file 1. The supplementary file includes Supplementary Tables 1–3, and Supplementary Figs. 1–16 [file 13024_2025_877_MOESM1_ESM.pdf]

# Additional file 1

**Supplementary Table 1:** Antibodies and detection reagents utilized for immunostaining.

| Antibody | Dilution | Vendor | Reference # | LOT # (if available) |
|---|---|---|---|---|
| Monoclonal rabbit anti-p62 | 1:400 | Abcam | ab109012 | 1000346-35, 1000346-2, GR3425465-9, 1097355-1 |
| Monoclonal rat anti-LAMP1 | 1:500 | Biolegend | 121602 | B446262 |
| Polyclonal guinea pig anti-NeuN (**except Supplementary Fig. 2**) | 1:500 | Millipore Sigma | ABN90 | 3995040, 3761806 |
| Monoclonal mouse anti-NeuN (**Supplementary Fig. 2**) | 1:500 | Millipore Sigma | MAB377 | 3713321 |
| Monoclonal mouse anti-PHF1 | 1:250 | Courtesy of Dr. Peter Davies | N/A | N/A |
| Monoclonal mouse anti-CP13 | 1:250 | Courtesy of Dr. Peter Davies | N/A | N/A |
| Monoclonal mouse anti-GAD67 | 1:1000 | Millipore Sigma | MAB5406 | 3768064 |
| Monoclonal mouse anti-Orexin A (KK09) | 1:200 | Santa Cruz Biotechnology | sc-80263 | G1524 |
| Monoclonal mouse anti-MAP2 (2a+2b; clone AP-20) | 1:500 | Millipore Sigma | M1406 | 0000284779 |
| Monoclonal mouse anti-β-amyloid (6F/3D) | 1:200 (IF), 1:400 (IHC) | Dako | M0872 | 41354307 |
| Polyclonal rabbit anti-CCP3 | 1:100 | Cell Signaling Technology | 9661 | 47 |
| Goat anti-rabbit Alexafluor 488 | 1:200 | Invitrogen | A11008 | 2765658 |
| Goat anti-guinea pig Alexafluor 568 | 1:200 | Invitrogen | A11075 | 2720368, 2387452 |
| Goat anti-mouse Alexafluor 568 | 1:200 | Invitrogen | A11004 | 2198584, 2855933 |
| Goat anti-rabbit Alexafluor 568 | 1:200 | Invitrogen | A11011 | 2782620 |
| Goat anti-guinea pig Alexafluor 647 | 1:200 | Invitrogen | A21450 | 2978785 |
| Goat anti-rat Alexafluor 647 | 1:200 | Invitrogen | A21247 | 2861859 |
| Biotinylated goat anti-mouse IgG1 | 1:80 | Invitrogen | A10519 | 2647874, 2306800 |
| Streptavidin Alexafluor 647 | 1:200 | Invitrogen | S32357 | 2581823 |
| DAPI | 1:5000 | Roche | 10236276001 | 59795022 |
| Thioflavin-S | 1% wt/volume | Millipore Sigma | T1892 | SHBM5906 |
| ABC kit | Biotinylated horse anti-mouse (1:400), Reagents A and B (1:200) | Vector Laboratories | PK-4002 | ZH1029 |
| DAB peroxidase kit | Kit instructions | Vector Laboratories | SK-4100 | ZH0928 |
| Donkey anti-guinea pig Alexafluor 594 (for NeuN in 4- and 8-month pictures in **Supplementary Figure 1)** | 1:200 | Jackson Immuno Research | 706-585-148 | 155325 |
| Streptavidin Alexafluor 647(for PHF1 in 4- and 8-month pictures in **Supplementary Figure 1)** | 1:200 | Invitrogen | S21374 | 2329429 |

**Supplementary Table 2:** Complete statistics for data reported in **Figures 1-10**.

| Fig. | Test | Statistical parameter or *group comparison* | p-value |
|---|---|---|---|
| **1G** | one-way ANOVA, Holm-Šídák post-hoc | region: F(2,15)=24.30 | **P<0.0001** |
| | | *Neocortex vs. HP* | **P=0.0006** |
| | | *EC vs HP* | **P<0.0001** |
| | | *Neocortex vs. EC* | **P=0.0465** |
| **1H** | multiple unpaired t-tests, Holm-Šídák correction | total HP: t=7.331(df=10) | **P=0.0001** |
| | | CA3: t=3.411(df=10) | **P=0.0439** |
| | | CA1: t=3.090(df=10) | **P=0.0339** |
| | | DG: t=1.739(df=10) | P=0.1127 |
| | | non-cell layer: t=2.703(df=10) | **P=0.0439** |
| **1I** | unpaired t-test | t=6.364(df=10) | **P<0.0001** |
| **1J** | unpaired t-test | t=2.321(df=4) | P=0.0810 |
| **1K** | unpaired t-test | t=3.619(df=4) | **P=0.0224** |
| **1L** | unpaired t-test | t=2.599(df=4) | P=0.0601 |
| **2A - age** | three-way repeated measures ANOVA | age: F(1.418,52.45)=2.519 | P=0.1065 |
| | | genotype: F(1,37)=0.0263 | P=0.8721 |
| | | sex: F(1,37)=5.853 | **P=0.0206** |
| | | age*genotype: F(2,74)=1.055 | P=0.3534 |
| | | age*sex: F(2,74)=1.701 | P=0.1896 |
| | | genotype*sex: F(1,37)=0.7626 | P=0.3882 |
| | | age*genotype*sex: F(2,74)=0.2920 | P=0.7476 |
| **2A - 12-month** | two-way ANOVA, Holm-Šídák post-hoc | genotype*sex: F(1,37)=0.5659 | P=0.4567 |
| | | sex: F(1,37)=5.138 | **P=0.0293** |
| | | genotype: F(1,37)=0.2832 | P=0.5978 |
| | | *DKI: M vs F* | P=0.0732 |
| | | *MAPT: M vs. F* | P=0.2966 |
| **2B - age** | three-way repeated measures ANOVA | age: F(1.872,69.28)=0.0106 | P=0.9865 |
| | | genotype: F(1,37)=2.541 | P=0.1194 |
| | | sex: F(1,37)<0.0001 | P=0.9972 |
| | | age*genotype: F(2,74)=0.3161 | P=0.7299 |
| | | age*sex: F(2,74)=0.4309 | P=0.6516 |
| | | genotype*sex: F(1,37)=3.091 | P=0.0870 |
| | | age*genotype*sex: F(2,74)=0.3637 | P=0.6963 |
| **2B - 12-month** | two-way ANOVA | genotype*sex: F(1,37)=0.4978 | P=0.4849 |
| | | sex: F(1,37)=0.5174 | P=0.4765 |
| | | genotype: F(1,37)=1.204 | P=0.2797 |
| **2C - age** | three-way repeated measures ANOVA | age: F(1.831,67.73)=4.750 | **P=0.0139** |
| | | genotype: F(1,37)=0.4246 | P=0.5187 |
| | | sex: F(1,37)=1.947 | P=0.1713 |
| | | age*genotype: F(2,74)=0.0869 | P=0.9169 |
| | | age*sex: F(2,74)=0.5328 | P=0.5892 |
| | | genotype*sex: F(1,37)=2.390 | P=0.1306 |
| | | age*genotype*sex: F(2,74)=2.074 | P=0.1329 |
| **2C -12-month** | two-way ANOVA, Holm-Šídák post-hoc | genotype*sex: F(1,37)=0.2505 | P=0.6197 |
| | | sex: F(1,37)=5.929 | **P=0.0198** |

| | | | |
|---|---|---|---|
| | | genotype: F(1,37)=0.0183 | P=0.8932 |
| | | *DKI: M vs F* | P=0.0833 |
| | | *MAPT: M vs F* | P=0.1845 |
| **2E** | three-way repeated measures ANOVA | age: F(1.719,63.61)=0.2290 | P=0.7626 |
| | | genotype: F(1,37)=0.9834 | P=0.3278 |
| | | sex: F(1,37)=6.311 | **P=0.0165** |
| | | age*genotype: F(2,74)=3.987 | **P=0.0227** |
| | | age*sex: F(2,74)=2.858 | P=0.0638 |
| | | genotype*sex: F(1,37)=1.090 | P=0.3033 |
| | | age*genotype*sex: F(2,74)=1.564 | P=0.2162 |
| **2E - 12-month** | two-way ANOVA, Holm-Šídák post-hoc | genotype*sex: F(1,37)=2.146 | P=0.1514 |
| | | sex: F(1,37)=6.981 | **P=0.0120** |
| | | genotype: F(1,37)=3.581 | P=0.0663 |
| | | *DKI: M vs F* | **P=0.0113** |
| | | *MAPT: M vs. F* | P=0.4159 |
| **2F** | three-way repeated measures ANOVA | age: F(1.542,57.07)=0.9068 | P=0.3863 |
| | | genotype: F(1,37)=1.593 | P=0.2148 |
| | | sex: F(1,37)=0.5632 | P=0.4577 |
| | | age*genotype: F(2,74)=3.299 | **P=0.0424** |
| | | age*sex: F(2,74)=1.143 | P=0.3243 |
| | | genotype*sex: F(1,37)=2.155 | P=0.1506 |
| | | age*genotype*sex: F(2,74)=0.4915 | P=0.6137 |
| **2F - 12-month** | two-way ANOVA, Holm-Šídák post-hoc | genotype*sex: F(1,37)=3.656 | P=0.0636 |
| | | sex: F(1,37)=2.591 | P=0.1160 |
| | | genotype: F(1,37)=8.433 | **P=0.0062** |
| | | *F: DKI vs. MAPT* | P=0.4924 |
| | | *M: DKI vs. MAPT* | **P=0.0029** |
| **2G - age** | three-way repeated measures ANOVA | age: F(1.690,62.54)=6.455 | **P=0.0045** |
| | | genotype: F(1,37)=0.8142 | P=0.3727 |
| | | sex: F(1,37)=23.64 | **P<0.0001** |
| | | age*genotype: F(2,74)=15.26 | **P<0.0001** |
| | | age*sex: F(2,74)=0.6841 | P=0.5077 |
| | | genotype*sex: F(1,37)=0.5619 | P=0.4582 |
| | | age*genotype*sex: F(2,74)=3.984 | **P=0.0227** |
| **2G - 12-month** | two-way ANOVA, Holm-Šídák post-hoc | genotype*sex: F(1,37)=1.938 | P=0.1722 |
| | | sex: F(1,37)=17.51 | **P=0.0002** |
| | | genotype: F(1,37)=20.73 | **P<0.0001** |
| | | *F: DKI vs. MAPT* | **P=0.0004** |
| | | *M: DKI vs. MAPT* | **P=0.0297** |
| | | *DKI: M vs F* | **P=0.0006** |
| | | *MAPT: M vs. F* | P=0.0586 |
| **3A** | two-way ANOVA, Holm-Šídák post-hoc | genotype: F(1,39)=24.46 | **P<0.0001** |
| | | sex: F(1,39)=13.94 | **P=0.0006** |
| | | genotype*sex: F(1,39)=0.6937 | P=0.4100 |
| | | *F: DKI vs. MAPT* | **P=0.0065** |
| | | *M: DKI vs. MAPT* | **P=0.0004** |

| | | | |
|---|---|---|---|
| | | *DKI: M vs F* | **P=0.0045** |
| | | *MAPT: M vs. F* | **P=0.0495** |
| **3B** | two-way ANOVA | genotype: F(1,39)=2.229 | P=0.1435 |
| | | sex: F(1,39)=0.8052 | P=0.3751 |
| | | genotype*sex: F(1,39)=1.669 | P=0.2040 |
| **3C** | two-way ANOVA, Holm-Šídák post-hoc | sex: F(1,39)=58.05 | **P<0.0001** |
| | | genotype: F(1,39)=2.238 | P=0.1427 |
| | | genotype*sex: F(1,39)=1.489 | P=0.2297 |
| | | *DKI: M vs F* | **P<0.0001** |
| | | *MAPT: M vs. F* | **P<0.0001** |
| **3D** | two-way ANOVA, Holm-Šídák post-hoc | genotype: F(1,39)=8.663 | **P=0.0054** |
| | | sex: F(1,39)=1.425 | P=0.2398 |
| | | genotype*sex: F(1,39)=0.3665 | P=0.5484 |
| | | *F: DKI vs. MAPT* | **P=0.0349** |
| | | *M: DKI vs. MAPT* | P=0.1022 |
| **3E** | two-way ANOVA, Holm-Šídák post-hoc | genotype: F(1,37)=18.22 | **P=0.0001** |
| | | sex: F(1,37)=30.21 | **P<0.0001** |
| | | genotype*sex: F(1,37)=3.329 | P=0.0761 |
| | | *F: DKI vs. MAPT* | **P=0.0003** |
| | | *M: DKI vs. MAPT* | P=0.0886 |
| | | *DKI: M vs F* | **P<0.0001** |
| | | *MAPT: M vs F* | **P=0.0144** |
| **3F** | two-way ANOVA, Holm-Šídák post-hoc | genotype: F(1,37)=40.30 | **P<0.0001** |
| | | sex: F(1,37)=6.511 | **P=0.0150** |
| | | genotype*sex: F(1,37)=1.352 | P=0.2524 |
| | | *F: DKI vs. MAPT* | **P<0.0001** |
| | | *M: DKI vs. MAPT* | **P=0.0007** |
| | | *DKI: M vs. F* | **P=0.0230** |
| | | *MAPT: M vs. F* | P=0.3379 |
| **3J** | multiple unpaired t-tests, Holm-Šídák correction | 4mth wake: t=0.0839(df=18) | P=0.9540 |
| | | 4mth NREM: t=0.2763(df=18) | P=0.9540 |
| | | 4mth REM: t=1.871(df=18) | P=0.2155 |
| | | 12mth wake: t=3.516(df=18) | **P=0.0074** |
| | | 12mth NREM: t=3.443(df=18) | **P=0.0074** |
| | | 12mth: REM: t=2.977(df=18) | **P=0.0081** |
| **3K** | multiple unpaired t-tests, Holm-Šídák correction | 4mth wake: t=0.0578(df=18) | P=0.2069 |
| | | 4mth NREM: t=1.684(df=18) | P=0.9546 |
| | | 4mth REM: t=4.403(df=18) | **P=0.0010** |
| | | 12mth wake: t=3.093(df=18) | **P=0.0125** |
| | | 12mth NREM: t=1.674(df=18) | P=0.1113 |
| | | 12mth: REM: t=5.605(df=18) | **P<0.0001** |
| **3L** | two-way ANOVA, Holm-Šídák post-hoc | genotype: F(1,16)=27.02 | **P<0.0001** |
| | | sex: F(1,16)=9.074 | **P=0.0083** |
| | | genotype*sex: F(1,16)=0.0063 | P=0.9378 |
| | | *F: DKI vs. MAPT* | **P=0.0036** |
| | | *M: DKI vs. MAPT* | **P=0.0036** |

| | | | |
|---|---|---|---|
| | | *DKI: M vs. F* | P=0.0861 |
| | | *MAPT: M vs. F* | P=0.0861 |
| **3M** | two-way ANOVA, Holm-Šídák post-hoc | genotype: F(1,16)=3.679 | P=0.0731 |
| | | sex: F(1,16)=6.007 | **P=0.0261** |
| | | genotype*sex: F(1,16)=1.345 | P=0.2632 |
| | | *DKI: M vs. F* | **P=0.0421** |
| | | *MAPT: M vs. F* | P=0.3747 |
| **3N** | two-way ANOVA, Holm-Šídák post-hoc | genotype: F(1,16)=5.229 | **P=0.0362** |
| | | sex: F(1,16)=0.0923 | P=0.7652 |
| | | genotype*sex: F(1,16)=10.79 | **P=0.0047** |
| | | *F: DKI vs. MAPT* | **P=0.0023** |
| | | *M: DKI vs. MAPT* | P=0.4902 |
| | | *DKI: M vs. F* | P=0.0511 |
| | | *MAPT: M vs. F* | **P=0.0434** |
| **3O** | two-way ANOVA | genotype: F(1,16)=0.0801 | P=0.7808 |
| | | sex: F(1,16)=0.1034 | P=0.7520 |
| | | genotype*sex: F(1,16)=2.783 | P=0.1147 |
| **4D** | two-way ANOVA, Holm-Šídák post-hoc | genotype: F(1,20)=109.2 | **P<0.0001** |
| | | age: F(1,20)=252.3 | **P<0.0001** |
| | | genotype*age: F(1,20)=12.81 | **P=0.0019** |
| | | Holm-Šídák post-hoc: all | **P<0.0001** |
| **4E** | unpaired t-tests | non-plaque-associated: t=2.806(df=4) | **P=0.0485** |
| | | plaque-associated: t=0.0729(df=4) | P=0.9454 |
| **5B** | two-way ANOVA, Holm-Šídák post-hoc | sex: F(1,8)=12.91 | **P=0.0071** |
| | | age: F(1,8)=2866 | **P<0.0001** |
| | | sex*age: F(1,8)=0.0879 | P=0.7744 |
| | | *4mth DKI: M vs. F* | **P=0.0495** |
| | | *12mth DKI: M vs. F* | **P=0.0495** |
| | | *DKI F: 4mth vs. 12mth* | **P<0.0001** |
| | | *DKI M: 4mth vs. 12mth* | **P<0.0001** |
| **5G** | unpaired t-test | t=4.694(df=4) | **P=0.0093** |
| **6B** | two-way ANOVA, Holm-Šídák post-hoc | genotype: F(1,20)=12.93 | **P=0.0018** |
| | | age: F(1,20)=11.68 | **P=0.0027** |
| | | genotype*age: F(1,20)=0.0759 | P=0.7858 |
| | | *4mth DKI vs. 4mth MAPT* | **P=0.0252** |
| | | *12mth DKI vs. 12mth MAPT* | **P=0.0293** |
| | | *4mth DKI vs. 12mth DKI* | **P=0.0380** |
| | | *4mth MAPT vs. 12mth MAPT* | **P=0.0331** |
| **6C** | two-way ANOVA, Holm-Šídák post-hoc | genotype: F(1,20)=42.64 | **P<0.0001** |
| | | age: F(1,20)=0.06357 | P=0.8035 |
| | | genotype*age: F(1,20)=1.331 | P=0.2622 |
| | | *4mth DKI vs. 4mth MAPT* | **P=0.0011** |
| | | *12mth DKI vs. 12mth MAPT* | **P<0.0001** |
| **6D** | two-way ANOVA, Holm-Šídák post-hoc | genotype: F(1,20)=19.60 | **P=0.0003** |
| | | age: F(1,20)=0.1508 | P=0.7019 |
| | | genotype*age: F(1,20)=1.032 | P=0.3219 |

| | | | |
|---|---|---|---|
| | | *4mth DKI vs. 4mth MAPT* | **P=0.0256** |
| | | *12mth DKI vs. 12mth MAPT* | **P=0.0020** |
| **6E** | two-way ANOVA, Holm-Šídák post-hoc | genotype: F(1,20)=31.57 | **P<0.0001** |
| | | age: F(1,20)=2.004 | P=0.1722 |
| | | genotype*age: F(1,20)=0.6327 | P=0.4357 |
| | | *4mth DKI vs. 4mth MAPT* | **P=0.0028** |
| | | *12mth DKI vs. 12mth MAPT* | **P=0.0004** |
| **6F** | two-way ANOVA | genotype: F(1,20)=2.965 | P=0.1005 |
| | | age: F(1,20)=0.3106 | P=0.5835 |
| | | genotype*age: F(1,20)=1.489 | P=0.2366 |
| **6G** | two-way ANOVA, Holm-Šídák post-hoc | genotype: F(1,20)=34.00 | **P<0.0001** |
| | | age: F(1,20)=41.68 | **P<0.0001** |
| | | genotype*age: F(1,20)=17.53 | **P=0.0005** |
| | | *4mth DKI vs. 4mth MAPT* | P=0.2588 |
| | | *12mth DKI vs. 12mth MAPT* | **P <0.0001** |
| | | *4mth DKI vs. 12mth DKI* | **P<0.0001** |
| | | *4mth MAPT vs. 12mth MAPT* | P=0.1243 |
| **6L** | two-way ANOVA, Holm-Šídák post-hoc | genotype: F(1,8)=415.0 | **P<0.0001** |
| | | age: F(1,8)=514.4 | **P<0.0001** |
| | | genotype*age: F(1,8)=410.2 | **P<0.0001** |
| | | *4mth DKI vs. 4mth MAPT* | P=0.9358 |
| | | *12mth DKI vs. 12mth MAPT* | **P <0.0001** |
| | | *4mth DKI vs. 12mth DKI* | **P<0.0001** |
| | | *4mth MAPT vs. 12mth MAPT* | P=0.1247 |
| **7C** | two-way ANOVA, Holm-Šídák post-hoc | genotype: F(1,20)=61.00 | **P<0.0001** |
| | | age: F(1,20)=45.01 | **P<0.0001** |
| | | genotype*age: F(1,20)=19.57 | **P=0.0003** |
| | | *4mth DKI vs. 4mth MAPT* | **P=0.0266** |
| | | *12mth DKI vs. 12mth MAPT* | **P<0.0001** |
| | | *4mth DKI vs. 12mth DKI* | **P<0.0001** |
| | | *4mth MAPT vs. 12mth MAPT* | P=0.1219 |
| **8C** | unpaired t-test | t=0.6147(df=14) | P=0.5486 |
| **8D** | unpaired t-test | t=2.355(df=14) | **P=0.0336** |
| **8E** | unpaired t-test | t=2.445(df=14) | **P=0.0283** |
| **8F** | unpaired t-test | t=2.418(df=14) | **P=0.0298** |
| **9A - light-cycle** | two-way repeated measures ANOVA | time*SD: F(16,256)=1.555 | P=0.0813 |
| | | time: F(6.057,96.91)=3.665 | **P=0.0025** |
| | | SD: F(1,16)=0.6661 | P=0.4264 |
| | | subject: F(16,256)=6.602 | **P<0.0001** |
| **9A - dark-cycle** | two-way repeated measures ANOVA, Holm-Šídák post-hoc | time*SD: F(17,272)=3.587 | **P<0.0001** |
| | | time: F(7.162,114.6)=4.522 | **P=0.0002** |
| | | SD: F(1,16)=19.32 | **P=0.0005** |
| | | subject: F(16,272)=1.633 | P=0.0602 |
| | | *MAPT-Ctrl vs. MAPT-3DSD – h12-14* | P=0.0504 |
| | | *MAPT-Ctrl vs. MAPT-3DSD – h14-16* | P=0.3593 |
| | | *MAPT-Ctrl vs. MAPT-3DSD – h16-18* | P=0.9025 |

| | | | |
|---|---|---|---|
| | | *MAPT-Ctrl vs. MAPT-3DSD – h18-20* | P=0.4642 |
| | | *MAPT-Ctrl vs. MAPT-3DSD – h20-22* | P=0.7275 |
| | | *MAPT-Ctrl vs. MAPT-3DSD – h22-24* | P=0.9535 |
| | | *MAPT-Ctrl vs. MAPT-3DSD – h36-38* | **P=0.0361** |
| | | *MAPT-Ctrl vs. MAPT-3DSD – h38-40* | P=0.3593 |
| | | *MAPT-Ctrl vs. MAPT-3DSD – h40-42* | P=0.1466 |
| | | *MAPT-Ctrl vs. MAPT-3DSD – h42-44* | P=0.9535 |
| | | *MAPT-Ctrl vs. MAPT-3DSD – h44-46* | P=0.3593 |
| | | *MAPT-Ctrl vs. MAPT-3DSD – h46-48* | P=0.3777 |
| | | *MAPT-Ctrl vs. MAPT-3DSD – h60-62* | **P=0.0273** |
| | | *MAPT-Ctrl vs. MAPT-3DSD – h62-64* | **P=0.0275** |
| | | *MAPT-Ctrl vs. MAPT-3DSD – h64-66* | P=0.0668 |
| | | *MAPT-Ctrl vs. MAPT-3DSD – h66-68* | P=0.9535 |
| | | *MAPT-Ctrl vs. MAPT-3DSD – h68-70* | P=0.7275 |
| | | *MAPT-Ctrl vs. MAPT-3DSD – h70-72* | P=0.9535 |
| **9B** | multiple unpaired t-tests, Holm-Šídák correction | light-cycle: t=0.8162(df=16) | P=0.4264 |
| | | dark-cycle: t=4.396(df=16) | **P=0.0009** |
| **9E** | unpaired t-test | t=2.702(df=7) | **P=0.0305** |
| **9F** | unpaired t-test | t=0.8950(df=7) | P=0.4005 |
| **9I** | unpaired t-test | t=4.291(df=16) | **P=0.0006** |
| **9J** | Pearson correlation, linear regression | $r^2$=0.3127; Y=2.260*X-3.809; F(1,16)=7.278 | **P=0.0158** |
| **10C** | unpaired t-test | t=2.487(df=18) | **P=0.0229** |
| **10D** | two-way repeated measures ANOVA, Holm-Šídák post-hoc | time*treatment: F(5,90)=1.889 | P=0.1039 |
| | | time: F(3.248,58.47)=8.316 | **P<0.0001** |
| | | treatment: F(1,18)=6.187 | **P=0.0229** |
| | | subject: F(18,90)=0.9021 | P=0.5776 |
| | | *MAPT-suc vs. MAPT-tre – h0-2* | P=0.5039 |
| | | *MAPT-suc vs. MAPT-tre – h2-4* | P=0.2284 |
| | | *MAPT-suc vs. MAPT-tre – h4-6* | P=0.9784 |
| | | *MAPT-suc vs. MAPT-tre – h6-8* | P=0.9908 |
| | | *MAPT-suc vs. MAPT-tre – h8-10* | P=0.9908 |
| | | *MAPT-suc vs. MAPT-tre – h10-12* | **P=0.0201** |
| **10E** | unpaired t-test | t=0.0308(df=18) | P=0.9758 |

**Abbreviations:** 3DSD: 3-day sleep disruption; Ctrl: control; DG: dentate gyrus; DKI: double knock-in (*App*$^{\text{NL-G-F}}$x*MAPT*); EC: entorhinal cortex; F: female; h: hour; HP: hippocampus; M: male; NREM: non-rapid eye movement; REM: rapid eye movement; SD: sleep disruption; suc: sucrose; tre: trehalose.

**Supplementary Table 3:** Complete statistics for data reported in **Supplementary Figures 3-5.**

| **Fig.** | **Test** | **Statistical parameter or *group comparison*** | **p-value** |
|---|---|---|---|
| **S3** | two-way ANOVA, Holm-Šídák post-hoc | genotype*sex: F(1,37)=1.961 | P=0.1698 |
| | | sex: F(1,37)=6.177 | **P=0.0176** |
| | | genotype: F(1,37)=0.5100 | P=0.4796 |
| | | *DKI: M vs F* | **P=0.0169** |
| | | *MAPT: M vs F* | P=0.4530 |
| **S4A latency** | three-way repeated measures ANOVA | trial: F(2.456,95.80)=3.084 | **P=0.0402** |
| | | genotype: F(1,39)=1.127 | P=0.2950 |
| | | sex: F(1,39)=4.462 | **P=0.0411** |
| | | trial*genotype: F(3,117)=0.2903 | P=0.8323 |
| | | trial*sex: F(3,117)=1.218 | P=0.3063 |
| | | genotype*sex: F(1,39)=0.1632 | P=0.6884 |
| | | trial*genotype*sex: F(3,117)=0.3346 | P=0.8003 |
| **S4A errors** | three-way repeated measures ANOVA | trial: F(2.559,99.81)=9.369 | **P<0.0001** |
| | | genotype: F(1,39)=0.0416 | P=0.8395 |
| | | sex: F(1,39)=0.6573 | P=0.4224 |
| | | trial*genotype: F(3,117)=1.175 | P=0.3223 |
| | | trial*sex: F(3,117)=0.4621 | P=0.7093 |
| | | genotype*sex: F(1,39)=2.516 | P=0.1208 |
| | | trial*genotype*sex: F(3,117)=0.5630 | P=0.6405 |
| **S4B** | two-way ANOVA | genotype*sex: F(1,39)=0.0452 | P=0.8327 |
| | | sex: F(1,39)=0.2116 | P=0.6481 |
| | | genotype: F(1,39)=0.2131 | P=0.6469 |
| **S4C latency** | three-way repeated measures ANOVA | trial: F(2.345,91.47)=8.563 | **P=0.0002** |
| | | genotype: F(1,39)=0.0018 | P=0.9660 |
| | | sex: F(1,39)=4.460 | **P=0.0412** |
| | | trial*genotype: F(3,117)=0.5580 | P=0.6438 |
| | | trial*sex: F(3,117)=0.5985 | P=0.6173 |
| | | genotype*sex: F(1,39)=0.1064 | P=0.7460 |
| | | trial*genotype*sex: F(3,117)=0.0646 | P=0.9785 |
| **S4C errors** | three-way repeated measures ANOVA | trial: F(2.620,102.2)=1.378 | P=0.2557 |
| | | genotype: F(1,39)=0.3130 | P=0.5791 |
| | | sex: F(1,39)=0.2190 | P=0.6424 |
| | | trial*genotype: F(3,117)=3.377 | **P=0.0207** |
| | | trial*sex: F(3,117)=0.5139 | P=0.6735 |
| | | genotype*sex: F(1,39)=0.3829 | P=0.5396 |
| | | trial*genotype*sex: F(3,117)=0.0966 | P=0.9618 |
| **S4D latency** | three-way repeated measures ANOVA | trial: F(3,117)=1.642 | P=0.1835 |
| | | genotype: F(1,39)=0.1788 | P=0.6747 |
| | | sex: F(1,39)=2.506 | P=0.1215 |
| | | trial*genotype: F(3,117)=0.3865 | P=0.7629 |
| | | trial*sex: F(3,117)=0.9027 | P=0.4421 |
| | | genotype*sex: F(1,39)=1.278 | P=0.2652 |
| | | trial*genotype*sex: F(3,117)=0.6018 | P=0.6151 |
| **S4D errors** | | trial: F(2.395,93.42)=0.9471 | P=0.4052 |

| | | | |
|---|---|---|---|
| | three-way repeated measures ANOVA | genotype: F(1,39)=2.020 | P=0.1632 |
| | | sex: F(1,39)=0.2519 | P=0.6186 |
| | | trial*genotype: F(3,117)=0.5788 | P=0.6301 |
| | | trial*sex: F(3,117)=0.2191 | P=0.8830 |
| | | genotype*sex: F(1,39)=2.339 | P=0.1342 |
| | | trial*genotype*sex: F(3,117)=0.1465 | P=0.9318 |
| **S4E** | two-way ANOVA | genotype*sex: F(1,39)=3.820 | P=0.0578 |
| | | sex: F(1,39)=0.0115 | P=0.9152 |
| | | genotype: F(1,39)=0.0919 | P=0.7634 |
| **S4F latency** | three-way repeated measures ANOVA | trial: F(1.965,76.64)=0.9782 | P=0.3794 |
| | | genotype: F(1,39)=0.0013 | P=0.9715 |
| | | sex: F(1,39)=3.850 | P=0.0569 |
| | | trial*genotype: F(3,117)=0.3284 | P=0.8048 |
| | | trial*sex: F(3,117)=1.288 | P=0.2817 |
| | | genotype*sex: F(1,39)=0.3190 | P=0.5754 |
| | | trial*genotype*sex: F(3,117)=0.2187 | P=0.8832 |
| **S4F errors** | three-way repeated measures ANOVA | trial: F(2.729,106.4)=0.3876 | P=0.7433 |
| | | genotype: F(1,39)=0.2537 | P=0.6173 |
| | | sex: F(1,39)=0.0808 | P=0.7777 |
| | | trial*genotype: F(3,117)=2.264 | P=0.0846 |
| | | trial*sex: F(3,117)=3.487 | **P=0.0180** |
| | | genotype*sex: F(1,39)=0.4139 | P=0.5238 |
| | | trial*genotype*sex: F(3,117)=2.068 | P=0.1082 |
| **S4G latency** | three-way repeated measures ANOVA | trial: F(1.945,71.97)=0.1391 | P=0.8650 |
| | | genotype: F(1,37)=0.2832 | P=0.5978 |
| | | sex: F(1,37)=5.138 | **P=0.0293** |
| | | trial*genotype: F(3,111)=0.8675 | P=0.4603 |
| | | trial*sex: F(3,111)=0.5861 | P=0.6254 |
| | | genotype*sex: F(1,37)=0.5659 | P=0.4567 |
| | | trial*genotype*sex: F(3,111)=0.9939 | P=0.3985 |
| **S4G errors** | three-way repeated measures ANOVA | trial: F(2.704,100.0)=0.2479 | P=0.8431 |
| | | genotype: F(1,37)=1.204 | P=0.2797 |
| | | sex: F(1,37)=0.5174 | P=0.4765 |
| | | trial*genotype: F(3,111)=0.3116 | P=0.8169 |
| | | trial*sex: F(3,111)=0.2473 | P=0.8631 |
| | | genotype*sex: F(1,37)=0.4978 | P=0.4849 |
| | | trial*genotype*sex: F(3,111)=0.6031 | P=0.6143 |
| **S4H** | two-way ANOVA, Holm-Šídák post-hoc | genotype*sex: F(1,37)=0.2505 | P=0.6197 |
| | | sex: F(1,37)=5.929 | **P=0.0198** |
| | | genotype: F(1,37)=0.0183 | P=0.8932 |
| | | *DKI: M vs F* | P=0.0833 |
| | | *MAPT: M vs F* | P=0.1845 |
| **S4I latency** | three-way repeated measures ANOVA | trial: F(2.563,94.82)=3.020 | **P=0.0411** |
| | | genotype: F(1,37)=3.581 | P=0.0663 |
| | | sex: F(1,37)=6.981 | **P=0.0120** |

| | | | |
|---|---|---|---|
| | | trial*genotype: $F(3,111)=1.324$ | $P=0.2702$ |
| | | trial*sex: $F(3,111)=1.405$ | $P=0.2450$ |
| | | genotype*sex: $F(1,37)=2.146$ | $P=0.1514$ |
| | | trial*genotype*sex: $F(3,111)=1.182$ | $P=0.3201$ |
| **S4I errors** | three-way repeated measures ANOVA | trial: $F(3,111)=0.7806$ | $P=0.5072$ |
| | | genotype: $F(1,37)=8.433$ | **$P=0.0062$** |
| | | sex: $F(1,37)=2.591$ | $P=0.1160$ |
| | | trial*genotype: $F(3,111)=0.1563$ | $P=0.9254$ |
| | | trial*sex: $F(3,111)=4.565$ | **$P=0.0047$** |
| | | genotype*sex: $F(1,37)=3.656$ | $P=0.0636$ |
| | | trial*genotype*sex: $F(3,111)=0.4530$ | $P=0.7157$ |
| **S5A** | three-way repeated measures ANOVA | time: $F(2,78)=29.82$ | **$P<0.0001$** |
| | | genotype: $F(1,39)=9.330$ | **$P=0.0041$** |
| | | sex: $F(1,39)=3.612$ | $P=0.0648$ |
| | | time*genotype: $F(2,78)=4.768$ | **$P=0.0111$** |
| | | time*sex: $F(2,78)=0.1415$ | $P=0.8683$ |
| | | genotype*sex: $F(1,39)=2.437$ | $P=0.1266$ |
| | | time*genotype*sex: $F(2,78)=1.372$ | $P=0.2597$ |
| **S5B** | three-way repeated measures ANOVA | time: $F(2,78)=22.22$ | **$P<0.0001$** |
| | | genotype: $F(1,39)=0.0959$ | $P=0.7585$ |
| | | sex: $F(1,39)=10.98$ | **$P=0.0020$** |
| | | time*genotype: $F(2,78)=0.3211$ | $P=0.7263$ |
| | | time*sex: $F(2,78)=5.565$ | **$P=0.0055$** |
| | | genotype*sex: $F(1,39)=0.6566$ | $P=0.4227$ |
| | | time*genotype*sex: $F(2,78)=0.2836$ | $P=0.7539$ |
| **S5C** | three-way repeated measures ANOVA | time: $F(2,74)=11.05$ | **$P<0.0001$** |
| | | genotype: $F(1,37)=20.73$ | **$P<0.0001$** |
| | | sex: $F(1,37)=17.51$ | **$P=0.0002$** |
| | | time*genotype: $F(2,74)=1.499$ | $P=0.2301$ |
| | | time*sex: $F(2,74)=0.4315$ | $P=0.6511$ |
| | | genotype*sex: $F(1,37)=1.938$ | $P=0.1722$ |
| | | time*genotype*sex: $F(2,74)=1.574$ | $P=0.2141$ |

**Abbreviations:** DKI: double knock-in (*App*$^{NL\text{-}G\text{-}F}$x*MAPT*); F: female; M: male.

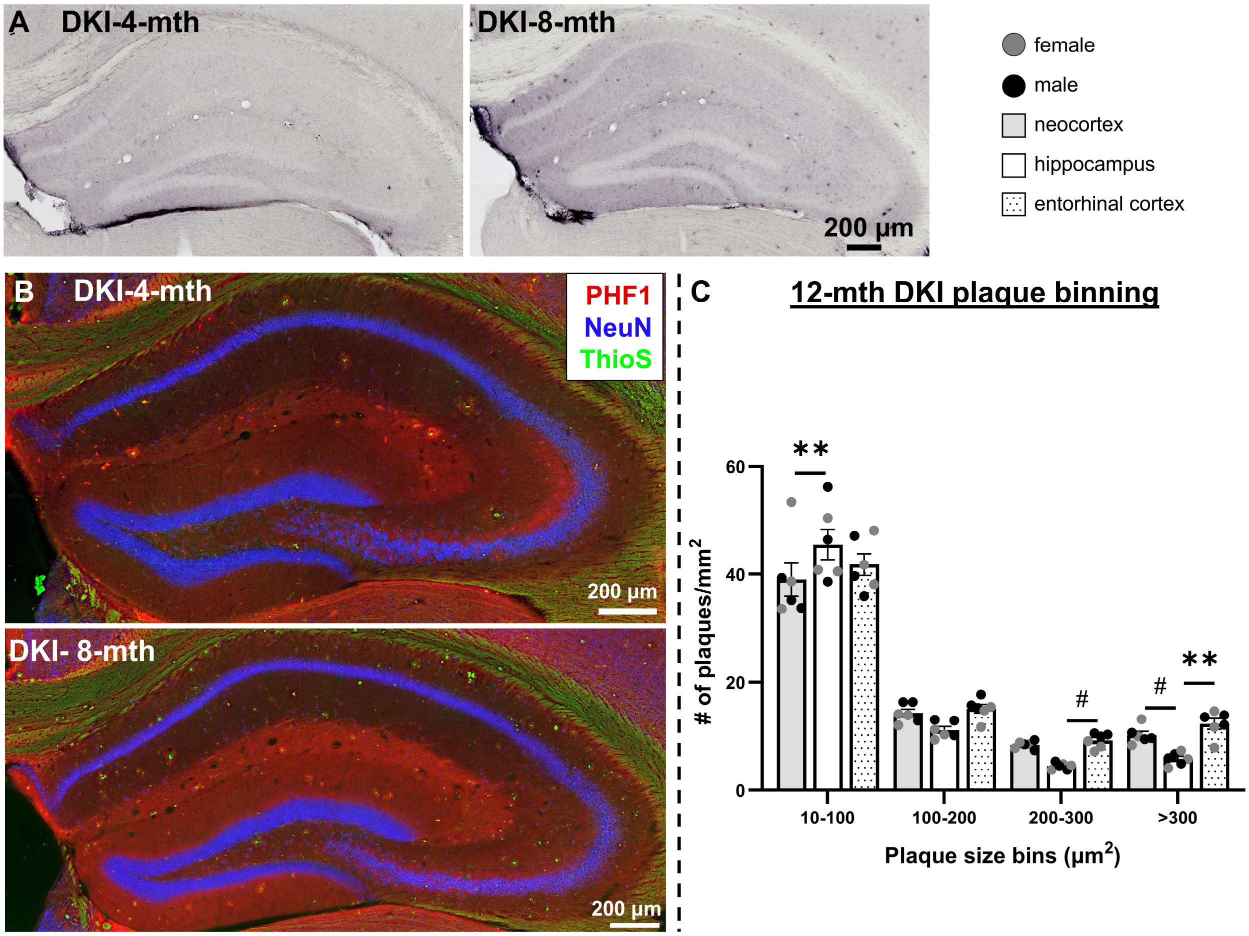


**Supplementary Fig. 1: Representative pathological images at 4- and 8-months in in *App*$^{NL\text{-}G\text{-}F}$x*MAPT* mice and Aβ plaque binning at 12-months. A,B** Representative 4- and 8-month immunostaining for 6F/3D (black), and for NeuN (blue), PHF1 (red) and Thioflavin-S (green) demonstrating progressive plaque accumulation, association of tau with plaques, and the lack of thinning in neuronal cell layers. **C** Thioflavin-S plaques were quantified in 12-month *App*$^{NL\text{-}G\text{-}F}$x*MAPT*s (DKI) and binned by size in the neocortex, hippocampus and entorhinal cortex. Significant effects of size binning (two-way ANOVA: $F(3,60)=380.8$, **P<0.0001**), region ($F(2,60)=3.957$, **P=0.0243**) and size*region ($F(6,60)=3.874$, **P=0.0025**) were detected. The hippocampus had significantly more small plaques (10-100 µm) than the neocortex (Holm-Šídák post-hoc: **P=0.0066**), and the cortical regions had more larger plaques than the hippocampus (200-300 µm, entorhinal cortex vs. hippocampus: P=0.0708; >300 µm, neocortex vs. hippocampus: P=0.0683; >300 µm, entorhinal cortex vs. hippocampus: **P=0.0069**). Data are presented as mean+/-SEM; *n*=3/sex (**A**) and *n*=3/age (**B,C**). #P<0.10, **P<0.01.

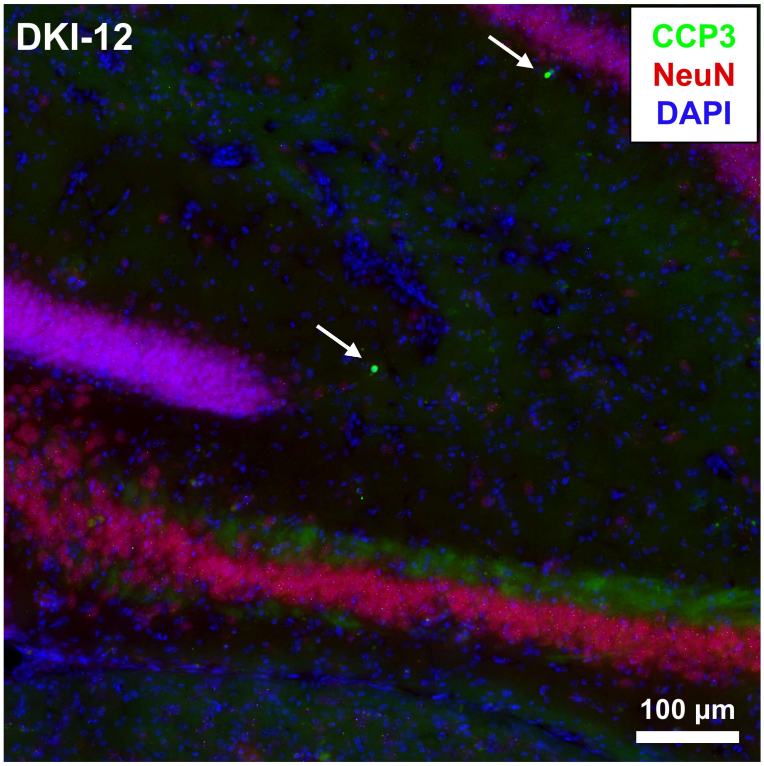


**Supplementary Fig. 2: Cleaved caspase-3 (CCP3) staining does not reflect loss of neuronal nuclei (NeuN) signal.** Representative CCP3 (green) image demonstrating positive signal that does not account for the loss of NeuN in the hippocampus (see **Fig. 1**) or in the hypothalamus (see **Fig. 6**) observed in *App*$^{NL\text{-}G\text{-}F}$x*MAPT* mice.

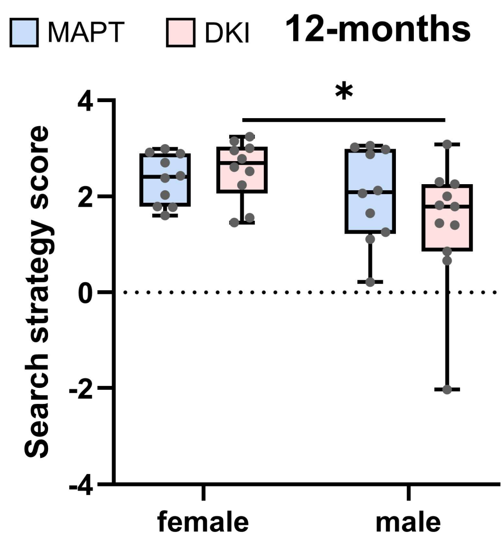


**Supplementary Fig. 3: Male *App*$^{NL\text{-}G\text{-}F}$x*MAPT* mice at 12-months exhibit impairment in the complexity of their search strategies utilized during the spatial memory probe.** Extended Barnes maze data; see also **Fig. 2C**. The average search strategy score across the 3-minute trial was calculated - direct and corrected strategies (score range ~3-5), long correction and focused search (score range ~1-3), serial search (score range ~0-1) and random (score range <0). Late-stage male *App*$^{NL\text{-}G\text{-}F}$x*MAPT* mice utilize significantly less complex search strategies than female *App*$^{NL\text{-}G\text{-}F}$x*MAPT* mice. Data are represented as box (line: median) and whisker (min-max) plots. *P<0.05. Statistical analysis was with two-way ANOVA, Holm-Šídák post-hoc; *n*=10-11/sex/genotype/age. See **Supplementary Table 3** for complete statistics.

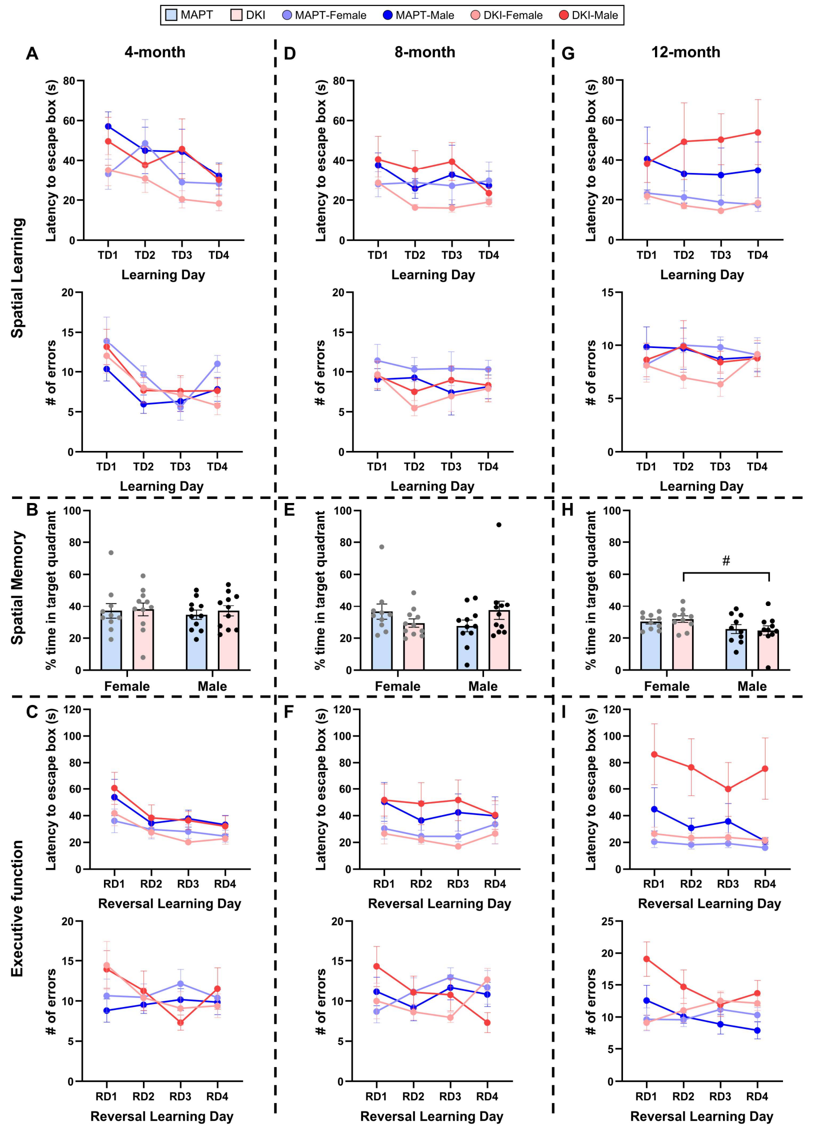


**Supplementary Fig. 4: Barnes maze data separated trial-by-trial and by age highlighting the deficit in 12-month *App*$^{\text{NL-G-F}}$x*MAPT* mice; in particular the male mice.** Extended Barnes maze data; see also **Fig. 2A-F.** **A-C** No *App*$^{\text{NL-G-F}}$x*MAPT* (DKI) vs. *MAPT* genotype differences were detected in learning (4 trial days (TD), latency to find the escape box, number of errors), memory (1 probe, % time searching target quadrant) or executive function (4 reversal days (RD), latency to find the escape box, number of errors) at 4-months of age; the trial*genotype effect for RD errors was significant. **D-F** No significant genotype differences were detected in cognition at 8-months either, with a trend to a genotype*sex interaction effect in spatial memory. Males were significantly slower to find the escape box than females at 4-months in learning and reversal learning trials, though they did not make more errors. In the 8-month reversal trials, there was a trend to a sex effect in the latency to escape, and a significant trial*sex effect in number of errors. **G** 12-month learning was not impacted by genotype in the latency or errors, with significantly slower performance in males. **H** Males of both genotypes spent less time searching the target quadrant in the memory probe at 12-months, with a trend to reduction in male vs. female *App*$^{\text{NL-G-F}}$x*MAPT* mice. **I** Impairment in executive function (longer latency to escape (P=0.0663) and greater number of errors (**P=0.0062**)) is evident in 12-month *App*$^{\text{NL-G-F}}$x*MAPT* mice, specifically in male mice. Data are presented as mean+/-SEM; *n*=10-11/sex/genotype/age. #P<0.10. Statistical analysis was with three-way repeated measures ANOVA (**A,C,D,F,G,I**) or two-way ANOVA, Holm-Šídák post-hoc when appropriate (**B,E,H**); see **Supplementary Table 3** for complete statistics.

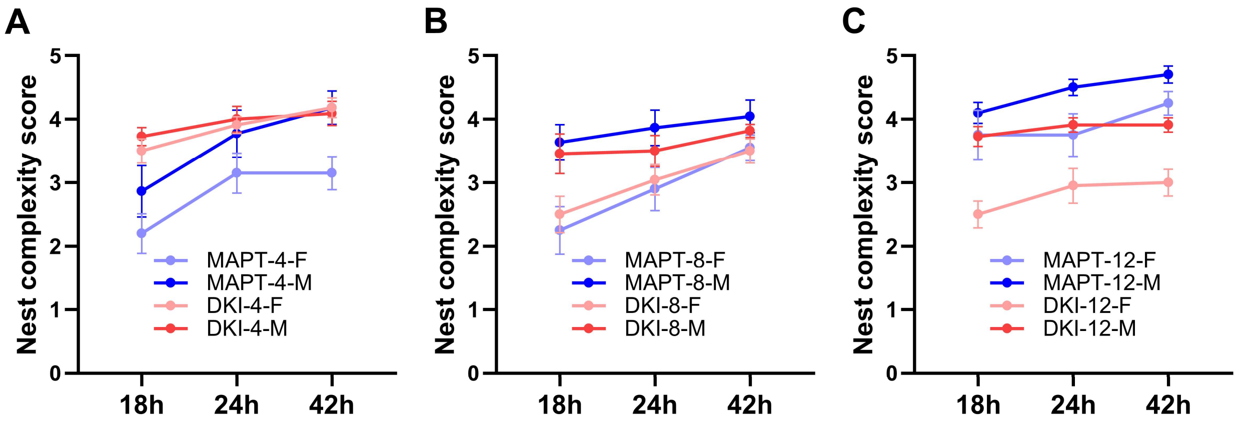


**Supplementary Fig. 5: Nesting data separated by timepoint and by age.** Extended activities of daily living data; see also **Fig. 2G**. **A** *App*$^{\text{NL-G-F}}$x*MAPT* (DKI) built significantly more complex nests than *MAPT*s at 4-months; sex effect was trending. **B** 8-month nesting significantly changed by sex (males>females), but not by genotype. **C** 12-month *App*$^{\text{NL-G-F}}$x*MAPT* mice built significantly less complex nests than *MAPT*s, as did females when compared to males. Data are presented as mean+/-SEM; *n*=10-11/sex/genotype/age. Statistical analysis was with three-way repeated measures ANOVA; see **Supplementary Table 3** for complete statistics.

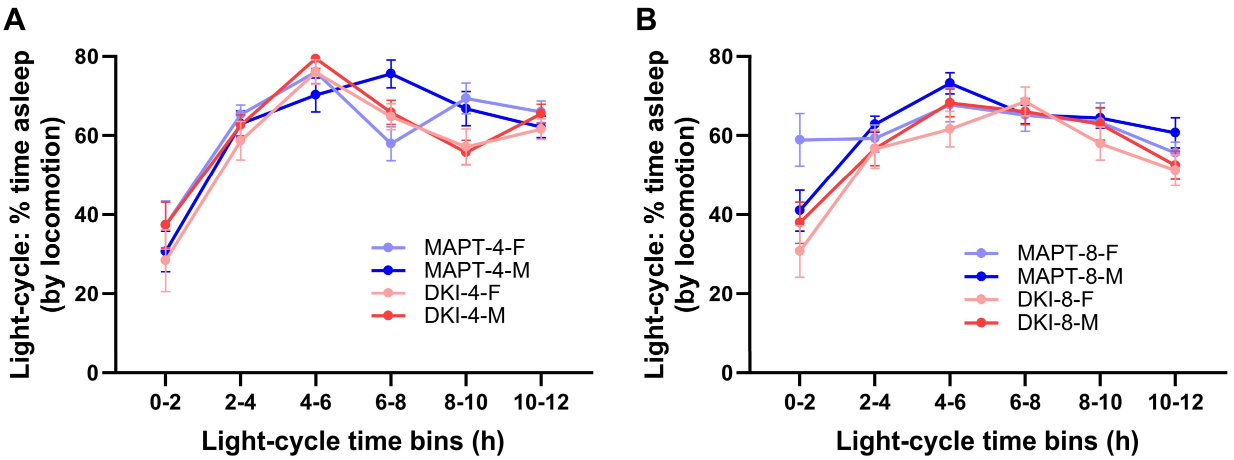


**Supplementary Fig. 6: Light-cycle sleep activity patterns at 4- and 8-months of age.** Sleep activity patterns were measured in 2-hour bins in PhenoTypers, in *MAPT* and *App*$^{\text{NL-G-F}}$x*MAPT* (DKI) mice. **A** 4-month mice have no overt genotype effects with a gradual increase in sleep from the start of the light-cycle to a peak halfway through and then a slight decline towards the end of the light-cycle. **B** Similar patterns were observed at 8-months, with slightly less sleep in the first 2-hours in female *App*$^{\text{NL-G-F}}$x*MAPT*s. Data are presented as mean+/-SEM; *n*=10-11/sex/genotype/age.

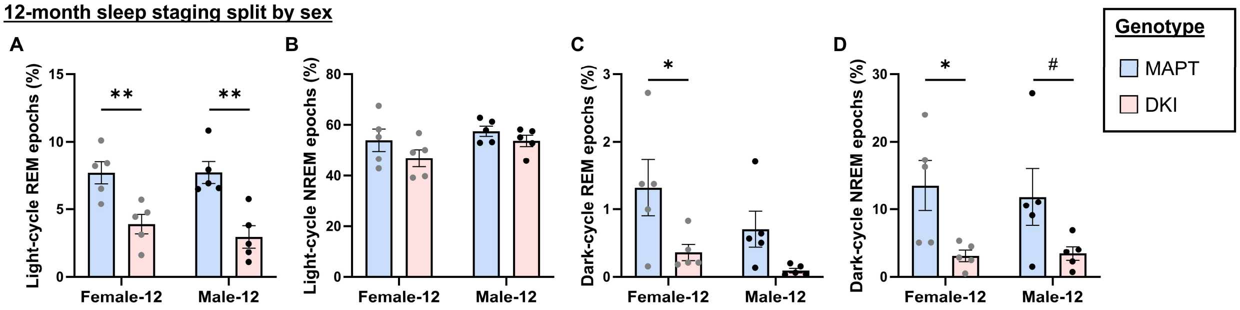


**Supplementary Fig. 7: No sex differences were observed in sleep staging by EEG in 12-month *MAPT* and *App*$^{NL\text{-}G\text{-}F}$x*MAPT* mice.** Sleep staging data in 12-month *MAPT* and *App*$^{NL\text{-}G\text{-}F}$x*MAPT* (DKI) mice from **Fig. 3J,K** was re-graphed and split by sex to assess potential sex differences. **A** A significant effect of genotype (F(1,16)=29.15, **P<0.0001**; females: **P=0.0037**, males: **P=0.0012**), but not sex (F(1,16)=0.3313, P=0.5729) or sex*genotype interaction (F(1,16)=0.3671, P=0.5531) was detected on light-cycle REM sleep time. **B** No significant differences were detected by genotype (F(1,16)=2.964, P=0.1044), sex (F(1,16)=2.745, P=0.1171) or sex*genotype interaction (F(1,16)=0.2876, P=0.5991) on light-cycle NREM sleep time. **C** A significant effect of genotype (F(1,16)=9.585, **P=0.0069**; females: **P=0.0334**, males: P=0.1065), but not sex (F(1,16)=3.005, P=0.1023) or sex*genotype interaction (F(1,16)=0.4583, P=0.5081) was detected on dark-cycle REM sleep time. **D** A significant effect of genotype (F(1,16)=10.66, **P=0.0049**; females: **P=0.0415**, males: P=0.0565), but not sex (F(1,16)=0.0507, P=0.8247) or sex*genotype interaction (F(1,16)=0.1270, P=0.7262) was detected on dark-cycle NREM sleep time. Data are presented as mean+/-SEM; *n*=5/sex/genotype. *P<0.01; **P<0.01. Statistical analysis was with two-way ANOVA, Holm-Šídák post-hoc when appropriate.

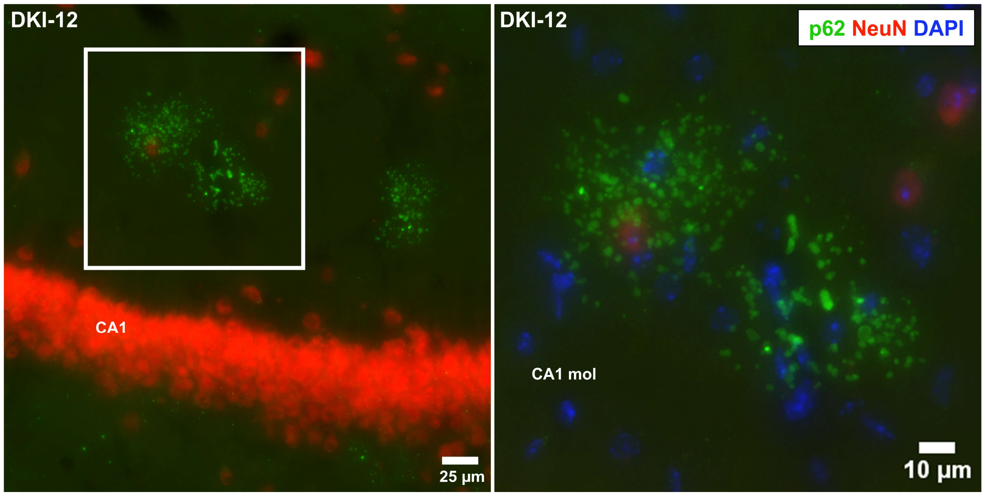


**Supplementary Fig. 8: p62 aggregates are not typically seen in the cytoplasm in the hippocampus.** Immunostaining was conducted for p62 (green), NeuN (red) and DAPI (blue). Representative 12-month *App*$^{NL\text{-}G\text{-}F}$x*MAPT* (DKI) pictures demonstrating the rare association of p62 aggregates around nuclear cell (DAPI) and specifically neuronal (NeuN) staining. This is consistent in *MAPT*s and at 4-months in *App*$^{NL\text{-}G\text{-}F}$x*MAPT*s. Representative images from *n*=3/sex/genotype/age.

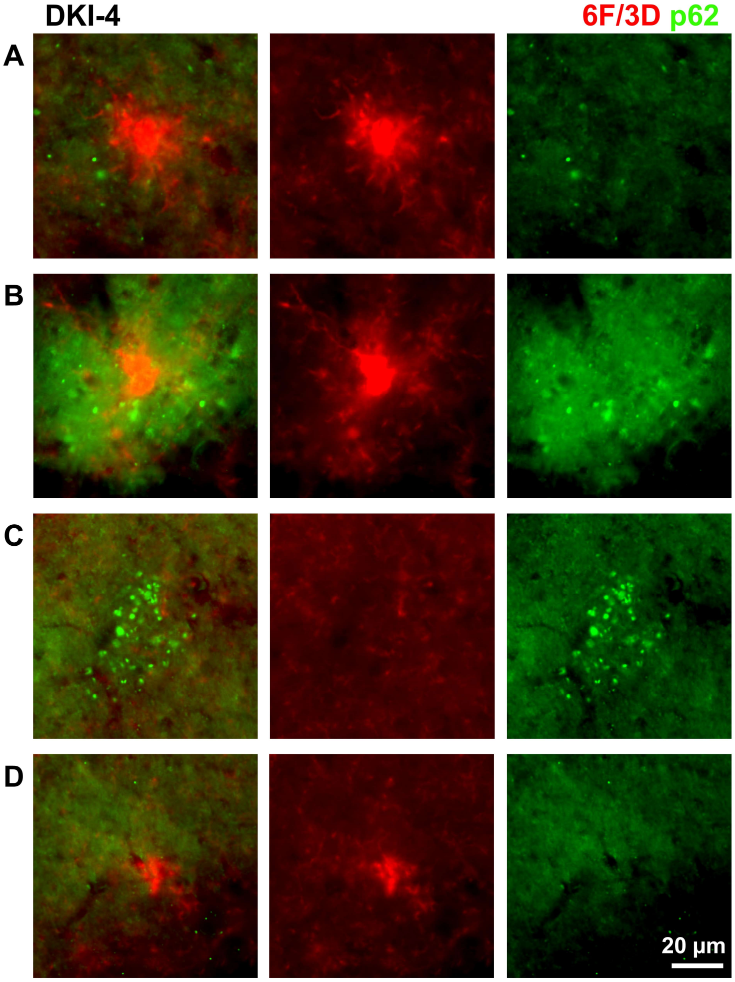


**Supplementary Fig. 9: Hippocampal p62 clusters in *App*$^{\text{NL-G-F}}$x*MAPT* mice were both plaque- and non-plaque-associated.** Images are related to **Fig. 4B** and **4C**. **A** Representative Aβ plaque (6F/3D, red) with minimal surrounding p62 (green) aggregation (not enough to classify as a p62 cluster). **B** Representative Aβ plaque with an associated p62 cluster. **C** Representative non-plaque-associated p62 cluster. **D** Representative Aβ plaque without associated p62 pathology. Images are from 4-month *App*$^{\text{NL-G-F}}$x*MAPT* (DKI) mice. *n*=4/age.

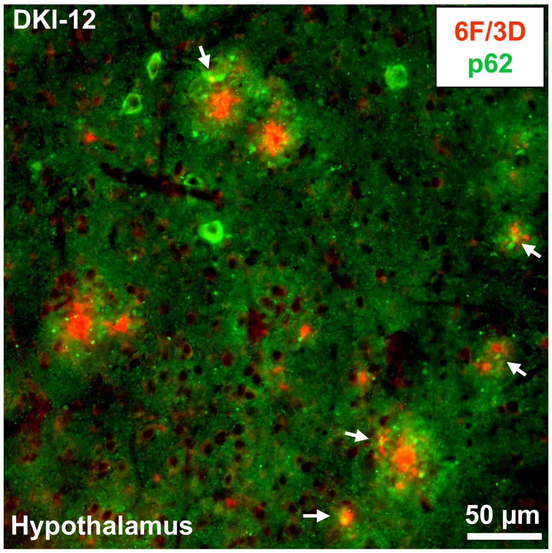


**Supplementary Fig. 10: Co-localization of Aβ with p62 in the hypothalamus.** 6F/3D (Aβ, red) and p62 (green) immunostaining was conducted in 4- and 12-month *App*$^{NL\text{-}G\text{-}F}$x*MAPT* mice (*n*=4/age). Representative image from a 12-month *App*$^{NL\text{-}G\text{-}F}$x*MAPT* mouse demonstrates co-localization of Aβ in the uncleared autophagic cargo (white arrows), and the association of p62 aggregates with plaques. These associations were rare at 4-months, considering low plaque in the region at the early-stage timepoint.

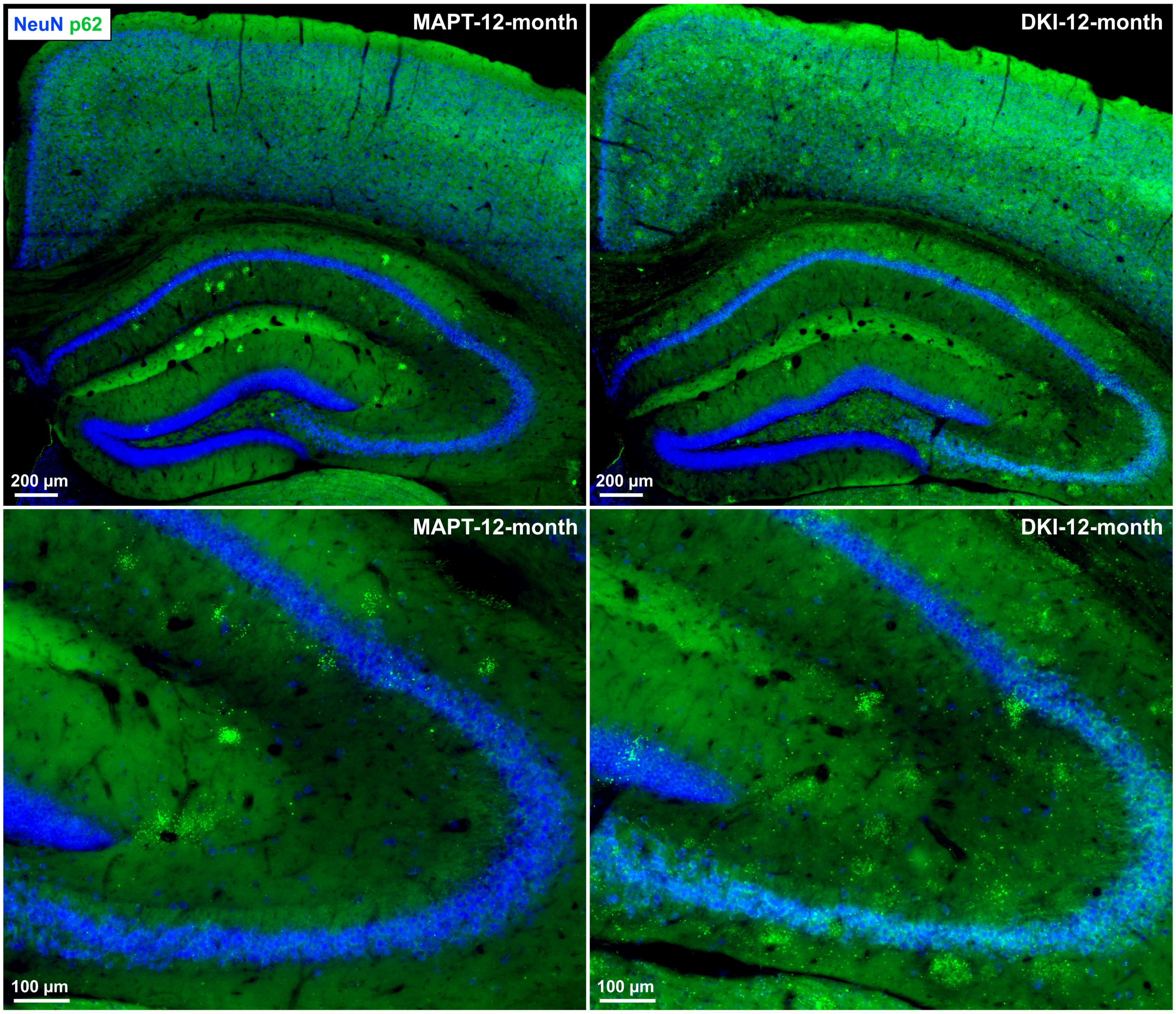


**Supplementary Fig. 11: p62 accumulation in 12-month *MAPT* and *App*$^{\text{NL-G-F}}$x*MAPT* mice.** Representative p62 (green) and NeuN (blue) immunostaining at 12-months demonstrating higher total abundance and spread of p62 burden in *App*$^{\text{NL-G-F}}$x*MAPT* (DKI) mice vs. *MAPT*s, across the neocortex and hippocampus. p62 clusters in *MAPT* mice are denser, but much less frequent, than *App*$^{\text{NL-G-F}}$x*MAPT*s. Representative images from *n*=3/sex/genotype.

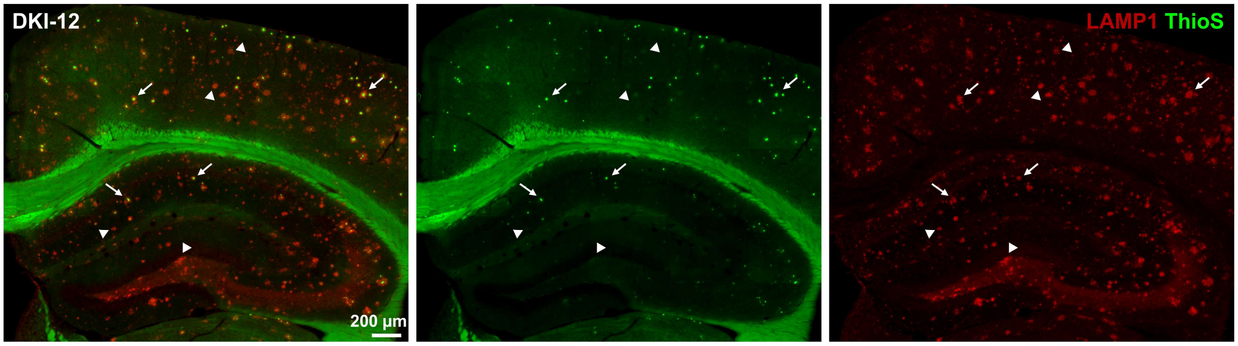


**Supplementary Fig. 12: Association of lysosomal pathology with Aβ plaques in the *App*$^{NL-G-F}$x*MAPT* hippocampus and cortex.** Similar to as observed in the hypothalamus (see **Fig. 6M**), LAMP1 (red) pathology surrounds plaques and appears prior to the formation of β-sheet detected by ThioS (green). Representative 12-month *App*$^{NL-G-F}$x*MAPT* (DKI) images demonstrate LAMP1+/ThioS+ plaques (white arrows) and LAMP1+/ThioS- formations (white arrowheads). This was similarly observed in 4-month *App*$^{NL-G-F}$x*MAPT* mice. *n*=3/age.

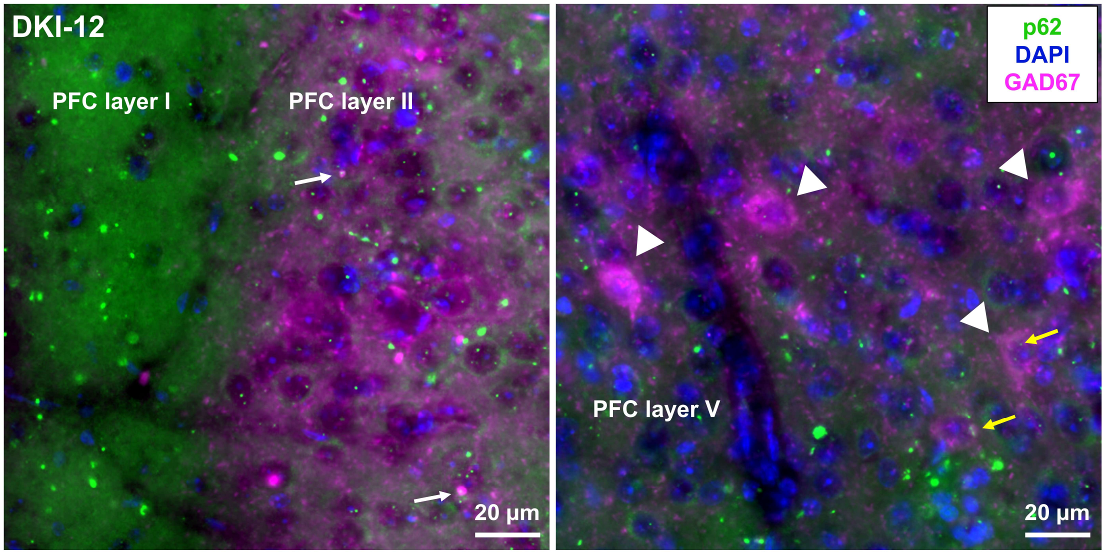


**Supplementary Fig. 13: Inhibitory neurons are relatively spared from p62 aggregates.** Immunostaining was conducted for p62 (green), GAD67 (purple) and DAPI (blue). GAD67+ inhibitory neuronal cell bodies (white arrowheads) do not co-localize with p62 in the PFC, or the lateral preoptic area of the hypothalamus (see **Fig. 8**). We did observe rare, below-threshold co-labelling of GAD67+ dendrites with p62 (white arrows), and rare, below-threshold p62 in GABAergic neuronal cytoplasm (yellow arrows), though these are likely due to normal autophagic flux and are not indicative of excessive aggregated and uncleared protein. Representative images were chosen from 12-month *App*$^{NL-G-F}$x*MAPT* (DKI) mice after looking at 8 mice across the 2 genotypes (*MAPT*, *App*$^{NL-G-F}$x*MAPT*) and 2 ages (4- and 12-months); *n*=2/genotype/age.

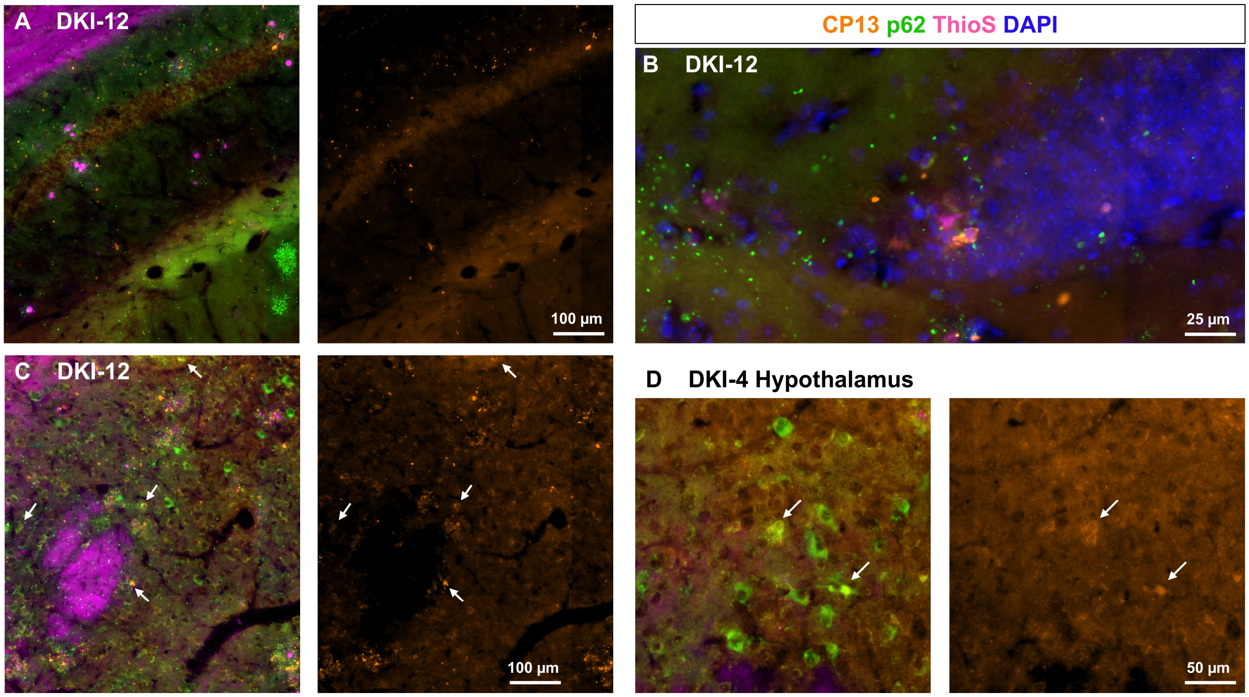


**Supplementary Fig. 14: *App*$^{NL\text{-}G\text{-}F}$x*MAPT* mice exhibit CP13+ dystrophic neurites and inclusions in the hippocampus and hypothalamus.** Phosphorylated tau was assessed using CP13 (Ser202) in the hippocampus and hypothalamus in 4- and 12-month *App*$^{NL\text{-}G\text{-}F}$x*MAPT* (DKI) mice. **A** Representative CP13 (orange), p62 (green) and ThioS (pink) staining in the hippocampus demonstrating CP13+ dystrophic neurites around plaques in 12-month *App*$^{NL\text{-}G\text{-}F}$x*MAPT*; similar results were observed at 4-months at a lesser degree. **B** CP13 cellular inclusions were also observed; image demonstrates co-localization with DAPI (blue). **C** In the 12-month *App*$^{NL\text{-}G\text{-}F}$x*MAPT* hypothalamus, CP13 pathology is present around plaques and in co-localization (non-exclusive) with cytoplasmic p62 inclusions (white arrows). **D** p62+/CP13+ inclusions (white arrows) were also observed at 4-months. *n*=3/age.

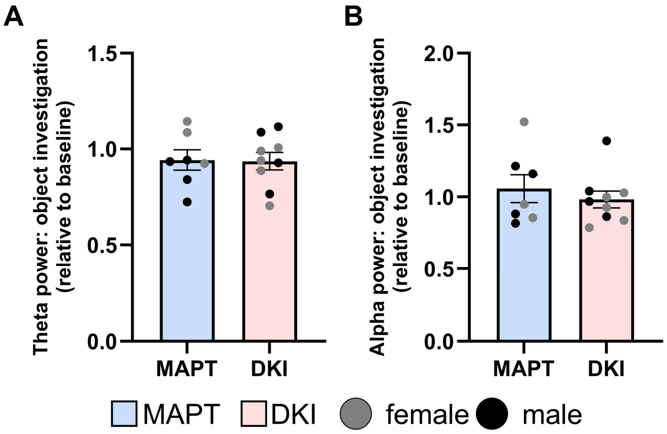


**Supplementary Fig. 15: Hippocampal theta and alpha power during object investigation.** **A** No genotype differences were detected between *MAPT* and *App*$^{NL\text{-}G\text{-}F}$x*MAPT* (DKI) mice in the theta power ratio (object investigation:baseline; unpaired t-test, t=0.0891(df=14), P=0.9303). **B** No genotype differences were detected in the alpha power ratio (object investigation:baseline; unpaired t-test, t=0.6945(df=14),

P=0.4987). Data are presented as mean+/-SEM; *n*=7-9/genotype.

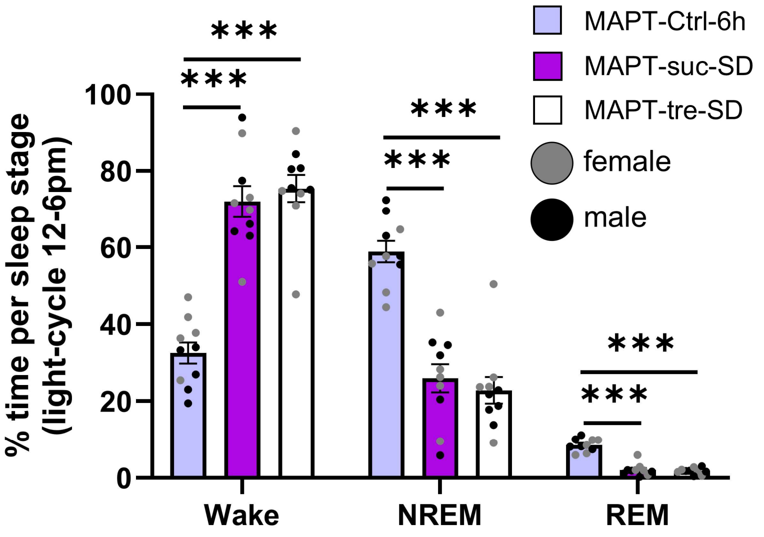


**Supplementary Fig. 16: Confirmation of sleep disruption with EEG.** Extended data for **Fig. 10**. *MAPT* mice were treated with 2% trehalose (MAPT-tre) or the control treatment with 2% sucrose (MAPT-suc) and underwent a 6-hour sleep disruption (SD) 12:00-6:00pm in the light-cycle prior to subsequent testing (see **Fig. 10**), and were compared to 12-month *MAPT* control (Ctrl) without SD (re-analysis of data from **Fig. 3**, limiting to the same timeframe as the SD). There was an overall effect of sleep stage (F(1.022,27.60)=201.7, **P<0.0001**), a trend to an SD effect (F(2,27)=2.589, P=0.0936), and a significant sleep stage*SD interaction effect (F(4,54)=42.07, **P<0.0001**). SD resulted in a significant loss of NREM and REM sleep, and increased wake, in MAPT-tre and MAPT-suc mice compared to the control (all **P<0.0001**). There was no difference between the MAPT-suc or -tre condition (wake: P=0.5332; NREM: P=0.5445; REM: P=0.6170). Data are presented as mean+/-SEM; *n*=5/sex/condition. ***P<0.001. Statistical analysis was with two-way repeated measures ANOVA, Holm-Šídák post-hoc.
